# DNA methylation and histone post-translational modification stability in post-mortem brain tissue

**DOI:** 10.1186/s13148-018-0596-7

**Published:** 2019-01-11

**Authors:** Jessica S. Jarmasz, Hannah Stirton, James R. Davie, Marc R. Del Bigio

**Affiliations:** 1 Department of Human Anatomy and Cell Science, University of Manitoba, Room 674 JBRC - 727 McDermot Avenue, Winnipeg, MB R3E 3P4 Canada; 2 Max Rady College of Medicine, University of Manitoba, Room 260 Brodie Centre - 727 McDermot Avenue, Winnipeg, MB R3E 3P5 Canada; 3 Department of Biochemistry and Medical Genetics, University of Manitoba, Room 333A BMSB, 745 McDermot Avenue, Winnipeg, MB R3E 0J9 Canada; 4 Department of Pathology, University of Manitoba, Room 401 Brodie Centre - 727 McDermot Avenue, Winnipeg, MB R3E 3P5 Canada

**Keywords:** Cortex, Human brain, Pig brain, Mouse brain, Autopsy, Post-mortem delay, Epigenetics, Histone acetylation, Histone methylation, DNA methylation

## Abstract

**Background:**

Epigenetic (including DNA and histone) modifications occur in a variety of neurological disorders. If epigenetic features of brain autopsy material are to be studied, it is critical to understand the post-mortem stability of the modifications.

**Methods:**

Pig and mouse brain tissue were formalin-fixed and paraffin-embedded, or frozen after post-mortem delays of 0, 24, 48, and 72 h. Epigenetic modifications frequently reported in the literature were studied by DNA agarose gel electrophoresis, DNA methylation enzyme-linked immunosorbent assays, Western blotting, and immunohistochemistry. We constructed a tissue microarray of human neocortex samples with devitalization or death to fixation times ranging from < 60 min to 5 days.

**Results:**

In pig and mouse brain tissue, we found that DNA cytosine modifications (5mC, 5hmC, 5fC, and 5caC) were stable for ≥ 72 h post-mortem. Histone methylation was generally stable for ≥ 48 h (H3K9me2/K9me3, H3K27me2, H3K36me3) or ≥ 72 h post-mortem (H3K4me3, H3K27me3). Histone acetylation was generally less stable. The levels of H3K9ac, H3K27ac, H4K5ac, H4K12ac, and H4K16ac declined as early as ≤ 24 h post-mortem, while the levels of H3K14ac did not change at ≥ 48 h. Immunohistochemistry showed that histone acetylation loss occurred primarily in the nuclei of large neurons, while immunoreactivity in glial cell nuclei was relatively unchanged. In the human brain tissue array, immunoreactivity for DNA cytosine modifications and histone methylation was stable, while subtle changes were apparent in histone acetylation at 4 to 5 days post-mortem.

**Conclusion:**

We conclude that global epigenetic studies on human post-mortem brain tissue are feasible, but great caution is needed for selection of post-mortem delay matched controls if histone acetylation is of interest.

**Electronic supplementary material:**

The online version of this article (10.1186/s13148-018-0596-7) contains supplementary material, which is available to authorized users.

## Background

Epigenetics represents the modification of gene expression by changes in the chemical makeup of nucleotides or the associated histone proteins, rather than alteration of the genetic code itself. Various DNA methylation changes, covalent histone tail modifications, and modulation of the enzymes responsible for epigenetic modifications are critical in neural development, aging, and neurological disease [1, 2]. Animal models have provided valuable insights into epigenetic events [3–5]. However, direct study of human brain tissue is necessary for understanding the human condition [6]. Despite this necessity, the use of human autopsy material has several inherent limitations. Variation in the circumstances before death (agonal state) including brain trauma, hypoxia, and seizures can alter the molecular constituents of tissue [7–9]. For example, human brains with ischemic injury had lower levels of histone acetylation, possibly associated with inhibited transcription of many genes [10]. Post-mortem variables include the environment after death (especially temperature), the time between death and autopsy (post-mortem delay—PMD), post-mortem artifacts such as putrefaction, method of preservation (chemical fixation vs. freezing), and storage temperature (room vs. refrigerated). In general, the differential vulnerability of DNA, RNA, proteins, and metabolites to PMD is well recognized. It is equally important to understand the post-mortem changes in epigenetic modifications.

DNA is well preserved when quickly extracted and frozen at − 80 °C, or fixed in buffered formalin for a short period (≤ 1 month) prior to paraffin-embedding [11]. Less is known about the maintenance of epigenetic modifications following death. Two chromatin immunoprecipitation (ChIP) studies of human post-mortem brains showed that DNA remained attached to histones for at least 30 h after death [12, 13]. In samples of brain from deceased adult humans that had been infused with “formalin within the first hours post-mortem” and embedded in paraffin after up to 2 months in fixative, global DNA methylation was generally preserved although gene-specific DNA methylation studies can be compromised if the tissue has been stored for decades [14]. Methylation patterns were successfully analyzed using bisulfite conversion of DNA extracted from human brains with Alzheimer’s disease and a PMD of ~ 48 h [15]. In an extreme example, methylation patterns in DNA extracted from > 10,000-year-old bone was successfully analyzed by bisulfite allelic sequencing [16]. Using immunohistochemistry (IHC), histone methylation (H3K4me2, H3K4me3, H3K27me3, H4K20me3) appears to be maintained in human cerebellum for at least 11 h post-mortem [12]. Utilizing ChIP, histone H3 trimethylated at lysine 4 (H3K4me3) and H3K27me3 were stable at various gene promotors up to 30 h post-mortem [13]. Barrachina et al. reported that histone acetylation (H3K9ac and H3K27ac) post-translational modifications (PTMs) at certain gene promotors varied substantially between 0 and 50 h post-mortem [17]. Lysine methyltransferase and acetyltransferase enzyme activities, which generate histone PTMs, were measured in 12 human post-mortem brain samples. Both enzyme activities tended to decline with increasing PMD from 5 to 100 h, although the changes were not statistically significant (possibly due to small sample size) [18]. To our knowledge, histone deacetylase (HDAC) and histone demethylase (HDM) enzymes have not been studied in the context of PMD nor have the enzymes responsible for DNA cytosine modifications (DNA methyltransferases (DNMTs), ten-eleven translocation (TETs), etc.).

We hypothesize that the delay between death and tissue preservation will affect epigenetic modifications in brain tissue. We addressed this by determining the stability of histone PTMs and DNA cytosine modifications in murine, porcine, and human brain samples after death using immunohistochemistry and biochemical methods.

## Results

### DNA integrity

Purified DNA from the seven pig brains (frontal cerebrum) electrophoresed on 0.8% agarose gels showed no smearing (i.e., no fragmentation) of the > 23,130 base pair band at any of the four PMD time points (Additional file 1). Note that 20–48% degradation of pig brain DNA has been previously reported after 120 h post-mortem [19].

### DNA methylation and hydroxymethylation

**Fig. 1:**
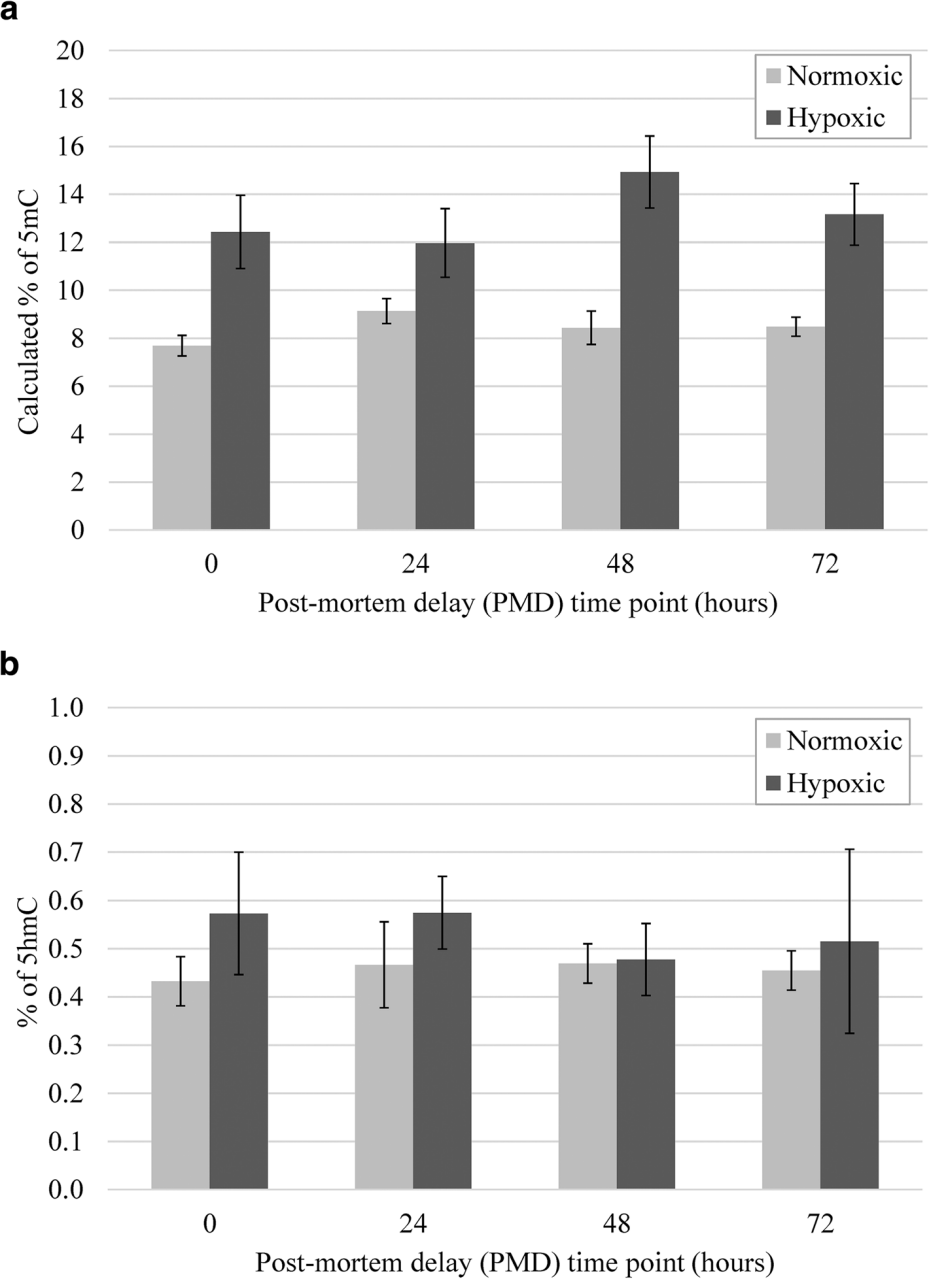
Percentage of DNA methylation (5mC; **a**) and hydroxymethylation (5hmC; **b**) in neonatal pig frontal cortex after post-mortem delays to freezing. In general, 5mC levels are higher than 5hmC levels. Temporal analysis shows no significant difference between the 0 and 72-h time points for pigs raised in normoxic (*n* = 4) and hypoxic (*n* = 3) conditions. Although the global 5mC and 5hmC levels tended to be higher in hypoxic pig brains, the change was not statistically significant (ANOVA with Dunnett’s post hoc, *p* < 0.05)

ELISA results for 5-methylcytosine (5mC) and 5-hydr-oxymethylcytosine (5hmC) are shown in Fig. 1. The percentage of 5mC in normoxic pig brain frontal cortex was approximately 8%, which coincides with previous reports of ~ 4% in adult mouse cerebral cortex and ~ 3% in newborn mouse hippocampus [20, 21]. The percentage of 5mC was stable up to 72 h post-mortem, with no statistically significant changes across PMD time points (*F*(3,27) 0.230, *p* = 0.874). This is consistent with a recent study of methylated CpGs in post-mortem rat [22], pig, and human brain [19]. The percentage of 5hmC in pig brain was approximately 0.45%, which coincides with prior reports of ~ 0.6% in adult mouse cortex and ~ 0.35% in newborn mouse hippocampus [20, 21]. The percentage of 5hmC also remained stable for at least 72 h post-mortem (*F*(3,27) 0.528, *p* = 0.667). 5mC and 5hmC levels were slightly higher in hypoxic pig brains, but the changes were not statistically significant (5mC *F*(3,27) 5.124, *p* = 0.002; 5hmC *F*(3,27) 4.256, *p* = 0.005) (Fig. 1).

### Antibody specificity

Some histone PTM antibodies can cross-react with histone PTMs other than their advertised specificity. In particular, the multiple methylation modifications on lysine residues might not be entirely distinguished. Selected antibodies were evaluated for specificity using peptide blocking immunohistochemistry and with dot blot. By immunohistochemical staining, anti-H3K27ac was appropriately blocked by its corresponding peptide (Additional file 2: Figure S1). Of the five histone methylation antibodies, only anti-H3K27me3 and anti-H3K36me3 showed the expected patterns of blocking (Table 1). Both H3K4me3 antibodies were blocked by various peptides. Note that a similar lack of specificity has been previously reported for H3K4me3 [23]. Anti-H3K27me2 was not blocked by its corresponding peptide.

**Table 1:** Summary of immunohistochemistry peptide blocking results

Peptide	Antibody						
	H3K4me3 (Abcam)	H3K4me3 (Active Motif)	H3K9me2, K9me3	H3K27me2	H3K27me3	H3K36me3	H3K27ac
H3K4me	[+]	[+]					
H3K4me2	[++]	[++]		−			
H3K4me3	+++	+++				−	
H3K9me			[+]				
H3K9me2							
H3K9me2, H3K9me3			++				
H3K9me3	[+]	[++]			−	−	
H3K27me				−	−		
H3K27me2				[−]	−		
H3K27me3	[+]	[+]		−	+++	−	
H3K36me						−	
H3K36me2				−		−	
H3K36me3					−	+++	
H3K27ac				−			+++
H4K5ac							−
Summary	Fairly specific	Fairly specific	Fairly specific	Not specific	Specific	Specific	Specific

*+++* complete block, *++* partial block, *+* minimal block, *−* no block

A blank cell means not applicable and square brackets [ ] represent an unexpected result

Antibodies tested via dot blot showed similar results (Table 2 and Additional file 2: Figure S2). Antibodies to total histone H3, H3panAc, H3K27me3, H3K14ac, H3K27ac, and H4K5ac bound only their respective peptides. Anti-H4K12ac bound its corresponding peptide strongly and H4K5ac weakly. Anti-H3K4me3 bound all peptides blotted on the membrane. Anti-H3K36me3 bound the majority of the peptides. Anti-H3K27me2 bound all but one peptide. Anti-total H4 bound none of the peptides, but it did bind calf thymus total histone mix.

**Table 2:** Summary of dot blot evaluation of antibody specificity

Peptide	Antibody											
	Total H3	Total H4	H3K27ac	H3K14ac	H4K5ac	H4K12ac	H3panAc	H3K4me3 (Abcam)	H3K4me3 (Active Motif)	H3K27me2	H3K27me3	H3K36me3
Histone mix		Strong										None
Unmodified H3	Strong	None	None	None			None		[Weak]		None	
Unmodified H4	None	[None]			None	None						
H3K4me								[Weak]	[Weak]			
H3K4me2								[Weak]	[Strong]	[Weak]		
H3K4me3								Strong	Strong			[Weak]
H3K9me	None											
H3K9me2		None								[Weak]		
H3K9me3								[Weak]	[Weak]		None	
H3K27me										None	None	
H3K27me2										[Weak]	None	
H3K27me3	None							[Weak]	[Strong]	Strong	Strong	[Strong]
H3K36me												None
H3K36me2										[Weak]		[Strong]
H3K36me3								[Weak]			None	Strong
H3K14ac			None	Strong	None	None	Strong					
H3K27ac	None	None	Strong	None	None	None	Weak					
H4K5ac	None	None			Strong	[Weak]	None					
H4K12ac		None	None	None	None	Strong	None					
Summary	Specific	Specific*	Specific	Specific	Specific	Fairly specific	Specific	Fairly specific	Not very specific	Fairly specific	Specific	Fairly specific

“Strong” indicates that the antibody bound to the peptide with strong affinity, “weak” indicates that the antibody bound but with weak affinity, and “none” indicates that the antibody did not bind to the peptide. A blank cell means not applicable, and square brackets [ ] represent an unexpected result

*A discussion with the manufacturer suggested the unmodified H4 peptide may have been a bad or incorrect batch

### Histone integrity

**Fig. 2:**
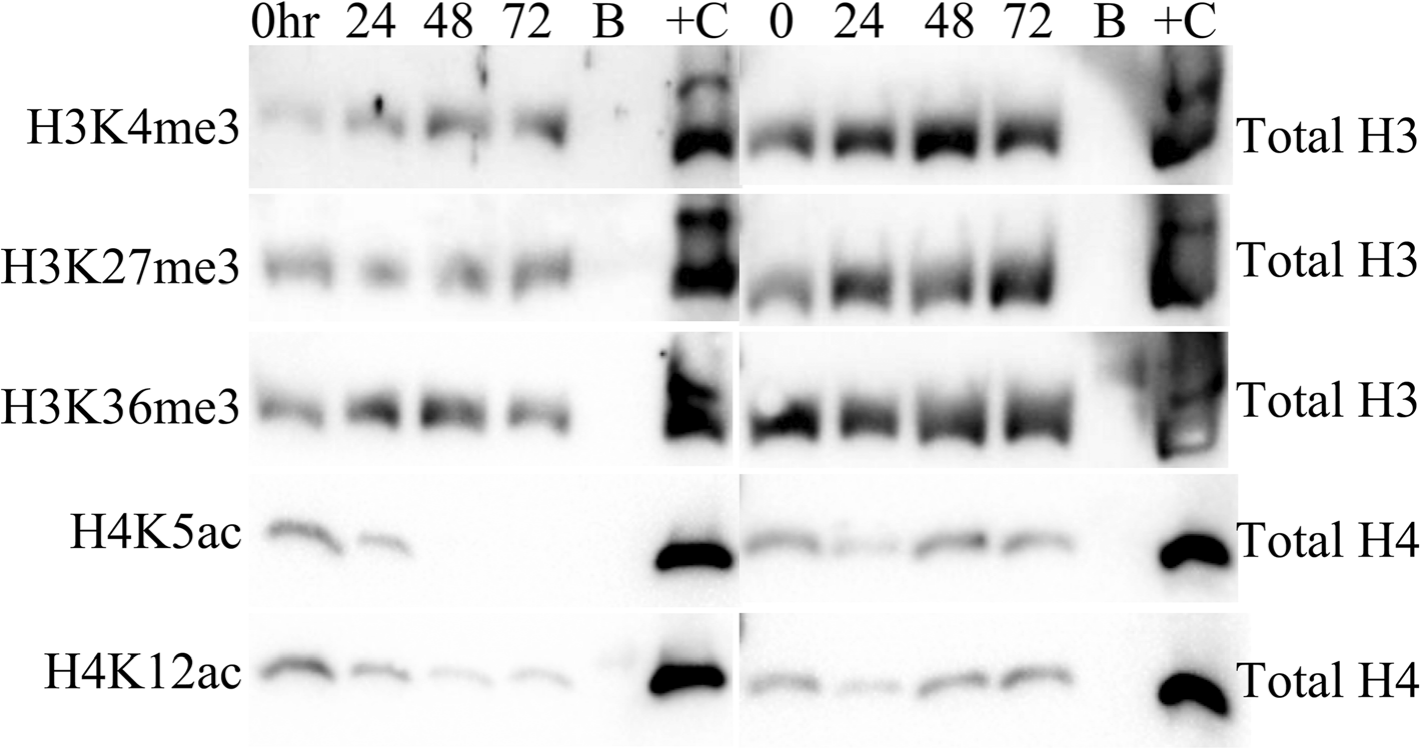
Western blots of neonatal pig (normoxic) brain samples for histone PTMs (left) and corresponding total histone (right). Histone methylation is stable across all post-mortem delay (0–72 h) time points while histone acetylation diminishes with increasing PMD. hr,.hour; B, blank lane, +C, positive control (calf thymus histone mix)

**Fig. 3:**
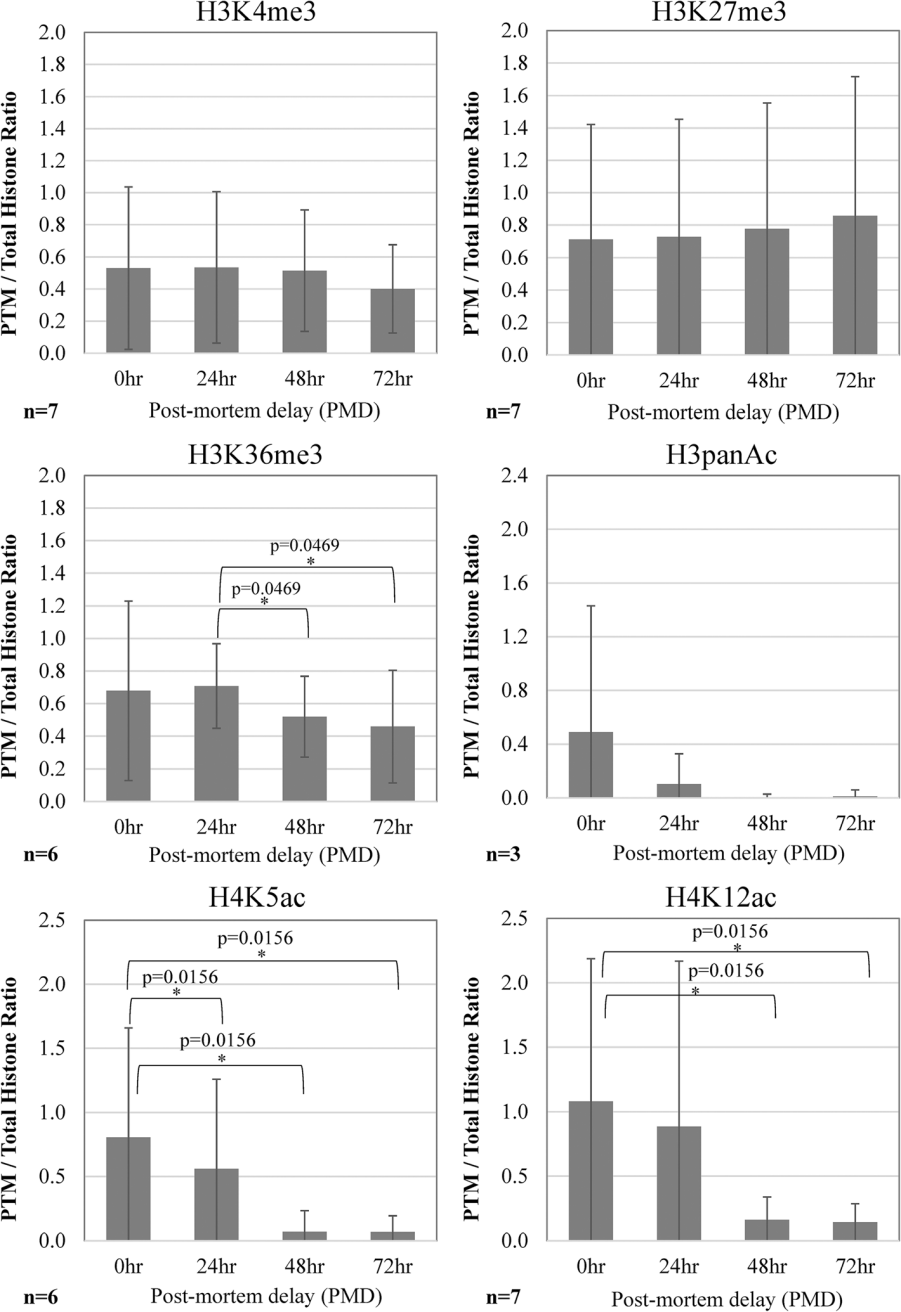
Quantitation of histone epigenetic modifications on Western blots of neonatal pig brain. There were no differences between normoxic and hypoxic samples; therefore, all data were pooled (*n* = 7) and densitometric band “quantity” displayed (mean ± 95% confidence intervals). H3K4me3 and H3K27me3 remained stable ≥ 72 h post-mortem. H3K36me3 was stable for 24 h but decreased significantly by 48 h. H3panAc decreased at 24 h and was negligible by 48 h; the difference was not significant because the initial values were low with wide variation. H4K5ac and H4K12ac were significantly decreased by 48 h. *p* values significant at *p* < 0.05 that passed the Benjamini-Hochberg correction are shown on the graphs

In pig brain, total histone H3 and histone H4 levels were stable up to 72 h post-mortem (Fig. 2 and Additional file 3). H3K4me3 and H3K27me3 histone methylation modifications also remained stable up to 72 h post-mortem, while H3K36me3 showed a decline by 48 h (*p* < 0.05) (Figs. 2 and 3). Histone acetylation tended to decline by 24 h post-mortem and was significantly reduced by 48 h (Fig. 3). The quantities of all histone PTMs were higher in normoxic pig brain than in hypoxic pig brain except for H3K36me3, but this was not statistically significant (results not shown). For detailed histone integrity results, see Additional file 4.

### Post-mortem stability of immunoreactivity

**Fig. 4:**
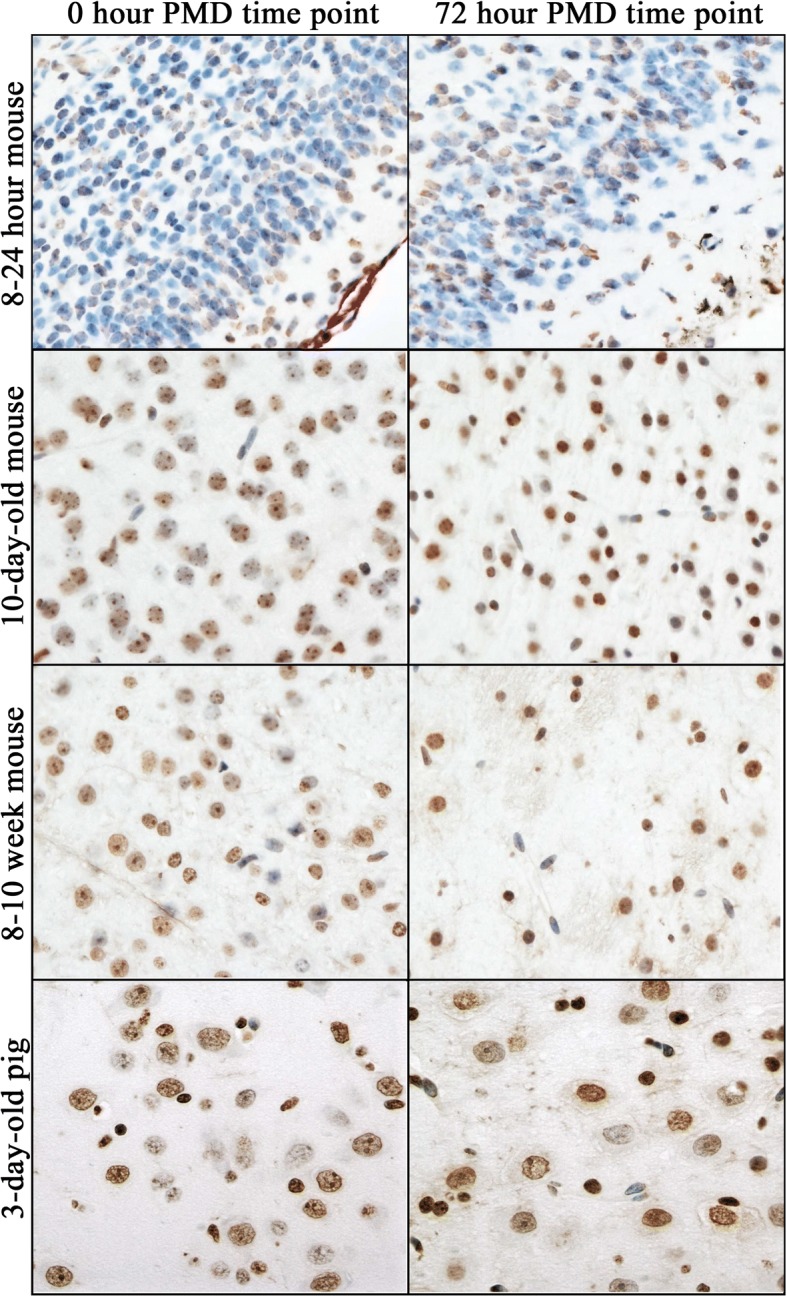
Immunohistochemical detection of 5 methylcytosine (5mC) in mouse and neonatal pig neocortex. Labeling is strongest in the large round nuclei of neurons in mouse brain; note that immature neurons in newborn mouse are usually negative. In pig brain, smaller glial nuclei are also labeled. Elongated nuclei of endothelial cells are generally negative. There is no difference between 0 (left) and 72 (right) hours post-mortem in any of the animal groups. Images taken at × 400 magnification. DAB detection of antibody (brown) and hematoxylin counterstain (blue)

**Fig. 5:**
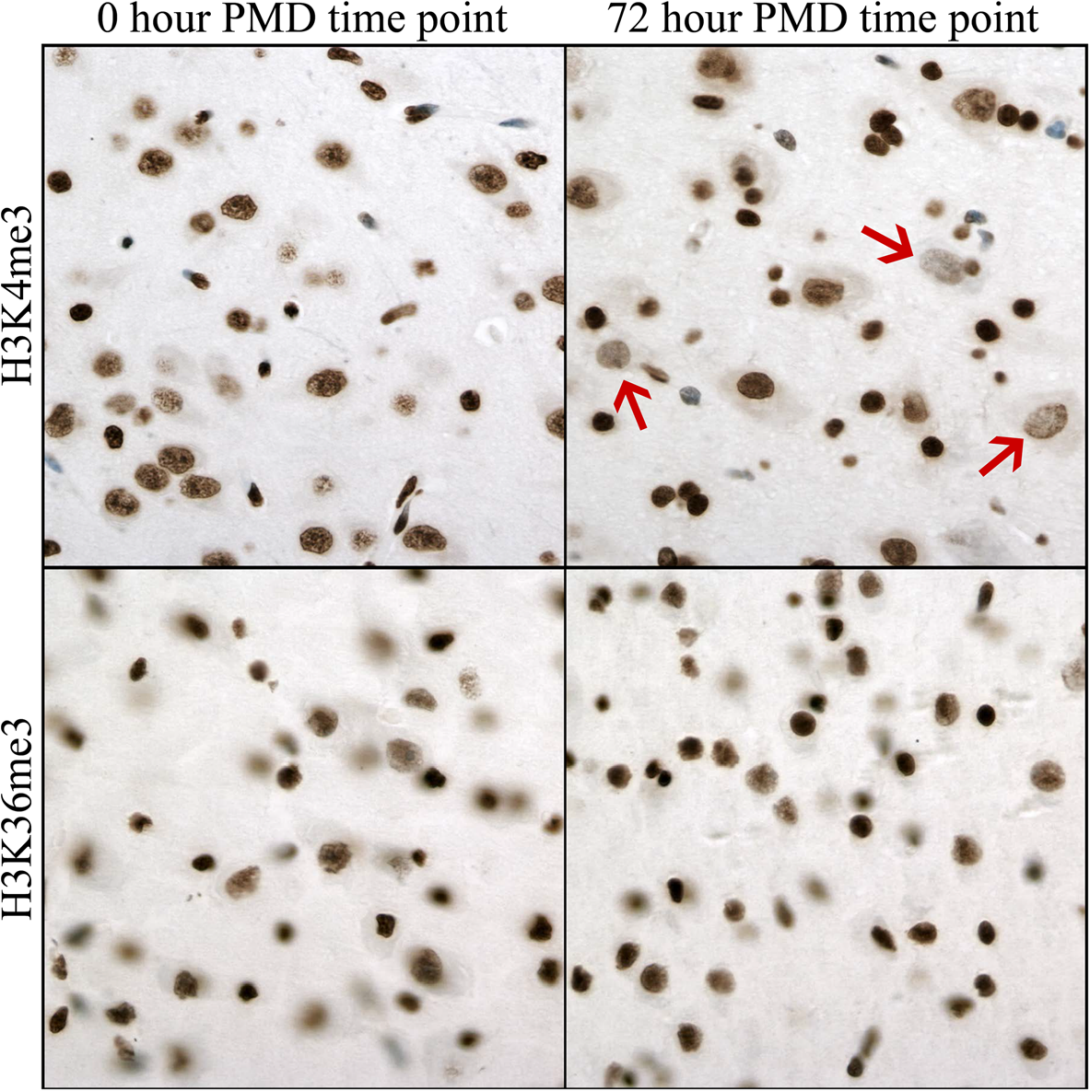
Immunohistochemical detection of histone 3 methylation PTMs (H3K4me3, H3K36me3) in neonatal pig neocortex. In control (0 h) brain, the nuclei of almost all cell types are positive except rare endothelial cells. Photomicrographs show that some large neurons (red arrows) have a minor decrease in intensity of immunoreactivity for H3K4me3 at 72 h. Images taken at × 400 magnification. DAB detection of antibody (brown) and hematoxylin counterstain (blue)

**Fig. 6:**
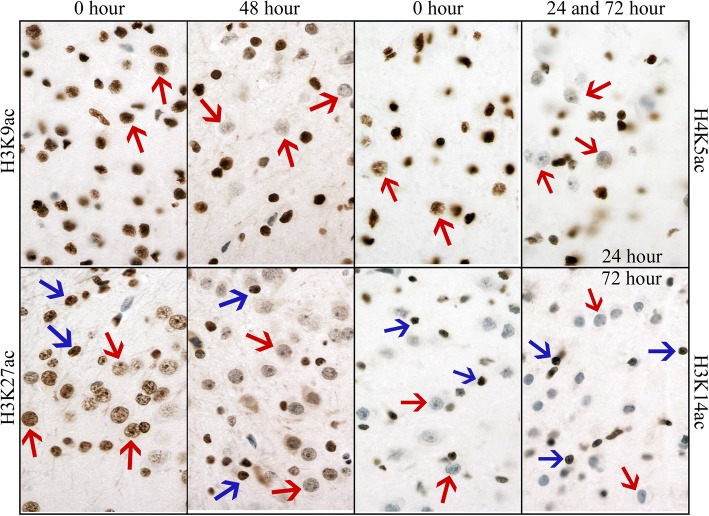
Immunohistochemical detection of histone 3 acetylation PTMs in neonatal pig neocortex. In control brain (0 h) anti-H3K9ac strongly labels nuclei of large neurons (red arrows) and smaller glial cells. Anti-H3K27ac and anti-H4K5ac label smaller glial nuclei (blue arrows) more intensely than those of neurons (red arrows). Anti-H3K14ac labels only smaller glial nuclei. Photomicrographs show a loss of immunoreactivity in large neurons but not glial cells (note different time points at which change was first observed). Images taken at × 400 magnification. DAB detection of antibody (brown) and hematoxylin counterstain (blue)

**Fig. 7:**
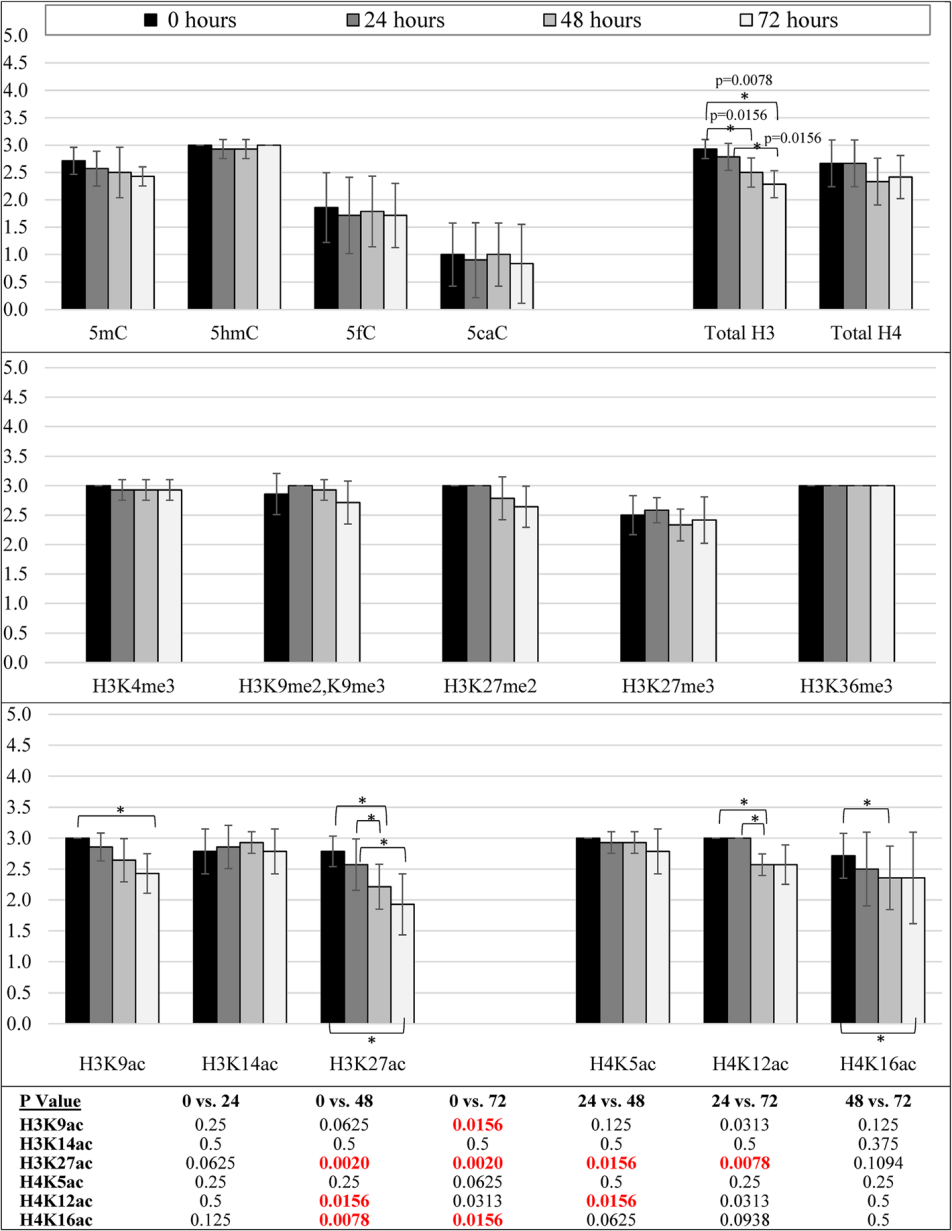
Bar graphs showing the semiquantitative intensity scores (mean ± 95% confidence intervals; maximum 3) for all epigenetic modification antibodies used in neonatal pig neocortex. DNA cytosine modifications, total histone H4, all histone methylation PTMs, H3K14ac, and H4K5ac and showed stable immunoreactivity up to 72 h post-mortem. H3K9ac was stable up to 48 h post-mortem. Total histone H3, H3K27ac, H4K12ac, and H4K16ac all showed a significant decline by 48 h post-mortem. *p* values for all statistical comparisons shown at bottom. A *p* value < 0.05 that is not bolded in red did not pass the Benjamini-Hochberg correction

Among both pig and mouse brain samples, DNA cytosine modifications, all histone methylation PTMs, H3K14ac, H4K5ac, and total histone H4 showed stable immunoreactivity up to 72 h post-mortem (Figs. 4, 5, 6, and Additional file 5). In pig brain (temporal and parietal cortices), H3K9ac was stable up to 48 h post-mortem (Figs. 6 and 7). H3K27ac, H4K12ac, H4K16ac, and total histone H3 were stable up to 24 h post-mortem (Figs. 6 and 7). In mouse dentate gyrus, H4K5ac showed a downward trend (not statistically significant) with increasing PMD (Additional file 6). H3K9ac was stable up to 48 h post-mortem (Additional file 6). H3K27ac, H4K12ac, and H4K16ac were stable only up to 24 h post-mortem (Additional file 6).

**Fig. 8:**
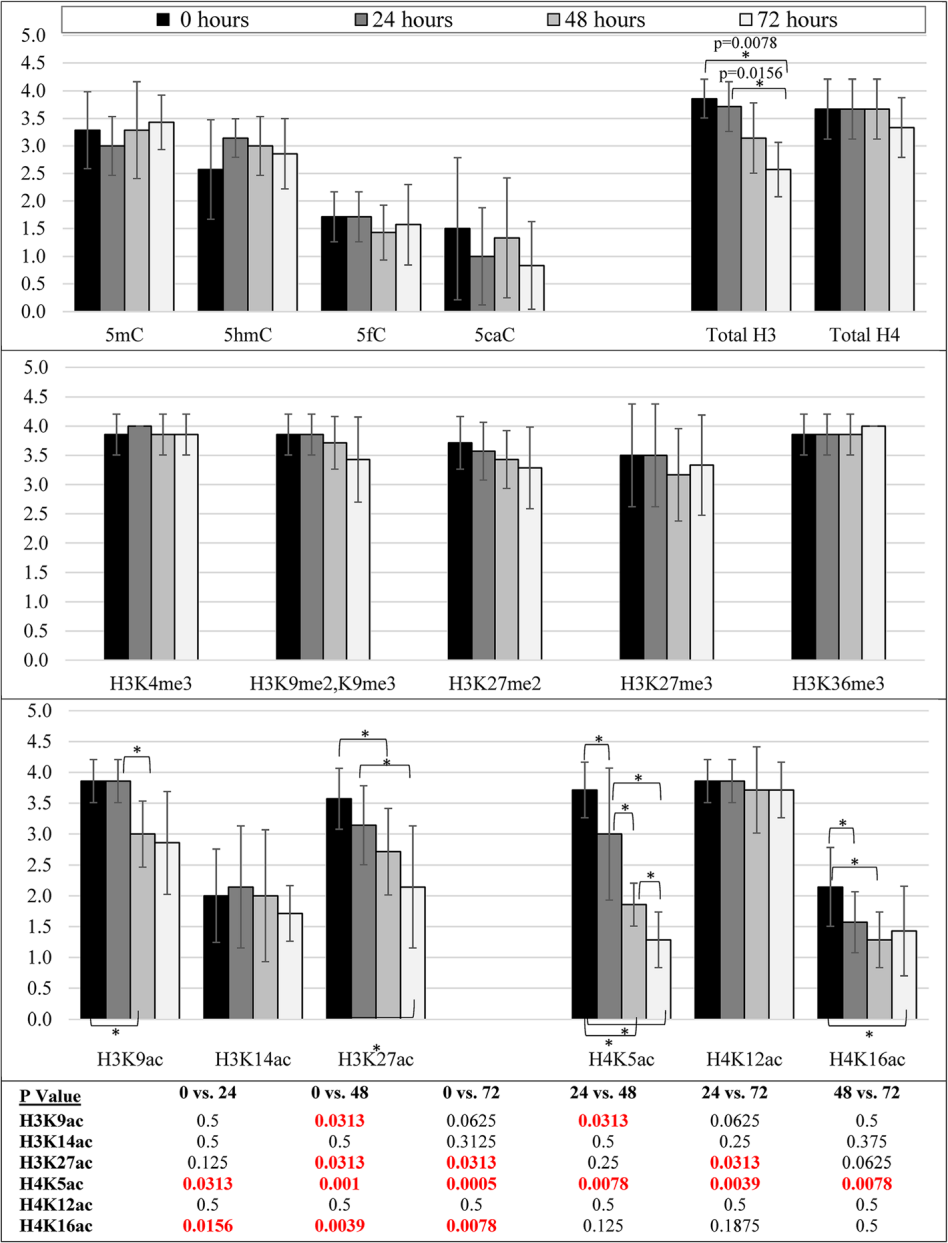
Bar graphs showing the semiquantitative proportion scores (mean ± 95% confidence intervals; maximum 4) for all epigenetic modification antibodies used in neonatal pig neocortex. DNA cytosine modifications, total histone H4, all histone methylation PTMs, H3K14ac, and H4K12ac were stable up to 72 h post-mortem. Total histone H3 declined after 48 h post-mortem and was significantly decreased at 72 h. H3K9ac was stable at 24 h and decreased significantly thereafter. H3K27ac, H4K5ac, and H4K16ac had all declined by 24 h and were significantly decreased by 48 h. *p* values for all statistical comparisons shown at bottom. A *p* value < 0.05 that is not bolded in red did not pass the Benjamini-Hochberg correction

In pig brain temporal and parietal cortices, the (semiquantitative) proportion of cells labeled with antibodies to DNA cytosine modifications, all histone methylation PTMs, H3K14ac, H4K12ac, and total histone H4 appeared stable up to 72 h post-mortem (Table 3; Figs. 4, 5, 6, and 8 and Additional file 5). Total histone H3 declined after 48 h post-mortem in all cell types (Table 3; Additional file 5 and Fig. 8). H3K9ac and H3K27ac were stable up to 24 h; thereafter, loss of staining was observed in large neuronal nuclei (Table 3; Figs. 6 and 8). H4K5ac and H4K16ac had already declined by 24 h in neuronal nuclei (Table 3; Figs. 6 and 8).

**Table 3:** Loss of epigenetic mark immunoreactivity among different cell types in newborn pig temporal/parietal cortices

		Nuclear morphology				
Epigenetic modification	Observed change (time point)	Large round	Intermediate	Small round	Small irregular/longer	Endothelial/smooth muscle
5mC	72 h	Minimal	Minimal	Minimal		
5hmC	72 h			Minimal		
5fC	72 h					
5caC	72 h	n/a				
H3K4me3	72 h	Minimal			Minimal	Minimal
H3K9me2, K9me3	72 h	Minimal			Minimal	
H3K27me2	72 h	Minimal				
H3K27me3	72 h	Minimal			Minimal	
H3K36me3	72 h					Minimal
H3K9ac	48 h	Moderate				
H3K14ac^A^	72 h	n/a	Minimal	Minimal	Minimal	
H3K27ac	72 h	Moderate	Minimal	Moderate		
H4K5ac	48 h	Moderate	Minimal		Minimal	
H4K12ac	48 h	Minimal			Minimal	
H4K16ac	48 h	Minimal				
Total H3	48 h	Minimal	Minimal	Minimal	Minimal	Minimal
Total H4	72 h				Minimal	

^A^Large nuclei were not positively stained at 0 h

A blank cell represents no change

**Fig. 9:**
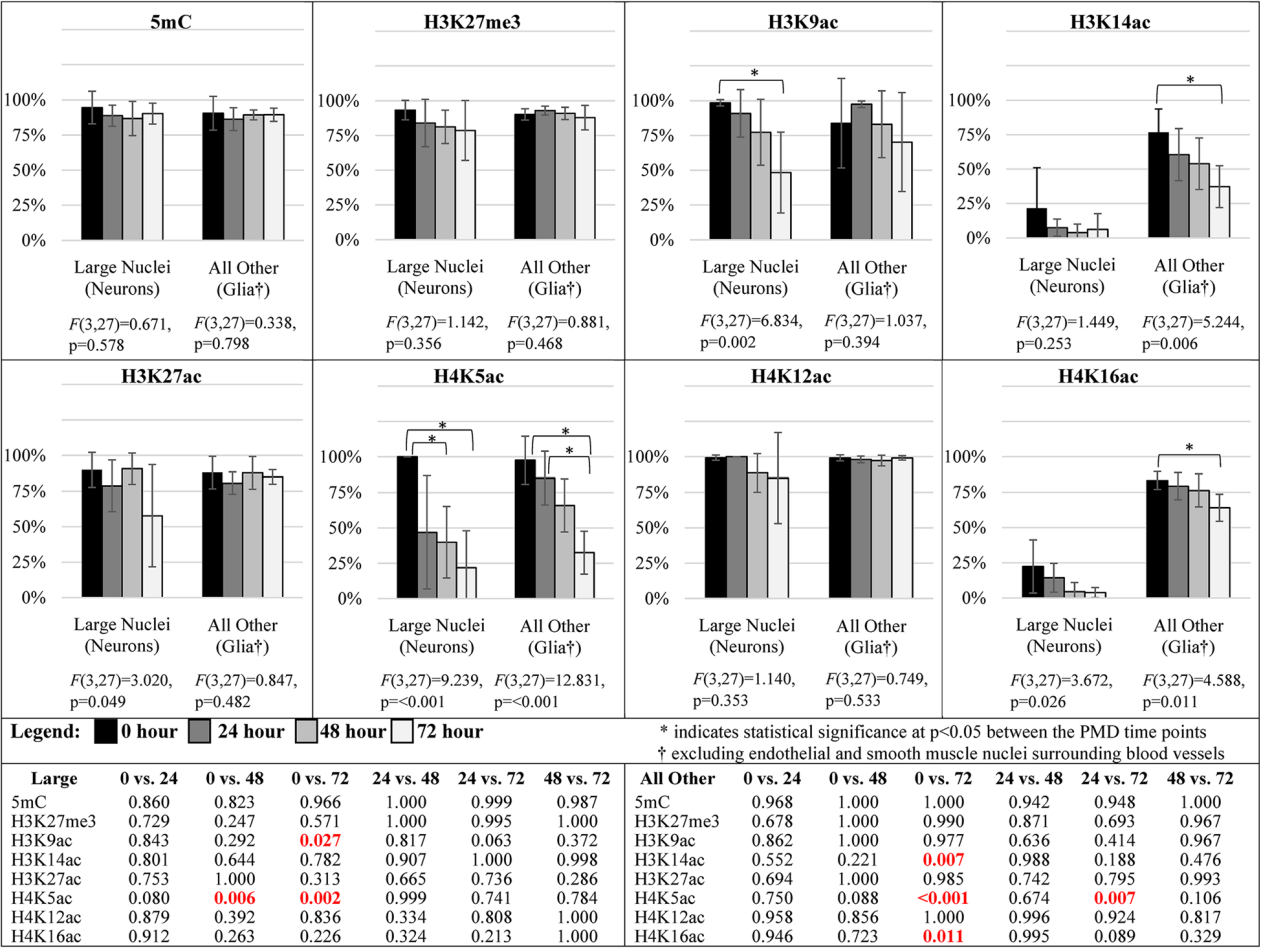
Bar graphs showing the quantitative analysis (mean ± 95% confidence intervals; maximum 100%) for nuclear immunostaining of selected epigenetic marks in neonatal pig neocortex. ANOVA *F* statistic and *p* values are also shown. Data are shown separately for large round nuclei (neurons) and smaller irregular nuclei (glial cells and/or interneurons). 5mC, H3K27me3, H3K27ac, and H4K12ac were stable at all post-mortem times to 72 h in both populations. H3K9ac declined in neurons, but not glial cells. H3K14ac and H4K16ac declined in glial cells, but only rare neurons were labeled in the control state (0 h). H4K5ac was lost in both neurons and glial cells. *p* values for all statistical comparisons shown at bottom and were subjected to the Dunnett’s post hoc test (*p* < 0.05)

Where the semiquantitative evaluation indicated changes in the immunohistochemical detection of PTMs, a quantitative evaluation was used to validate the results (Fig. 9). On images of pig neocortex, the intensity of nuclear immunoreactivity was measured (results not shown) and the proportions of immunoreactive cells were counted in two morphologic populations: large round nuclei (neurons) and smaller round to ovoid nuclei (glial cells and small interneurons; elongated nuclei of endothelial and smooth muscle cells were excluded). Because DNA cytosine modifications and histone methylation PTMs are stable up to 72 h post-mortem, we did a quantitative validation for only 5mC and H3K27me3. With 5mC and H3K27me3 immunostaining, neither cell population exhibited differences over progressive PMD as determined by ANOVA with Dunnett’s post hoc test for multiple comparisons. Among the acetyl marks, only H3K9ac showed a statistically significant decrease in neurons, but not in glial cells. H4K5ac demonstrated decreases in both neurons and glia by 48 h post-mortem. H3K14ac and H4K16ac were expressed at very low levels in neurons to begin with. Statistically significant post-mortem decreases of these two marks were demonstrated in glial nuclei (Fig. 9).

Reflecting their cell maturation-dependent state, regional localization of most epigenetic marks differed between the three age groups of mice (Fig. 4; Additional file 5). However, with respect to post-mortem stability, immunostaining of mouse brains yielded essentially the same result as the pig brains. The three age groups showed similar patterns of stability and were therefore pooled for analysis. DNA cytosine modifications, histone methylation, H3K14ac, and H4K12ac were stable from 0 to 72 h post-mortem (Fig. 4; Additional files 5 and 6). Total histone H3 and H4 declined after 48 h (Additional file 6); the loss did not appear to be cell type specific (Table 4). H3K9ac and H3K27ac were stable up to 24 h post-mortem (Additional file 6); after that, loss of immunoreactivity was seen in large neurons (Table 4). H4K5ac and H4K16ac declined by 24 h in large neurons (Additional file 6; Table 4). A limited analysis of 96 h PMD was conducted on two ~ 13-week mouse brains. Immunoreactivity for 5mC, 5hmC, 5fC, 5caC, H3K4me3, H3K27me3, and H3K36me3 was unaltered. There was a slight decrease in the intensity of H3K9me2, K9me3, and H3K27me2 immunoreactivity.

**Table 4:** Loss of epigenetic mark immunoreactivity among different cell types in young adult mouse hippocampal dentate gyrus

		Nuclear morphology				
Epigenetic modification	Observed change (time point)	Large round	Intermediate	Small round	Small long/irregular	Endothelial/smooth muscle
5mC	72 h	Minimal				
5hmC	72 h					
5fC	72 h					
5caC	72 h	Minimal				
H3K4me3	72 h				Minimal	Minimal
H3K9me2, K9me3	72 h	Minimal			Minimal	
H3K27me2	72 h	Moderate				
H3K27me3	72 h	Minimal			Minimal	Minimal
H3K36me3	72 h				Minimal	
H3K9ac	48 h	Moderate			Minimal	
H3K14ac^A^	72 h	Minimal				Minimal
H3K27ac	72 h	Minimal			Minimal	
H4K5ac	48 h	Moderate				
H4K12ac	48 h	Moderate				
H4K16ac	48 h	n/a				
Total H3	48 h	Minimal	Minimal			
Total H4	72 h	Minimal	Minimal		Minimal	

^A^ Large nuclei were not positively stained at 0 h

A blank cell represents no change

There were no differences in the pig brain samples cooled after 6–8 h at room temperature compared to those cooled immediately (results not shown). There were no obvious differences between normoxic and hypoxic pig brains (results not shown). Of the 15 epigenetic modifications assessed by immunostaining for overfixation (using the 24 h post-mortem pig brain samples), 8 were slightly affected by overfixation. H3K27ac and H4K5ac started to show minor decreases in immunoreactivity if stored for 5 weeks in formalin prior to paraffin embedding. H3K9ac and H3K14ac started to show minor decreases in immunoreactivity when fixed for 6 weeks. 5mC, 5hmC, H3K9me2/K9me3, and H3K27me2 started to show changes when fixed for 12 weeks. Overall, the minor decrease in immunoreactivity was primarily seen in large neuronal nuclei.

### Human brain tissue microarray

**Fig. 10:**
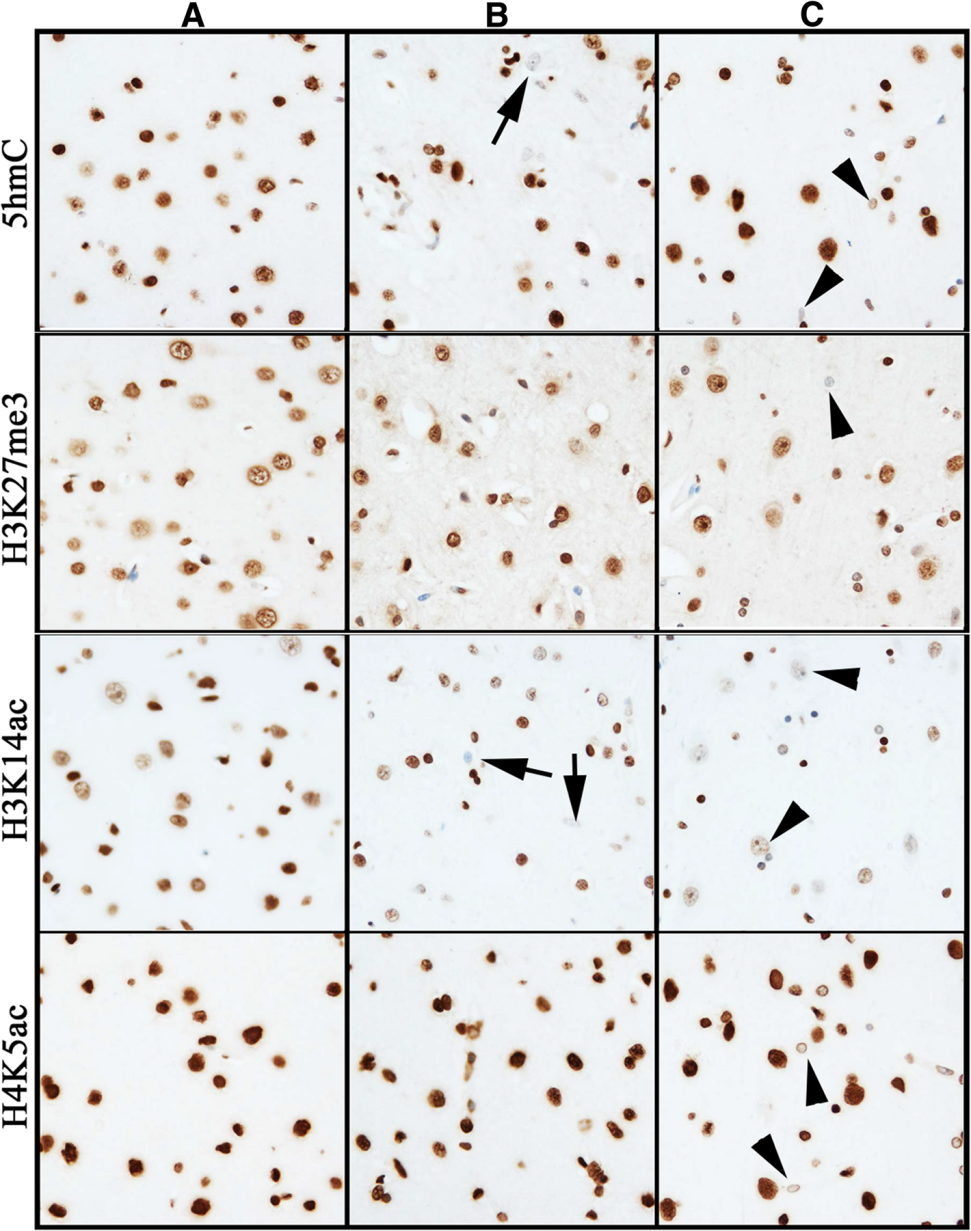
Representative immunohistochemical detection of epigenetic marks in human neocortex tissue array. Column **a** shows control temporal lobe obtained from a nonpathological surgical specimen that was fixed < 1 h after devitalization. Column **b** shows a frontal lobe specimen from a person who survived in a coma for 7 days after a severe hypoxic insult and whose autopsy (i.e., delay to fixation) was performed 31 h after death. Column **c** shows a nonpathological frontal lobe specimen from a person who died immediately after trauma and whose autopsy was performed 4 days after death. For all antibodies shown, in the control tissue almost all nuclei (except for those of scattered endothelial cells) are immunoreactive. 5mC immunoreactivity (top row) was reduced in some hypoxic neurons (arrow) and some small nuclei after prolonged delay to fixation (arrowhead). H3K27me3 (second row) immunoreactivity was unchanged in hypoxic brain (although cell shapes were altered). After prolonged delay to fixation, a few small nuclei were negative (arrowhead). H3K14ac (third row) immunoreactivity was diminished in approximately half the cells in hypoxic brain (arrows) and many neurons in the delayed fixation brain (arrowheads). H4K5ac (fourth row) immunoreactivity was unchanged in hypoxic brain but showed diminished intensity in the delayed fixation brain (arrowheads). Images taken at × 200 magnification. DAB detection of antibody (brown) and hematoxylin counterstain (blue)

The two surgical samples (temporal cortex), which were fixed < 1 h after devitalization, exhibited strong immunoreactivity using all epigenetic modification antibodies. In the autopsy samples (frontal cortex), almost all epigenetic modifications tested were stable up to 4 days post-mortem. Acetyl marks H3K9ac, H3K27ac, H4K5ac, and H4K12ac showed apparently greater stability in the human brain than in pig and mouse brain. Sample 12 exhibited a decline in immunoreactivity for all six acetylation marks. The body of the deceased had been at room temperature for ≤ 2 days before being stored at 4 °C for 3 days prior to autopsy. The two samples from brains with hypoxic damage showed minor decreases in intensity and proportion of positive cells in all but four epigenetic modifications (5fC, H3K27me3, H4K5ac, and H4K12ac) (Fig. 10 and Table 5).

**Table 5:** Changes in epigenetic mark immunoreactivity—human neocortex tissue array samples

	Normal			Subacute hypoxic changes
Epigenetic modification	3 days post-mortem	4 days post-mortem	5 days post-mortem	31–43 h post-mortem
5mC	No change	No change	No change	Cytoplasm leakage
5hmC	No change	No change	No change	Few (−) small nuclei
5fC	No change	No change	Moderate decrease; glial and neuronal	No change
5caC	No change	No change	Moderate decrease; neuronal	Few small nuclei are (−)
H3K4me3	Minor cytoplasmic labeling	Minor decrease, neuronal	Minor decrease, neuronal	Cytoplasm leakage
H3K27me3	No change	Few (−) glial cells	No change	No change
H3K36me3	No change	Minor decrease; glial and neuronal	Minor decrease; glial and neuronal	Minor decrease; neuronal
H3panAc	No change	Minor decrease; glial and neuronal	Minor decrease; glial and neuronal	Minor decrease; glial and neuronal
H3K9ac	No change	Minor decrease; glial	Minor decrease; glial and neuronal	Few small nuclei are (−)
H3K14ac	Minor decrease; glial and neuronal	Minor decrease; glial Moderate decrease; neuronal; few (−) neurons	Minor decrease; glial, Moderate decrease; neuronal; few (−) neurons	Minor to moderate decrease; glial and neuronal; few (−) neurons
H3K27ac	Minor decrease; glial	Few small nuclei are (−)	Few small nuclei are (−)	Few small nuclei are (−)
H4K5ac	No change	Moderate decrease; glial and neuronal	Few small nuclei are (−)	No change
H4K12ac	No change	No change	No change	No change

Note: There were no obvious differences between the specimens obtained surgically and fixed < 1 h after devitalization and the autopsy samples that were fixed ≤ 2 days after death, unless there were also changes due to hypoxic-ischemic brain damage

## Discussion

A summary of the immunohistochemical labeling stability of epigenetic modifications is shown in Table 6. Overall, we conclude that DNA cytosine and histone methylation modifications, in addition to a range of additional antibodies used to evaluate brain cell types (Additional file 7), are stable ≥ 48 h and usually ≥ 72 h post-mortem in human, pig, and mouse brain. By contrast, histone acetylation modifications are generally not stable with increasing post-mortem delay. Furthermore, immunohistochemistry seemed to be more sensitive than Western blotting for the global detection of H3K9ac, H3K27ac, H4K5ac, and H4K12ac.

**Table 6:** Summary of the immunoreactivity stability of histone acetylation, histone methylation, and DNA cytosine modifications in brain samples*

Epigenetic modification	IHC	IHC—immunoreactivity		IHC—proportion		IHC—counts		Western blot
	Human—Cx	Pig—Cx	Mouse—DG	Pig—Cx	Mouse—DG	Neurons	Glia	Pig—Cx
5mC	120	72	72	72	72	72	72	n/a
5hmC	120	72	72	72	72	–	–	n/a
5fC	96	72	72	72	72	–	–	n/a
5caC	96	72	72	72	72	–	–	n/a
H3K4me3	72	72	72	72	72	–	–	72
H3K9me2/K9me3	nd**	72	72	72	72	nd**	nd**	No signal**
H3K27me_3_	72	72	72	72	72	72	72	72
H3K36me_3_	72	72	72	72	72	–	–	48
H3K9ac	72	48	48	24	24	48	72	Wrong mw
H3K14ac	53	72	72	72	72	72	48	Low signal
H3K27ac	53	24	24	24	24	72	72	Wrong mw
H4K5ac	72	72	48	24	< 24	24	48	< 24
H4K12ac	120	24	24	72	72	72	72	24
H4K16ac	nd	24	24	24	< 24	72	48	No signal
Total H3	nd	24	72	48	48	–	–	72 h
Total H4	nd	72	72	72	48	–	–	72 h
H3panAc	72	nd	nd	nd	nd	nd	nd	24 h

*IHC* immunohistochemistry, *Cx* neocortex, *DG* dentate gyrus of hippocampus, *nd* not done, *n/a* not applicable; *−* not counted; *mw* molecular weight *Numeric values represent hours post-mortem to formalin fixation (or freezing for Western blots) at which there are no differences compared to samples that were fixed or frozen immediately after death

**H3K9me2,K9me3 antibody was discontinued; H3K9me2 replacement antibody could not be optimized

Although we found that DNA, global DNA methylation, and global DNA hydroxymethylation were stable ≥ 72 h post-mortem, other studies have indicated that some degradation can occur earlier. In a comet assay of cells isolated from the brains of mice that were killed and stored at room temperature, DNA degradation was apparent at 6 h and progressed to substantial levels by 72 h [24]. In a study of pig brains that remained in the skull at room temperature until the desired PMD time points were reached, up to 48% of DNA was degraded by 120 h post-mortem, and there was increased variance in DNA methylation [19]. In a study of neonatal and adult rat cerebellum left at room temperature for 0, 5, and 9 h post-mortem, there was a significant decrease in global 5mC at 9 h in the adult brains (2.9 ± 0.7% vs. 3.7 ± 0.6% in control), but no change in the neonates [25]. Not surprisingly, storage at cool temperature after death is an important factor, possibly because enzyme kinetics associated with autolysis are slowed.

We must explain the post-mortem degradation of histone acetylation modifications. The main enzymes involved in the removal of acetyl groups from lysine residues are histone deacetylases (HDAC) and sirtuins. Sirtuins are NAD+-dependent nuclear proteins; therefore, when energy production by mitochondria ceases after death, sirtuins are rendered inactive [26]. In mouse brain, HDAC 1 and 2 are the most prevalent, HDAC 3, 4, and 5 moderately so, and HDAC 6 is of low abundance [25]. In human frontal cortex, HDACs 1, 2, 5, and 6 are present, with HDAC2 the most highly expressed and HDAC 5 the least [27]. In rat hippocampus, during the first postnatal week, HDAC2 is predominantly nuclear, and then appears in neuronal cytoplasm [28]. In young adult and 12-month-old mouse cingulate cortex and hippocampus, HDACs 1, 2, and 3 are present in neurons and astrocytes; in the aging brain, they move from being primarily cytoplasmic to nuclear [29]. We postulate that increased post-mortem permeability of nuclear membranes allows cytoplasmic HDACs to enter the nucleus, creating an environment for nonspecific deacetylation of histones. Another possible explanation for the post-mortem loss of immunostaining is nonspecific block of the epitope by something liberated by autolysis.

The reason why some acetylation PTMs are more stable than others is unclear. On mammalian histone H3, acetylation is sequential with lysine 14 acetylated before lysine 23, 18, and 9 [30]. We observed that H3K14ac was most stable, particularly in glial cells. On histone H4, lysine 16 is acetylated before 8 and 12, and 5 [30]. We observed that H4K5ac had less PMD stability than H4K12ac and H4K16ac. It is possible that post-mortem deacetylation of histone PTMs occurs in the reverse order of their post-translational addition. Loss of immunostaining was observed mainly in neurons with relative preservation of all epitopes in the nuclei of glial cells. This might be explained by the amounts of the HDACs present in the different cell types. In rat brain, HDAC 2, 3, 5, and 11 expression is high in neurons, moderate in oligodendrocytes, and low in astrocytes [31–35].

Global histone methylation was generally stable. Demethylation by histone lysine demethylases (HDM) is dependent on oxygen delivery to flavin-adeninedinucleotide and therefore unlikely to occur in dead tissue [36]. HDMs tend to be specific with regard to the type of histone lysine residue they will demethylate [37]. The selectivity of HDMs might explain the relative lack of post-mortem change.

In contrast to pig and mouse brain where global H4K5ac labelling diminished after 1-day post-mortem, H4K5ac labelling persisted in the human brain samples even at 4–5 days post-mortem. This might be a chance observation in the small number of human samples. This might reflect upon the specificity of the antibodies, which were raised against human epitopes; however, core histone proteins H3 and H4 are highly conserved in mammals. Another possibility is the different agonal states between humans (the subjects died under various circumstances) and animals (which were anesthetized very briefly for euthanasia). In rodents, single brief (e.g., 20 min) or long (e.g., 2 h daily) exposures to sevoflurane or isoflurane anesthesia have been shown to promote hypermethylation of certain genes in the brain; however, this is seen at the earliest 6 h after exposure [38, 39]. A 1-h long exposure to sevoflurane decreased histone acetylation levels of the circadian rhythm gene Period2 (*Per2)* in the mouse suprachiasmatic nucleus [40]. Artifact related to tissue oxidization cannot be excluded; the human tissue array slides were subjected to immunohistochemistry < 24 h after being prepared, whereas the pig and mouse slides were cut and stored for 1–12 weeks before use [41]. Another possible explanation is the baseline level of HDACs in developing brain versus the mature brain; we studied young pigs and mice, but the human samples were derived largely from middle age individuals [42].

Although changes related to hypoxia were not a primary outcome measure in this study, because donated neonatal pig brain samples were from a well-controlled experiment, they deserve a brief comment. In hypoxic pig brain samples, ELISAs showed higher global levels of 5mC and 5hmC (Fig. 1; not significant) and Western blots showed lower levels H4K5ac, H4K12ac, H3K4me3, and H3K27me3 and slightly higher levels of H3K36me3 (Additional file 4; not significant). The two hypoxic samples in the human brain microarray showed overall decreases in the intensity of immunoreactivity for most epigenetic marks, which likely reflects cell compromise. In general, decreases in histone H3 and H4 acetylation and increases in DNA methylation have been reported in the context of experimental cerebral ischemia [10, 43], global hypoxia [44], and in cultured cells subjected to hypoxia [45–47].

The shortcomings to this study are primarily technical. We cannot fully explain why the post-mortem stability of histone PTMs in pig brain appears to differ between immunohistochemical detection and Western blotting methods. Formalin fixation arrests protein changes quickly and immunohistochemistry allows regional and subcellular evaluation of antibody binding patterns. Isolation of nuclei from frozen preceded Western blotting; the histone purification procedures might be associated with some artifactual changes despite the inclusion of protease inhibitors. The brain regions assessed also differed; frontal cerebrum was used for Western blot, and temporal/parietal cortex was used for immunostaining. Differences in the antibody incubations likely do not explain the discrepancy. We did not test all anti-histone antibodies with multiple peptides to fully evaluate blocking in immunohistochemistry and specificity in dot blot. Some antibodies targeting specific histone marks (e.g., H4K5ac, H3K27ac) showed more widespread immunoreactivity than the “total” histone H3 and H4 antibodies, the opposite of what one would expect. The discrepancy in immunoreactivity means that use of the “total histone” H3 or H4 antibodies to normalize Western blots might not be entirely reliable. However, despite these uncertainties about the specificity of some of the antibodies, and the differences between assay results, our general conclusions about post-mortem stability remain valid.

There is growing interest in epigenetic changes in the pathogenesis of human brain diseases. Ultimately, changes in specific gene loci will be responsible. Therefore, another limitation of our study is the use of global epigenetic assays. Homogenized tissue and immunostains only show averages within the brain overall and within cell nuclei respectively. One study of rat brain tissue evaluated the effects of post-mortem delay (up to 96 h) on global DNA methylation and DNA hydroxymethylation as well as gene-specific modifications in the imprinted gene loci, *h19* and *Igf2*. Neither global nor site-specific levels of 5-methylcytosine and 5-hydroxymethylcytosine were altered as a consequence of the post-mortem intervals studied [22]. To our knowledge, only one study addresses histone gene site-specific changes in human post-mortem brain [13]. ChIP was used to measure H3K4me3 and H3K27me3 at promotors for several genes (β-globulin, glutamic acid decarboxylase, and N-methyl-d-aspartate (NMDA) receptor subunit NR2B). With increasing post-mortem intervals (5 to 30 h), histone methylation levels were similar despite variable autolysis time and tissue pH [13]. As a future direction, it will be important to investigate more gene locus-specific changes at the level of histone PTMs.

## Conclusions

Epigenetic studies that utilize global assays on post-mortem brain tissue are feasible and may be helpful to understand the pathogenesis of many neurodevelopmental and neurodegenerative diseases. We recommend that the post-mortem stability of the epigenetic marks be predetermined if human autopsy brain tissue is to be studied. The apparent stability might be dependent on the method of detection, the tissue species, the brain region analyzed, the brain cell type, and the antibodies themselves. Caution is especially needed when interpreting histone acetylation results; degradation was evident in the animal tissues by < 24 h in some cases, although there appeared to be relatively greater stability in the small number of human specimens studied. Ideally, the samples of interest should have a PMD time of ≤ 48 h, but in some circumstances (e.g., where tissue samples are rare), ≤ 72 may be reasonable. In theory, affected and control samples should be matched for age, sex, and PMD. Many anti-histone PTM antibodies remain to be validated. Comparison of developing, mature, and aged subjects, and verification of the post-mortem stability of histone PTMs using gene-specific molecular techniques such as ChIPseq and DNA cytosine modifications via bisulfite conversion would also be valuable.

## Methods

### Porcine brain

Pig brains (*Sus domesticus*) (4 normoxic, 3 hypoxic; *N* = 7) were donated by investigators studying the effects of hypoxia on immature lung [48]. Ethics approval was obtained through the University of Manitoba Animal Care Committee (Protocol # 14-008/1). Briefly, female newborn (8–24 h-old) piglets were obtained from a local pathogen-free supplier. Six of the seven piglets were raised to 3 days of age before sacrifice. Piglets were bottle fed with swine milk replacer and housed in temperature-controlled isolettes. They were raised in normobaric hypoxia (10% oxygen) or in normoxia (21% oxygen) for 72 h. The hypoxic piglets were smaller (body weight mean ± SEM: normoxic 1.71 ± 0.14 kg; hypoxic 1.53 ± 0.07 kg). Animals were euthanized with an intraperitoneal injection of pentobarbital followed by exsanguination. The time between injection and pedal reflex test was approximately 5–10 min. The scalp was stripped, and the skull was opened with scissors. The medulla-spinal cord junction was transected, then the brain was removed, weighed, and immediately placed into a humid container. Concordant with body weights, brains from the hypoxic piglets were smaller (normoxic 35.11 ± 0.40 g; hypoxic 31.15 ± 0.26 g). The cerebellum and brainstem were frozen separately and used for assay optimization. The cerebral hemispheres were split in the midline, then sliced into 8 or 14 pieces of neocortex, which were each assigned a PMD time point of 0, 24, 48, or 72 h (Additional file 8: Figure S1). Therefore, each pig brain yielded all four PMD time points. The delays were designed to mimic human post-mortem and autopsy conditions, where some bodies are cooled immediately after death (e.g., death in hospital) while others are subject to a room temperature delay (e.g., found dead in home). The 0 h specimens were formalin-fixed or frozen immediately, while the other brain pieces remained in humidified Petri dishes at 4 °C until the designated time (24, 48, or 72 h). Some pieces remained at room temperature for 6–8 h before being transferred to 4 °C. One sample from each pair was immersion fixed in 10% buffered formalin (Fisher Scientific #23-245-685) and the other sample was frozen and stored at − 70 °C for biochemical assays. After fixation for no more than 1 week, the samples were painted with various colors (to designate the PMD identity) before being placed in 3 × 2 cm tissue cassettes for embedding in single blocks of paraffin. This ensured that all immunostaining conditions were identical for comparison of the different time points. To test for adverse effects of overfixation, brain samples from the 24-h PMD remained in formalin for 2.5, 5, 6, 8, 9, 10, and 12 weeks followed by sample coloring and embedding in a single block of paraffin.

### Murine brain

Ethics approval was obtained through the University of Manitoba Animal Care Committee (Protocol # 14-012; AC10924). Outbred CD1 mice (*Mus musculus*) were locally supplied in three age groups: 0–24 h-old newborn (8 male, 8 female; body weight 1.64 ± 0.03 g), 10-day (8 male, 8 female; body weight 7.02 ± 0.23 g) and 8–10 week (8 male, 8 female; body weight 36.67 ± 1.01 g). Two male and 2 female brains from each age were processed at each PMD time point of 0, 24, 48, and 72 h. Two dams (~ 13 weeks; body weight 44.12 ± 2.48 g) were sacrificed for a PMD time of 96 h. That is a total of 16 brains per age group (four sets of PMD time points; 48 mouse brains total) + two dam brains. Newborn mice were euthanized by direct decapitation with scissors (no anesthetic). Infant and adult mice were euthanized by a 4–5% isoflurane anesthesia followed by bilateral pneumothorax or cervical dislocation. The time between isoflurane exposure and pedal reflex test was approximately 10–20 min. The scalp was opened with scissors, and the brain was removed and weighed (newborns 0.085 ± 0.003 g; 10-day 0.33 ± 0.01 g; young adults 0.49 ± 0.01 g). Each mouse brain gave rise to a single PMD time point. The right hemisphere was weighed and frozen at − 70 °C for biochemical assays. The left hemisphere was fixed in 10% buffered formalin followed by paraffin embedding. The cerebellum and brainstem were discarded (Additional file 8: Figure S2). Samples for the 0 h time point were formalin-fixed or frozen immediately. For the remaining time points, brains remained at room temperature in a humidified Petri dish for 6.9 ± 0.1 h before transfer to 4 °C. Fixed brain samples were colored and processed in the same manner as pig brains.

### Human brain

A tissue array consisting of anonymized brain samples was constructed under the Diagnostic Services Manitoba institutional approval for quality assurance procedures. Neuropathology records were reviewed to identify neocortical specimens with no microscopic abnormality (determined upon examination by a neuropathologist) that had been formalin-fixed after a range of devitalization or post-mortem periods. Brain samples with known hypoxic damage were also identified. Four (4) millimeter (mm) diameter samples were punched out of 14 formalin-fixed paraffin-embedded tissue blocks and assembled into a single 5 × 3 array (Additional file 9). The tissue array paraffin block was cut at 5 μm and mounted onto slides for immunohistochemistry with antibodies to the following epigenetic modifications: 5mC, 5hmC, 5fC, 5caC, H3K4me3, H3K27me3, H3K36me3, H3panAc, H3K9ac, H3K14ac, H3K27ac, histone H4 acetylated at lysine 5 (H4K5ac), and H4K12ac (see details below). Three epigenetic modifications used in the animal studies were not done on the human samples: anti-H3K9me2, K9me3 had been discontinued by the manufacturer, a new lot of anti-H3K9me2 could not be optimized for immunostaining (selected to replace H3K9me2, K9me3, and a new lot of anti-H3K27me2 failed the peptide blocking test and was therefore not used.

### Histone extraction

Histones were extracted from the porcine brain using the Histone Extraction Kit (Abcam; #113476) according to the manufacturer’s protocol with minor changes. Briefly, 0.1 g of tissue was put into 500 μl of 1× pre-lysis buffer, homogenized, and centrifuged to obtain a pellet. Pellets were resuspended in 400–700 μl of lysis buffer, mixed, and put on ice for 1 h. The samples were centrifuged to obtain a supernatant that contained the acid extracted histones, which were then transferred into a pH neutralizing buffer solution containing dithiothreitol (DTT), followed by the addition of phenylmethylsulfonyl fluoride (PMSF) to inhibit proteases. Histone protein concentrations were measured using the Bradford method (Bio-Rad DC Protein Assay kit # 5000112). Absorbance was measured at 750 nm on an Epoch Microplate Reader (Gen5 2.08 Software), and concentrations were calculated from a standard curve. The average histone concentration was 33.8 μg/μl from the 0-h sample, 26.9 μg/μl from the 24-h sample, 22.1 μg/μl from the 48-h sample, and 19.6 μg/μl from the 72-h sample.

### DNA extraction and purification

DNA was extracted using the DNeasy Blood and Tissue DNA extraction kit (Qiagen #69505) according to the manufacturer’s protocol with minor changes. Briefly, porcine brain samples (25-30 mg) were thawed at room temperature, combined with primary lysis buffer and proteinase K, mixed, and incubated (with periodic stirring) in a water bath at 56 °C for 3–3.5 h. RNase A (100 mg/ml) was added to the samples, and after 2 min at room temperature, secondary lysis buffer and ~ 100% ethanol were added and mixed immediately. The mixture was pipetted onto the DNeasy Mini Spin column, centrifuged, and repeatedly washed. DNA captured in the DNeasy membrane was eluted with AE buffer twice (total eluted volume of 400 μl). DNA was quantified and tested for purity using spectrophotometric analysis at 260 nm and 280 nm (Ultrospec 3000), then stored at 4 °C. The average DNA concentration was 0.023 μg/μl (12.5 μg) per sample. All DNA samples were precipitated in a 1:10 volume of 3 M sodium acetate at pH 5.2 followed by addition of an equal volume of ~ 100% ice-cold ethanol and stored at − 20 °C overnight. Samples were mixed then centrifuged at 9660–15000*g* for 1 h, the DNA pellet was washed twice with ice-cold 70% ethanol and centrifuged for 30 min. DNA pellets were air dried overnight then dissolved in Qiagen Elution Buffer (Tris-EDTA solution) to the desired volume at ~ 50 °C for 10 min, mixed, and stored at 4 °C. DNA was quantified using spectrophotometric analysis at 260 nm (Ultrospec 3000; Pharmacia Biotech) to obtain final concentrations and at 280 nm to verify purity.

### DNA integrity

Twelve-well 0.8% agarose gels were prepared by combining 0.8 g of agarose (Invitrogen UltraPure Agarose, #15510-027) in 100 ml of 0.5× running buffer (1 L of DNA/RNA-free water (Gibco, #10977-015) with 50 mL of 10X Tris-borate-EDTA (TBE) buffer). Gel Star Nucleic Acid Gel Stain (10 μl, Lonza #50535) was added before the gel was set. Cold 6X DNA loading buffer (2.5% Ficoll-400, 11 mM EDTA, 3.3 mM Tris-HCl, 0.017% sodium dodecyl sulfate (SDS), 0.015% bromophenol blue, pH 8.0 25 °C) and DNA samples were combined in a 3:1 ratio of loading buffer to DNA, then pipetted into individual wells (20 μl/well) along with a DNA ladder (Quick-Load DNA Marker, Broad Range; NEB #N0303). The gels ran at 100 V for 1.5 h and were imaged using a chemiluminescence imager and computer program (FluorChem HD2; ProteinSimple, San Jose, CA).

### Western blotting

Histone samples (30 μg/8 μl per well; in duplicate) were prepared in a 1:1 ratio with 2× sample buffer (1 M Tris pH 6.8, 35% glycerol, 10% SDS, 0.2 mg Bromophenol Blue), loaded into a 15-lane SDS–15% polyacrylamide gel, and separated by electrophoresis (135 V, 100 min) with 1× running buffer at pH 8.3 (25 mM Tris, 182 mM Glycine, 0.1% SDS) along with protein standard (Bio-Rad, #161-0374) and a positive control (1 mg/μl of pure histone mix from calf thymus; Roche, #10 223 565 001). Samples were transferred onto 0.2 μm polyvinylidene difluoride (PVDF) membrane (Bio-Rad, #162-0177) at a constant amperage of 400 mA (70 min on ice) using a wet transfer system and a transfer buffer with no SDS (25 mM Tris, 192 mM glycine, pH 8.3, 20% methanol). Protein transfer was verified with Ponceau S stain. The PVDF membrane was incubated with 5% skim-milk (Carnation Fat Free Instant Skim Milk powder) blocking solution or bovine serum albumin (BSA) (Sigma, A9647; Chem Cruz, SC-2323) in 1× TBS (depending on the antibody) for 1.5 h at room temperature. Membranes were cut in half; one half was incubated with primary antibodies specific for histone PTMs and the other half with the respective “total histone” antibody (either H3 or H4) overnight (Additional file 10 and Additional file 11: Table S1). Peroxidase-conjugated AffiniPure sheep anti-mouse IgG (Jackson ImmunoResearch #515-035-062) or goat anti-rabbit IgG (Sigma, A6154) was applied to the membrane at a concentration of 1:7000 for 2 h at room temperature. Chemiluminescent reagent (Clarity Western ECL Substrate, Bio-Rad #170-5060) applied to the membrane for 3 min; histone bands were visualized on Classic Blue Autoradiography Film BX (MidSci, EBNU2).

Bands were quantified using the Bio-Rad ChemiDoc TM MP System (Universal hood III model) and ImageLab Software (Version 5.2.1). The software generated a table with, “relative front” (position related to molecular size), the “volume” of the band (a product of the band area and the mean intensity of all pixels within the band area boundaries), and the relative quantification (in comparison to the reference band, which is the 0-h time point). All bands underwent automatic background subtraction (“rolling ball” method; disk size 10 mm). The specific histone modification “volume” was divided by the respective total histone “volume” and all results are shown as the normalized “volume” ratios.

### DNA methylation enzyme-linked immunosorbent assay

The 5mC DNA methylation enzyme-linked immunosorbent assay (ELISA) Kit (Zymo, #D5325) kit was used to measure DNA methylation in pig brain samples using the indirect method. The manufacturer’s suggested protocol was followed, except for the addition of gentle rocking/agitation during all 37 °C incubation steps. Briefly, 100 ng of purified DNA from each pig brain sample (*N* = 7) was brought to a total volume of 200 μl with 5mC coating buffer. Samples, along with the standards, were heated at 98 °C then immediately iced to denature the DNA. Samples (100 μl) were pipetted in triplicate into the 96-well 5mC coated plate and incubated at 37 °C for 1 h. The residual fluid was discarded and the wells were repeatedly washed, then 200 μl of 5mC ELISA blocking buffer was added and incubated at 37 °C for 30 min. The buffer was discarded and 100 μl of anti-5-methylcytosine (1:2000) and secondary antibody (1:1000) were simultaneously added to the wells and incubated at 37 °C for 1 h. The wells were washed, then 100 μl of horseradish peroxidase (HRP) developer was added and absorbance was measured every 5–10 min at 405 nm using the Epoch Microplate Reader (Gen5 2.08 Software) to measure the color change (yellow/green to bluish/green). Absorbance values were averaged and the percentage of 5-methylcytosine was calculated (%5mC = *e*{((absorbance − *y*-intercept)/slope)}) and multiplied by the fold difference in CpG density of the *E. coli* derived standards (*E. coli*/pig 0.0747/0.0108 = 6.91). Values are reported as the percent of CpG methylation. Originally, we tried the Global DNA Methylation ELISA Kit (5′-methyl-2′-deoxycytidine Quantitation) (Cell Biolabs, #STA-380). However, our calculated sample values fell below the linear curve, which precluded proper calculation of global DNA methylation.

### DNA hydroxymethylation enzyme-linked immunosorbent assay

The Quest 5-hmC DNA ELISA Kit (Zymo, #D5426) kits was used to measure DNA hydroxymethylation levels in the pig brain samples using the sandwich method. The manufacturer’s suggested protocol was followed, except for the addition of gentle rocking/agitation during all 37 °C incubation steps. Briefly, plate wells were loaded with 1 ng/μl of anti-5-hydroxymethylcytosine polyclonal antibody diluted in coating buffer and incubated at 37 °C for 1 h, then the wells were washed. Each well was filled with 200 μl of 5mC ELISA buffer, then the plate was incubated at 37 °C for 30 min. Control DNA (1 ng) and purified sample DNA (1 ng) in triplicate from each pig brain sample was heated at 98 °C for 5 min, cooled on ice, then pipetted into each well and incubated at 37 °C for 1 h. The wells were washed, 100 μl of anti-DNA antibody conjugated to HRP (1:100) was added and incubated at 37 °C for 30 min, the wells were washed again, then 100 μl of HRP developer was added, and absorbance was measured every 5–10 min at 405 nm using the Epoch Microplate Reader (Gen5 2.08 Software) to monitor the color change. Absorbance values were averaged, and the percentage of 5-hydroxymethylcytosine was calculated using the formula provided (%5-hmC = (absorbance − *y*-intercept)/slope). Values are reported as the percent of 5-hydroxymethylcytosine in reference to the control DNA (standards).

### Immunohistochemistry

Paraffin-embedded brain samples from mice (anterior left hemisphere) and pigs (temporal and parietal cortices) were sectioned at a thickness of 5-6 μm and mounted onto charged glass slides (Fisher Superfrost Plus #12-550-15, 25 X 75 X 1.0 mm) then left to dry overnight. Sections were subjected to immunohistochemistry at room temperature unless otherwise stated. Optimal primary antibody concentration was determined by running a series of dilutions greater and less than the manufacturers’ suggestions. See Additional file 10 for antibody details and Additional file 11: Table S2 for specific immunostaining conditions. Paraffin was melted at ~ 60 °C for 25 min, and tissue samples were rehydrated through graded xylene and ethanol solutions. For antigen retrieval, slides were submerged in boiling buffer in a pressure cooker (Hamilton Beach, 37539C) for 20 min, then cooled in ddH_2_0. Endogenous peroxidase was blocked with a 1:10 solution of 30% hydrogen peroxide (Fisher, H325-500) in methanol (Fisher, A433P-4) for 15 min. For the DNA cytosine modification antibodies, the tissue was incubated with 3 N hydrochloric acid (HCl) (Fisher, #351278-212) for 15 min. The slides were then subjected to a 30-min block with 10% goat (Jackson ImmunoResearch #005-000-121) or sheep (Jackson ImmunoResearch #013-000-121) serum (depending on the secondary antibody) in diluent (1% BSA in either 1× TBS or 1× PBS). Blocking solution was discarded, and the primary antibody was applied for 2 h, followed by washing (3× washing buffer #1, 1× buffer #2), application of the secondary antibody for 1 h (1:300 dilution; biotin-conjugated streptavidin goat anti-rabbit, Jackson ImmunoResearch #111-065-144 or biotin-conjugated streptavidin sheep anti-mouse, Jackson ImmunoResearch #515-065-003), followed by washing, application of peroxidase-conjugated streptavidin for 45 min (1:300 dilution; Jackson ImmunoResearch #016-030-084), washing, and finally 1:10 dilution of 30% hydrogen peroxide with 3,3′ diaminobenzidine (DAB; Sigma, D5905) to develop the slides. The slides were counterstained with Harris hematoxylin solution, dehydrated through graded ethanol and xylene solutions, coverslipped with Permount (Fisher, SP15- 100 Toluene solution VN1294), and left to air dry overnight.

### Specificity of epigenetic mark antibodies

Selected anti-histone antibodies were tested for specificity using peptide interference to block all possible methyl modifications (e.g., for H3K4me3, peptides for H3K4me3, H3K4me2, and H3K4me were used) and one acetyl modification (H3K27ac). Antibodies were prepared at the dilution used for immunostaining (Additional file 11: Table S2). Peptide solution of an equal concentration (in μg/μL) was added. Because the IgG antibodies are ~ 150 kD and the peptides are ~ 15 kD (~ 100 amino acids), this allowed for a 10:1 M concentration surplus of blocking peptide. The antibody-peptide solution was rocked gently for 30 min before being applied to the slides. The immunohistochemistry protocol was executed as described above.

Antibodies against H3K14ac, H3K27ac, H4K5ac, H4K12ac, H3K4me3, H3K27me3, H3K36me3, H3panAc, total histone H3, and total histone H4 were also tested via dot blot on nitrocellulose membrane (Bio-Rad #162-0146, Lot 9396). Peptides specific to the antibodies, peptides that might cross-react, as well as unmodified histone H3 and unmodified histone H4 were pipetted directly onto the nitrocellulose membrane (0.1, 0.5,1, or 2 μg). The membrane was air dried for 5 min, then wrapped in foil and baked for 15 min at 50–60 °C before following the same detection protocol used for Western blotting, starting at the blocking step. For the complete list of all peptides used, see Additional file 12.

### Immunohistochemical imaging and semiquantitative evaluation

Immunostained slides were imaged using a standard upright microscope (Olympus BX51TRF microscope; QImaging camera 32-0110A-568; MicroPublisher 5.0, Model LH100HG). Images were obtained at × 40, × 200, or × 400 using QCapture (v2.8.1, 2001–2005, QImaging Corp.) software. Two semiquantitative scales were developed to estimate the proportion of immunoreactive nuclei (graded 4 to 0) (Additional file 13: Figure S1) and the intensity of immunoreactivity (graded 3 to 0) (Additional file 13: Figure S2). Morphologic features of nuclei were used as a surrogate for probable brain cell type (Additional file 13: Figure S3). This is based upon the experience of the senior author (MRD), an experienced neuropathologist. The semiquantitative scale of proportion was validated by manual cell counting on photomicrographs produced at × 200 magnification. The number of positive (any degree of brown) versus negative (blue stained) nuclei were counted in two morphologic criteria: large round nuclei (neurons) and smaller round to irregular nuclei (glial cells). The long narrow nuclei of endothelial and smooth muscle cells were excluded; we observed even at the zero time point that there was an inexplicable mix of positive and negative cells using almost all antibodies.

Quantitative analysis was applied to pig brain images (all PMD) for all six histone acetylation marks, 5mC, and H3K27me3. Nuclei with any detectable brown coloration were considered positive for determination of the proportion quantitation. Using Image J 1.52a software (NIH, USA), photomicrographs created at × 400 magnification were converted to gray scale, the perimeters of large nuclei were traced, and the mean intensity was calculated. Results were graphed and compared to the semiquantitative intensity scale results. Note that the immunohistochemistry results did not have a complete 256 gray scale range because the hematoxylin has some color. Both the numeric and intensity results were found to be similar by quantitative and semiquantitative analyses. The latter approach is much quicker and therefore suited for screening of major changes, but might miss subtle differences.

### Statistical analysis

Statistical data were analyzed using JMP 13 software (SAS Institute Inc.; Cary NC), IBM SPSS Statistics software (Version 25), and the Data Analysis Tab in Microsoft Excel (Office Professional Plus 2016). Descriptive statistics were generated using Microsoft Excel to produce 95% confidence intervals. Western blot densitometry data and semiquantitative (proportion and intensity) immunohistochemistry rank assessments are non-parametric and therefore were subjected to a Wilcoxon/Kruskal-Wallis rank sums test (*p* < 0.05). The *p* values were adjusted using the Benjamini-Hochberg procedure using the formula ((i/m)*Q) and a false discovery rate (FDR) of 0.1. For mouse brain data (immunohistochemistry), all ages were pooled. ELISA data and immunohistochemistry quantitative count data were subjected to analysis of variance (ANOVA) with the Dunnett’s post hoc multiple comparisons test where *p* < 0.05 was considered significant.

## Additional files

Additional file 1: DNA integrity in neonatal pig (normoxic) frontal cortex after post-mortem delays to freezing. Agarose gel showing DNA extracted from brain samples obtained from a single pig and frozen at post-mortem delays (PMD) of 0, 24, 48 and 72 hours (hr). M represents the DNA size markers in base pairs (bp). Bands at all post-mortem delay time points remain intact with no smearing. (PDF 39 kb)

Additional file 2: **Figure S1.** Examples of peptide blocking immunohistochemistry for histone post-translational modifications in neonatal pig neocortex. Top row photomicrographs show anti-histone H3 trimethylated at lysine 4 (H3K4me3). H3K4me3 was blocked by its own peptide (K4me3), but also partially blocked by other less specific peptides (K4me, K4me2, K9me3). Bottom row photomicrographs show anti-H3K27ac. It was blocked only by its own peptide (K27ac) and not by others (H4K5ac; others not shown). Images all taken at × 400 magnification. DAB detection of antibody (brown) and hematoxylin counterstain (blue). **Figure S2.** Complete dot blot peptide results. A small quantity of peptide was directly pipetted onto nitrocellulose membrane. The membrane was blocked and incubated with the antibody overnight. (PDF 4535 kb)

Additional file 3: Total histone Western blot results. Raw densitometric quantity (“volume”) measurements are shown for all pigs combined (n=7; mean ± 95% confidence intervals). Total histone H3 and H4 levels were stable across all post-mortem delay time points (not significant at *p*<0.05). (PDF 33 kb)

Additional file 4: **Figure S1.** Western blots for each pig set of normoxic (Pigs 1-4) and hypoxic (Pigs 5-7) pigs for H3K27me3 (left) and corresponding Total Histone H3 (right). **Figure S2.** Western blots for each pig set of normoxic (Pigs 1-4) and hypoxic (Pigs 5-7) pigs for H4K12ac (left) and corresponding Total Histone H4 (right). **Figure S3.** Western blots for Pig #7 (Hypoxic) for all histone PTMs (left) and corresponding Total Histone (right). **Figure S4.** Full western blot images for H3K36me3 and H4K12ac. Histone PTM (left) corresponding Total Histone (right). L represents the protein standard ladder (kd), B represents a “blank” lane, +C represents the positive control (calf thymus histone mix). (PDF 4534 kb)

Additional file 5: **Figure S1.** Immunohistochemical detection of H3K27me2 in 10-day mouse and neonatal pig neocortex. In control brains (0 hour), the nuclei of all cells, except for a subpopulation of endothelial cells, are positive. Photomicrographs show decreased intensity of immunoreactivity in medium-size nuclei of mouse brain at 48 and 72 hours post-mortem, and in large neuronal nuclei of pig brain by 72 hours post-mortem. Images taken at × 400 magnification. DAB detection of antibody (brown) and hematoxylin counterstain (blue). **Figure S2.** Immunohistochemical detection of “total” histone H3 and H4 in neonatal pig and mouse neocortex. In control (0 hour) pig brain and 10-day and 10-week mouse brains, anti-H3 and anti-H4 label nuclei in all but a subset of endothelial cells. However, in newborn mouse brain, anti-H3 does not label the most immature cells. Photomicrographs show a minor decrease in the intensity of immunostaining of large neurons in pig brain at 72 hours post-mortem. In mouse brain at all ages, loss of H3 and H4 immunoreactivity is more substantial at 72 hours post-mortem. Images taken at × 400 magnification. DAB detection of antibody (brown) and hematoxylin counterstain (blue). (PDF 4534 kb)

Additional file 6: **Figure S1.** Bar graphs showing the semiquantitative intensity scores (mean ± 95% confidence intervals; maximum 3) for all epigenetic modification antibodies used in mouse brain. There values are shown for dentate gyrus, where there were no age-related differences (all ages combined). DNA cytosine modifications, total histone H4, all histone methylation PTMs, and H3K14ac showed stable immunoreactivity up to 72 hours post-mortem. H4K5ac showed a downward trend (not statistically significant) with increasing PMD. Other acetylation marks (H3K9ac, H3K27ac, H4K12ac and H4K16ac) tended to decrease by 24 hours and were significantly decreased by 48 hours post-mortem. *p* values for all statistical comparisons shown at bottom. A *p* value <0.05 that is not bolded in red did not pass the Benjamini-Hochberg correction. **Figure S2.** Bar graphs showing the semiquantitative proportion scores (mean ± 95% confidence intervals; maximum 4) for all epigenetic modifications in mouse brain dentate gyrus (all ages combined). DNA cytosine modifications and histone methylation were stable from 0 to 72 hours post-mortem. Total histone H3 and H4 labeling declined gradually after 48 hours. All of the acetylation modifications showed progressive declines by 24 hours, although not all of the trends were statistically significant. *p* values for all statistical comparisons shown at bottom. A *p* value <0.05 that is not bolded in red did not pass the Benjamini-Hochberg correction. (PDF 4534 kb)

Additional file 7: Post-mortem stability of brain antigens commonly evaluated in neuropathology. Method - Paraffin blocks of neonatal pig (one each raised in normoxic and mild hypoxic environment) cerebrum samples that were fixed with formalin after storage at 4˚C for 0, 24, 48, or 72 hours and then paraffin embedded were sectioned at 5μm thickness. They were subjected to immunostaining using and automated immunostaining system (Dako Envision) as done routinely for human autopsy specimens under conditions that had been optimized for use with human material. All slides were examined by a neuropathologist with extensive experience evaluating human and other mammalian brains. Results - There are no differences comparing normoxic to hypoxic newborn pig brains. Several antigens had no change in detection intensity, but the pattern was smudged or globular (+/- increased background) after a 72 hour delay to fixation. This suggests degradation of the cell and / or antigen leaking from cells. (PDF 4534 kb)

Additional file 8: **Figure S1.** Pig brain dissection. Lines on the photograph show the dissection planes used to divide the pig brain into samples used for formalin fixation and subsequent immunohistochemical staining (white) or freezing at -70˚C and subsequent biochemical analyses (yellow). **Figure S2.** Mouse brain dissection. The left brain hemisphere was used for formalin fixation (FFPE) and subsequent immunohistochemical staining and the right hemisphere for freezing at -70˚C. (PDF 4534 kb)

Additional file 9: Human brain tissue microarray - case details and construction. (PDF 97 kb)

Additional file 10: Epigenetic mark antibody details and sources. (PDF 4535 kb)

Additional file 11: **Table S1.** Antibody dilutions and respective Western Blotting conditions. **Table S2.** Antibody dilutions and respective Immunohistochemistry conditions. (PDF 4535 kb)

Additional file 12: Peptides used for antibody specificity experiments. (PDF 39 kb)

Additional file 13: **Figure S1.** Standard scale images used for grading the proportion of immunoreactive nuclei. Any degree of brown was considered positive. Photomicrographs show: 4 - ~100% positive; 3 - ~75% positive; 2 - ~50% positive; 1 <25% positive; 0 - ~0% positive. Images taken at × 400 magnification. DAB detection of antibody (brown) and hematoxylin counterstain (blue). **Figure S2.** Standard scale images used for grading the intensity of immunoreactive nuclei. Strong labeling (3) was almost black with no nuclear details visible (arrows). Medium labeling (2) is distinctly brown but with chromatin stippling or nucleolus showing (arrows). Faint labeling (1) has brown coloration (arrows) that only barely obscures the blue staining in negative (0) cells (arrowheads). For grading, the majority level was the score assigned. Images taken at × 400 magnification. DAB detection of antibody (brown) and hematoxylin counterstain (blue). **Figure S3.** Cell type (probable) based on nuclear morphology. (PDF 4533 kb)

## Abbreviations

5caC: 5-Carboxylcytosine
5fC: 5-Formylcytosine
5hmC: 5-Hydroxymethylcytosine
5mC: 5-Methylcytosine
ANOVA: Analysis of variance
ChIP: Chromatin immunoprecipitation
CpG: Cytosine-phosphate-guanine
DAB: 3,3′ Diaminobenzidine
DNA: Deoxyribonucleic acid
DNMT: DNA methyltransferase
DTT: Dithiothreitol
EDTA: Ethylenediaminetetraacetic acid
ELISA: Enzyme-linked immunosorbent assay
FDR: False discovery rate
H3panAc: Pan-acetyl histone H3
HCl: Hydrochloric acid
HDAC: Histone deacetylase
HDM: Histone demethylase
HRP: Horseradish peroxidase
IHC: Immunohistochemistry
PCR: Polymerase chain reaction
PMD: Post-mortem delay
PMSF: Phenylmethylsulfonyl fluoride
PTM: Post-translational modification
PVDF: Polyvinylidene difluoride
RNA: Ribonucleic acid
SDS: Sodium dodecyl sulfate
SEM: Standard error of the mean
TET: Ten-eleven translocation methylcytosine dioxygenase
