# Supplementary material for: DNA methylation and histone post-translational modification stability in post-mortem brain tissue
Source: Clin Epigenetics. 2019 Jan 11;11:5. doi: 10.1186/s13148-018-0596-7 (PMC6330433; doi:10.1186/s13148-018-0596-7)

Additional File 1

DNA integrity in neonatal pig (normoxic) frontal cortex after post-mortem delays to freezing. Agarose gel showing DNA extracted from brain samples obtained from a single pig and frozen at post-mortem delays (PMD) of 0, 24, 48 and 72 hours (hr). M represents the DNA size markers in base pairs (bp). Bands at all post-mortem delay time points remain intact with no smearing.

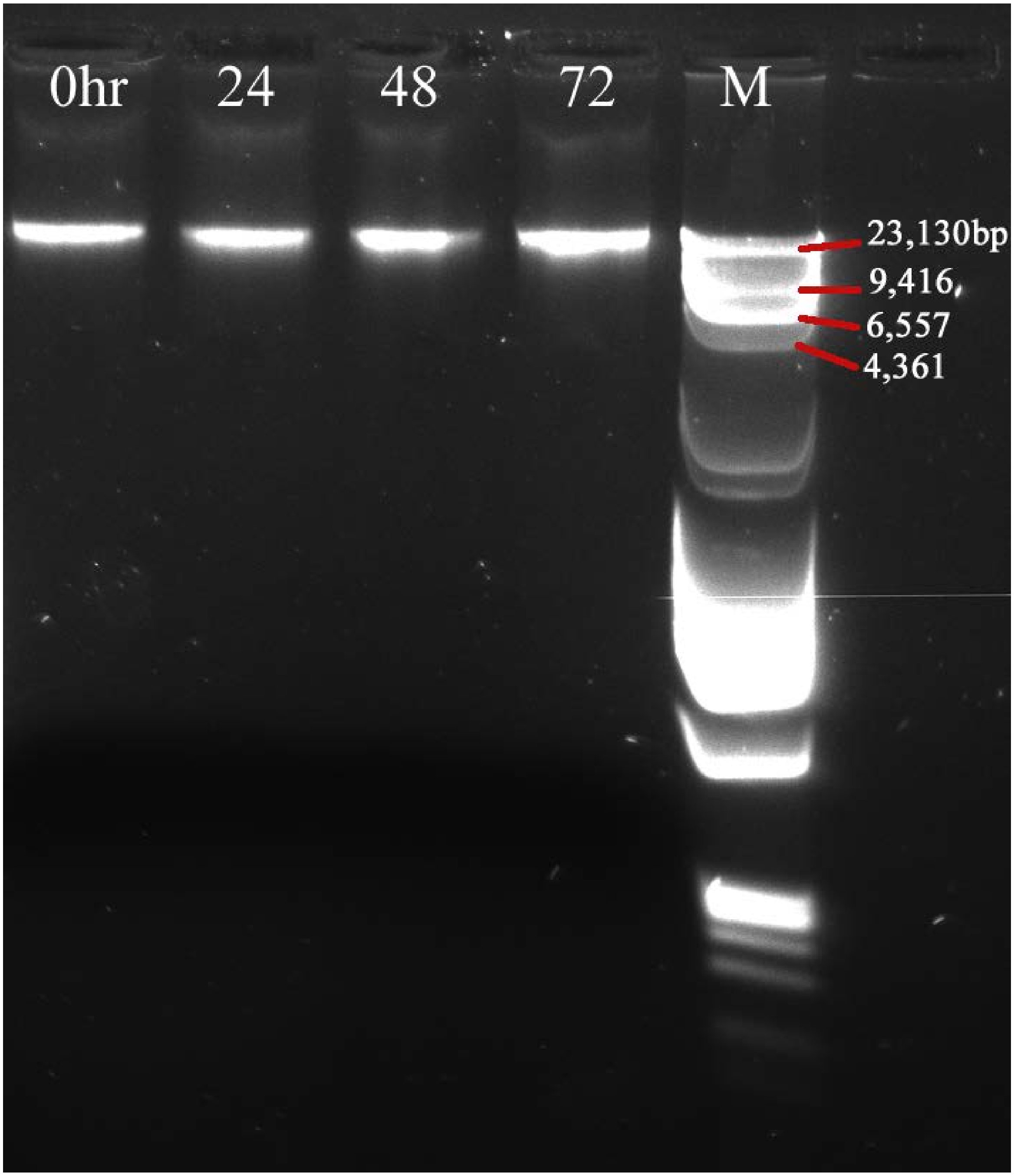

Supplement: Supplementary file 1 — DNA integrity in neonatal pig (normoxic) frontal cortex after post-mortem delays to freezing. Agarose gel showing DNA extracted from brain samples obtained from a single pig and frozen at post-mortem delays (PMD) of 0, 24, 48 and 72 hours (hr). M represents the DNA size markers in base pairs (bp). Bands at all post-mortem delay time points remain intact with no smearing. (PDF 39 kb) [file 13148_2018_596_MOESM1_ESM.pdf]
