# Supplementary material for: DNA methylation and histone post-translational modification stability in post-mortem brain tissue
Source: Clin Epigenetics. 2019 Jan 11;11:5. doi: 10.1186/s13148-018-0596-7 (PMC6330433; doi:10.1186/s13148-018-0596-7)
Supplement: Supplementary file 2 — Figure S1. Examples of peptide blocking immunohistochemistry for histone post-translational modifications in neonatal pig neocortex. Top row photomicrographs show anti-histone H3 trimethylated at lysine 4 (H3K4me3). H3K4me3 was blocked by its own peptide (K4me3), but also partially blocked by other less specific peptides (K4me, K4me2, K9me3). Bottom row photomicrographs show anti-H3K27ac. It was blocked only by its own peptide (K27ac) and not by others (H4K5ac; others not shown). Images all taken at × 400 magnification. DAB detection of antibody (brown) and hematoxylin counterstain (blue). Figure S2. Complete dot blot peptide results. A small quantity of peptide was directly pipetted onto nitrocellulose membrane. The membrane was blocked and incubated with the antibody overnight. (PDF 4535 kb) [file 13148_2018_596_MOESM2_ESM.pdf]

Additional File 2

Figure S1: Examples of peptide blocking immunohistochemistry for histone post-translational modifications in neonatal pig neocortex. Top row photomicrographs show anti-histone H3 trimethylated at lysine 4 (H3K4me3). H3K4me3 was blocked by its own peptide (K4me3), but also partially blocked by other less specific peptides (K4me, K4me2, K9me3). Bottom row photomicrographs show anti-H3K27ac. It was blocked only by its own peptide (K27ac) and not by others (H4K5ac; others not shown). Images all taken at x 400 magnification. DAB detection of antibody (brown) and hematoxylin counterstain (blue).

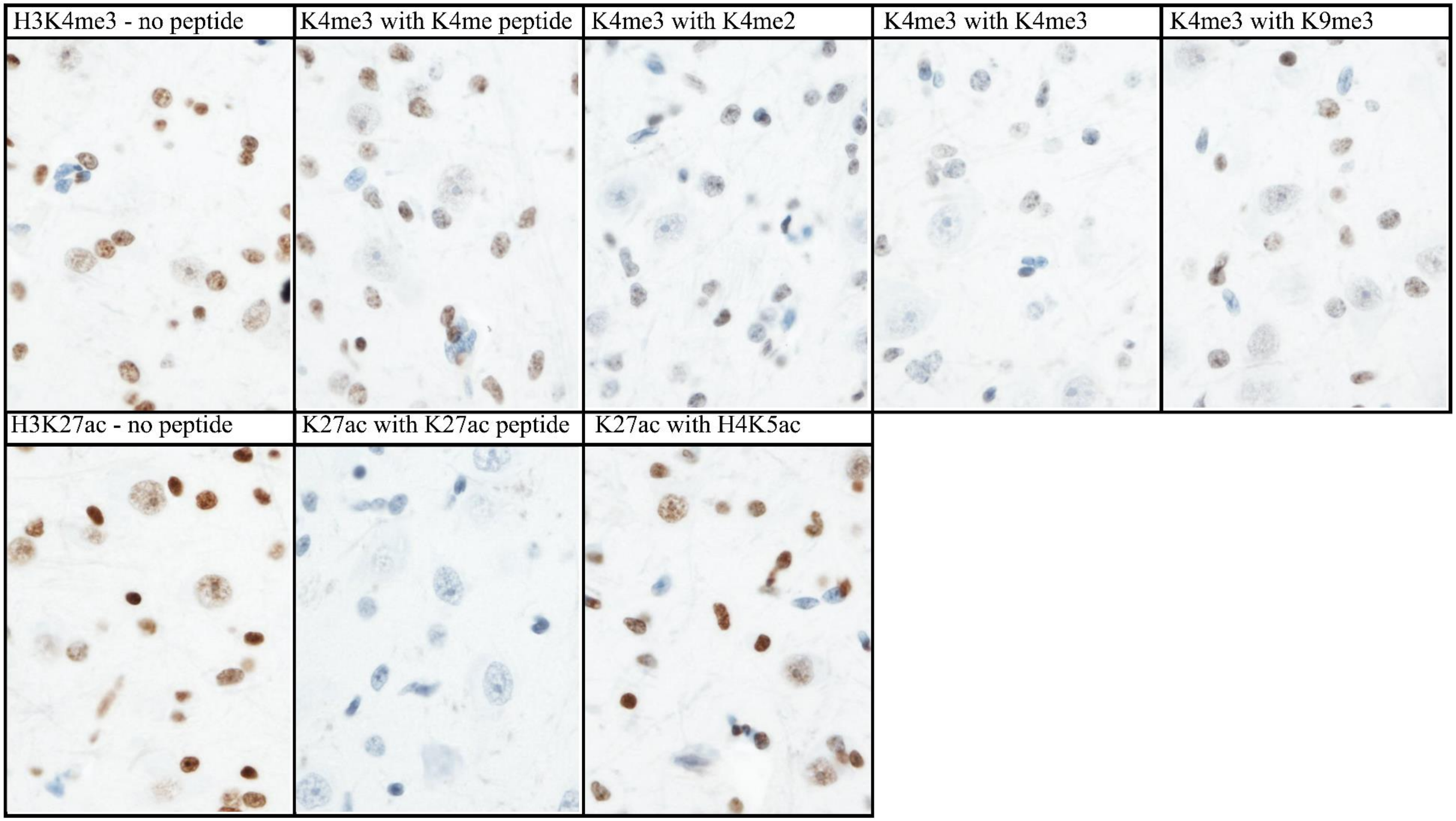

Figure S2: Complete dot blot peptide results. A small quantity of peptide was directly pipetted onto nitrocellulose membrane. The membrane was blocked and incubated with the antibody overnight.

Unmodified H3 **0.5µg**	H3K27ac **0.5µg**	H3K9me **0.5µg**
Unmodified H3 **2µg**	H3K27ac **2µg**	H3K9me **2µg**
Unmodified H4 **0.5µg**	H4K5ac **0.5µg**	H3K27me3 **0.5µg**
Unmodified H4 **2µg**	H4K5ac **2µg**	H3K27me3 **2µg**

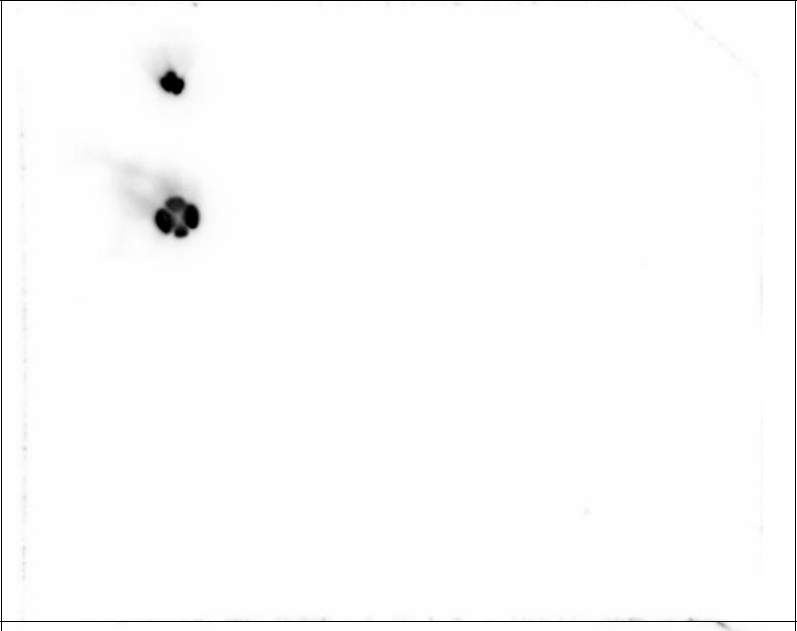

Total Histone H3

Unmodified H4 **0.5µg**	H4K5ac **0.5µg**	H4K12ac **0.5µg**
Unmodified H4 **2µg**	H4K5ac **2µg**	H4K12ac **2µg**
Unmodified H3 **0.5µg**	H3K27ac **0.5µg**	H3K9me2 **0.5µg**
Unmodified H3 **2µg**	H3K27ac **2µg**	H3K9me2 **2µg**

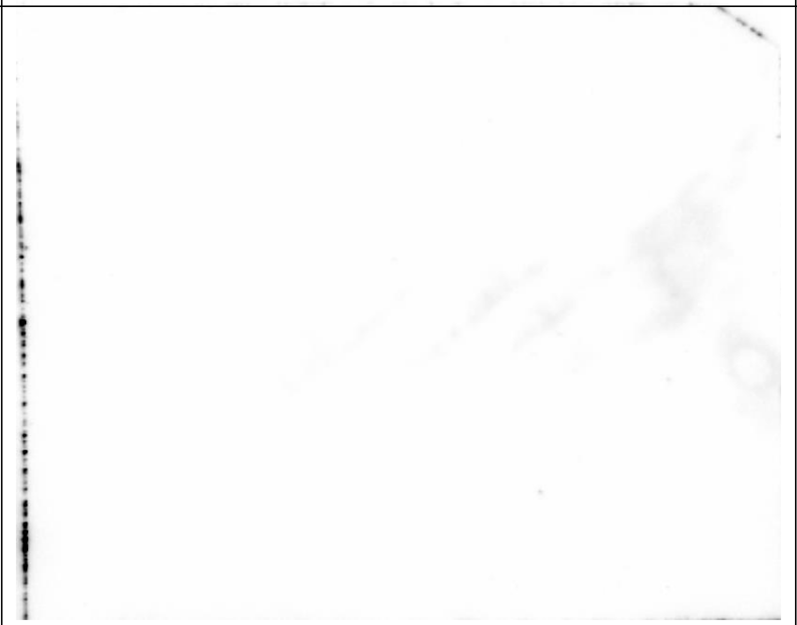

Total Histone H4

Unmodified H4 H3K14ac H3K27ac<br>**0.5µg 0.5µg 0.5µg**<br>Unmodified H4 H3K14ac H3K27ac<br>**2µg 2µg 2µg**<br>H4K5ac --><br>**0.1µg 0.5µg 2µg**<br>H4K12ac --><br>**0.1µg 0.5µg 2µg**	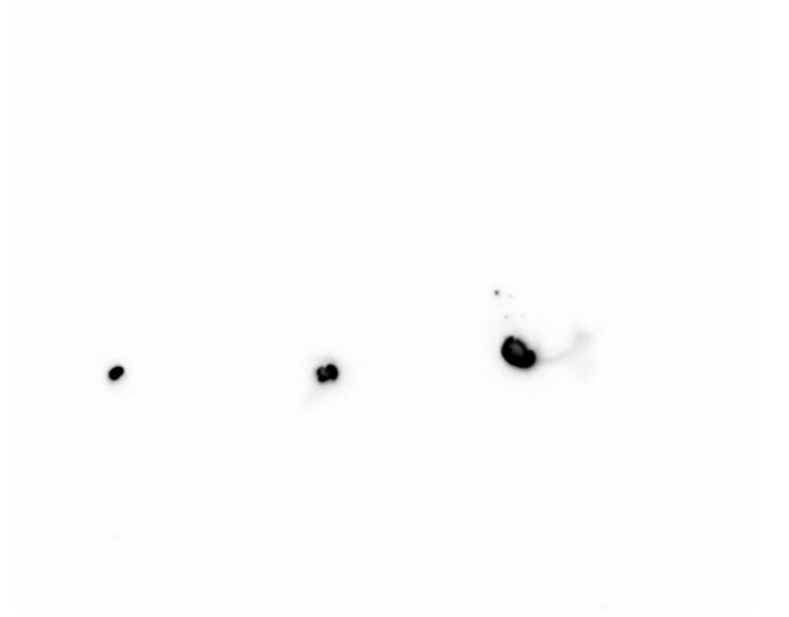	H4K5ac
Unmodified H4 H3K14ac H3K27ac<br>**0.5µg 0.5µg 0.5µg**<br>Unmodified H4 H3K14ac H3K27ac<br>**2µg 2µg 2µg**<br>H4K5ac --><br>**0.1µg 0.5µg 2µg**<br>H4K12ac --><br>**0.1µg 0.5µg 2µg**	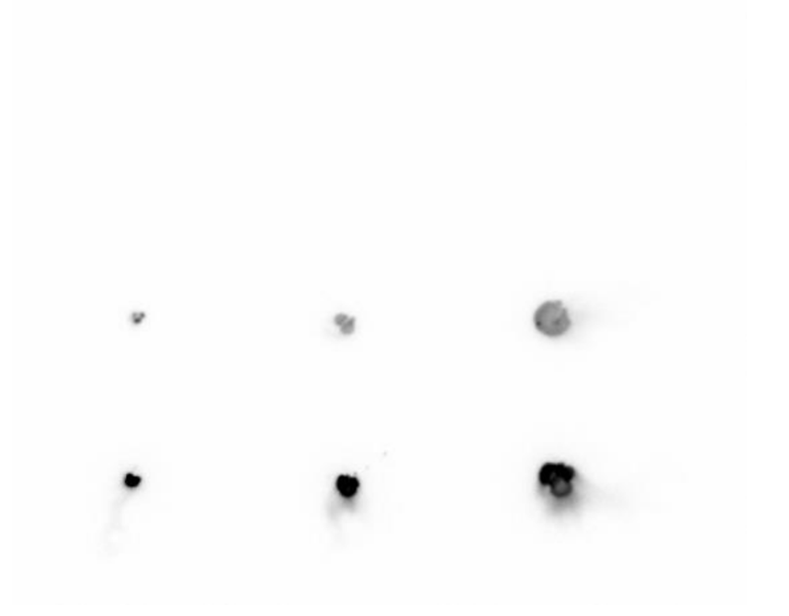	H4K12ac

Unmodified H3 **0.5µg** | H4K5ac **0.5µg** | H4K12ac **0.5µg**

Unmodified H3 **2µg** | H4K5ac **2µg** | H4K12ac **2µg**

H3K14ac -->
0.1µg **0.5µg** **2µg**

H3K27ac -->
0.1µg **0.5µg** **2µg**

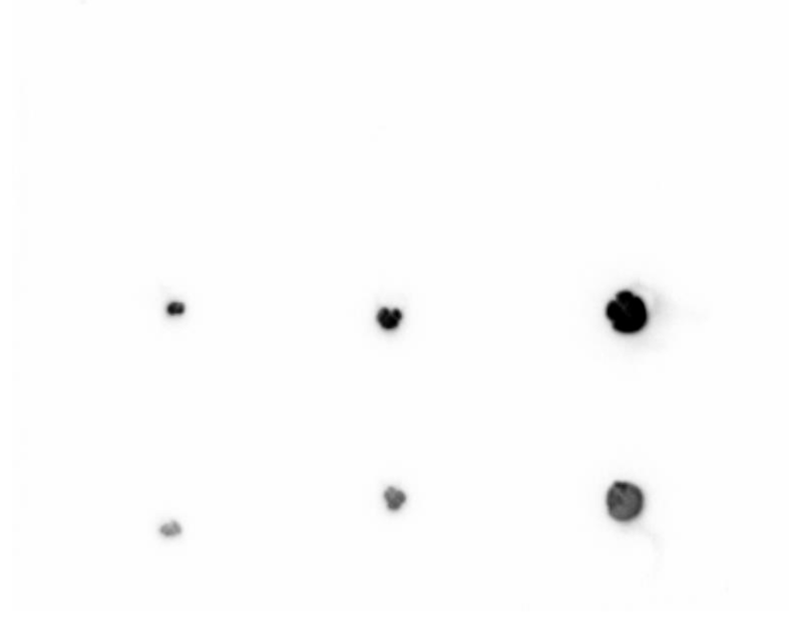

H3panAc

H3K4me--->
0.1µg **0.5µg** **2µg**

Unmodified H3
0.5µg

H3K4me2--->
0.1µg **0.5µg** **2µg**

Unmodified H3
2µg

H3K4me3--->
0.1µg **0.5µg** **2µg**

H3K27me3
2µg

H3K9me3 **0.5µg** | H3K9me3 **2µg** | H3K27me3 **0.5µg**

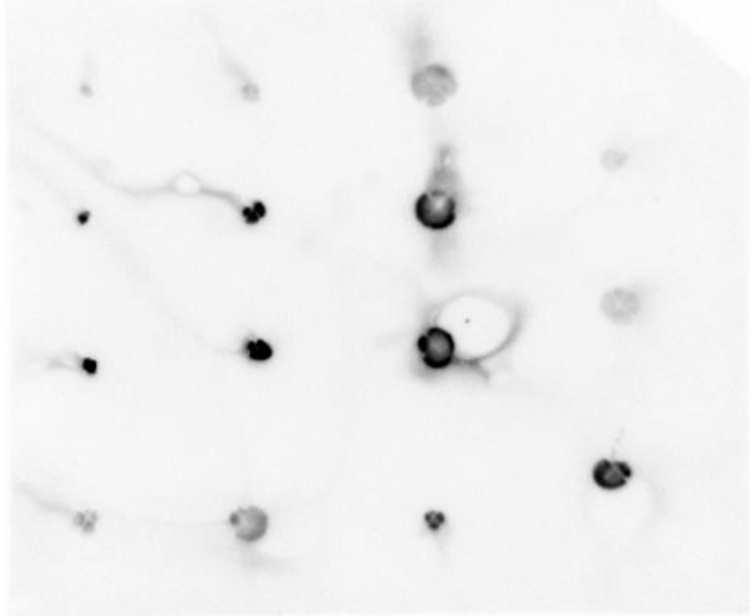

H3K4me3 (Active Motif)

H3K27me--><br>**0.1µg** **0.5µg** **2µg**<br>Unmodified H3<br>**0.5µg**<br>H3K27me2--><br>**0.1µg** **0.5µg** **2µg**<br>Unmodified H3<br>**2µg**<br>H3K27me3--><br>**0.1µg** **0.5µg** **2µg**<br>H3K36me3<br>**2µg**<br>H3K9me3 H3K9me3 H3K36me3<br>**0.5µg** **2µg** **0.5µg**	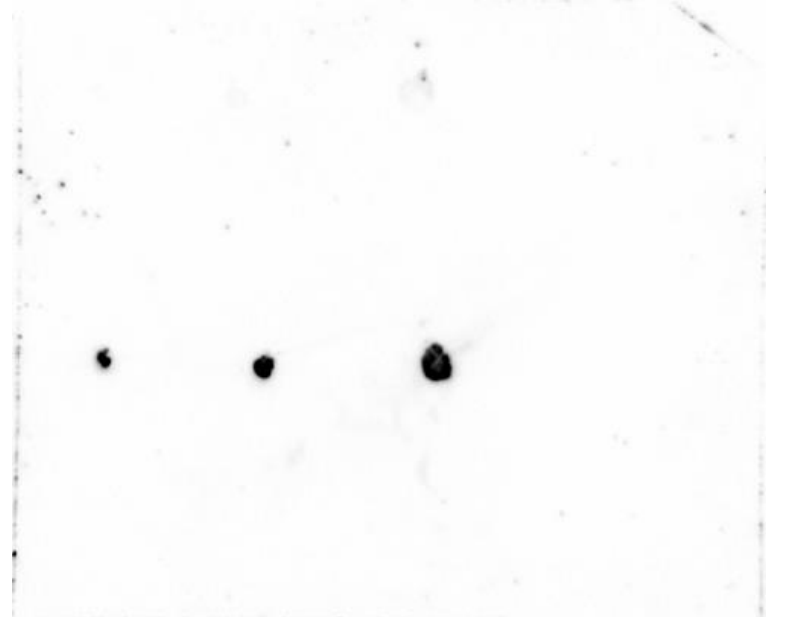	H3K27me3
H3K36me--><br>**0.1µg** **0.5µg** **2µg**<br>Unmodified H3<br>**0.5µg**<br>H3K36me2--><br>**0.1µg** **0.5µg** **2µg**<br>Unmodified H3<br>**2µg**<br>H3K36me3--><br>**0.1µg** **0.5µg** **2µg**<br>H3K27me3<br>**2µg**<br>H3K4me3 H3K4me3 H3K27me3<br>**0.5µg** **2µg** **0.5µg**	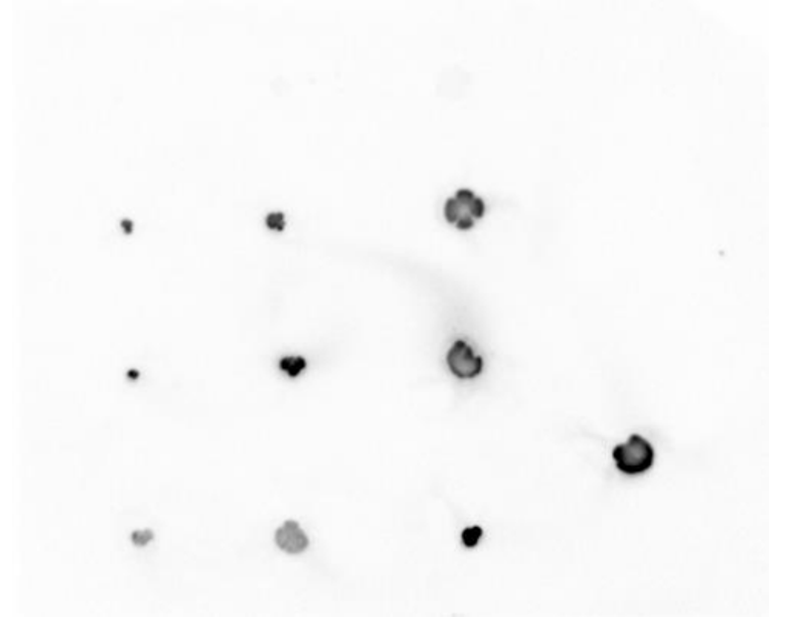	H3K36me3

Histone Mix --><br>**0.1µg** **0.5µg** **2µg**<br><br>Unmodified H4 --><br>**0.1µg** **0.5µg** **2µg**<br><br>H4K5ac H4K12ac Unmodified H3<br>**0.5µg** **0.5µg** **0.5µg**<br><br>H4K5ac H4K12ac Unmodified H3<br>**2µg** **2µg** **2µg**	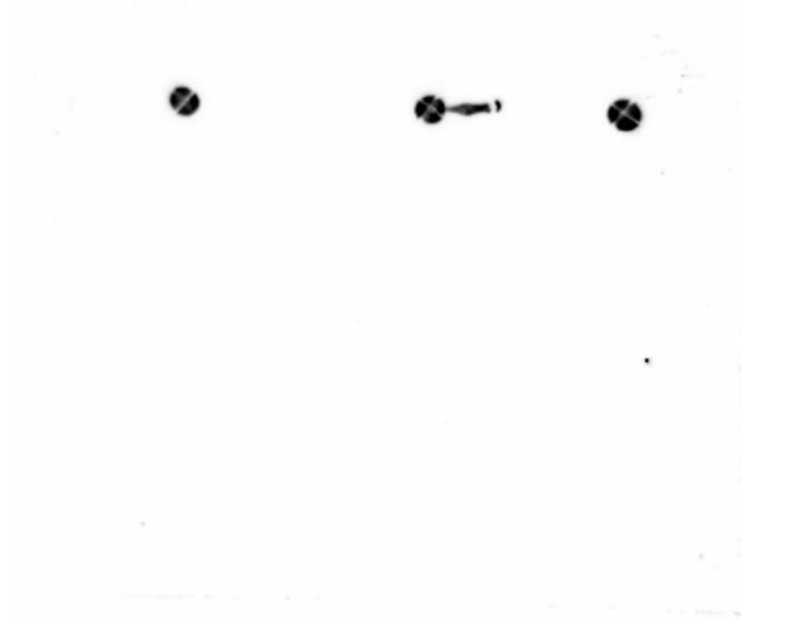	Total Histone H4
H3K27ac H3K14ac<br>**0.1µg** **0.1µg**<br><br>H3K27ac H3K14ac<br>**0.5µg** **0.5µg**<br><br>H3K27ac H3K14ac<br>**2µg** **1µg**<br><br>H4K12ac Unmodified H3<br>**1µg** **1µg**	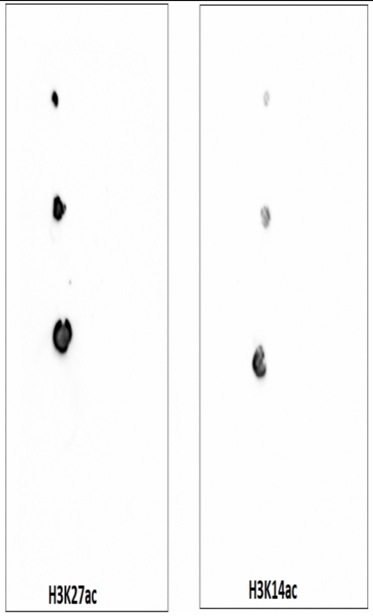	H3K27ac

<table>
<tr>
<td>H3K14ac **0.1µg**   H3K27ac **0.1µg**

H3K14ac **0.5µg**   H3K27ac **0.5µg**

H3K14ac **1µg**   H3K27ac **2µg**

H4K12ac **1µg**   Unmodified H3 **1µg**</td>
<td>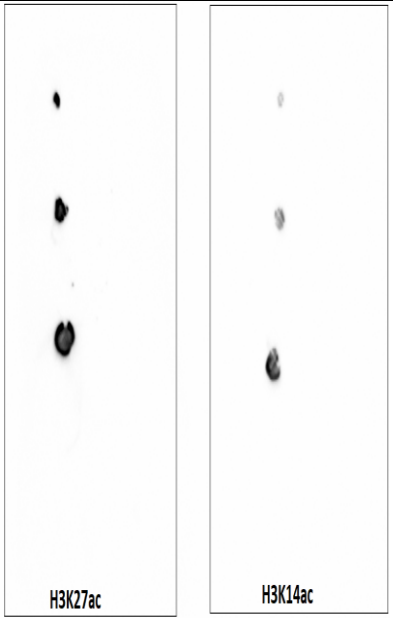
</td>
<td>H3K14ac</td>
</tr>
<tr>
<td>H3K4me-->
0.1µg   **0.5µg**   **2µg**
H3K36me3 **0.5µg**
H3K4me2-->
0.1µg   **0.5µg**   **2µg**
H3K36me3 **2µg**
H3K4me3-->
0.1µg   **0.5µg**   **2µg**
H3K27me3 **2µg**
H3K9me3 **0.5µg**   H3K9me3 **2µg**   H3K27me3 **0.5µg**</td>
<td>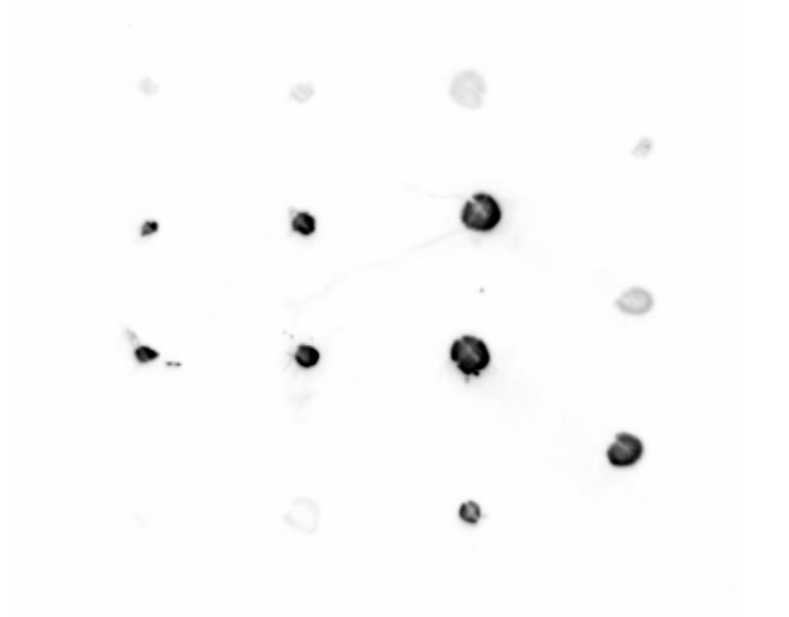</td>
<td>H3K4me3 (Abcam)</td>
</tr>
</table>

H3K27me2--->

0.1µg **0.5µg** **2µg**

H3K36me2

0.5µg

H3K27me--->

0.1µg **0.5µg** **2µg**

H3K36me2

2µg

H3K27me3--->

0.1µg **0.5µg** **2µg**

H3K9me2

2µg

H3K4me2 H3K4me2 H3K9me2

0.5µg **2µg** **0.5µg**

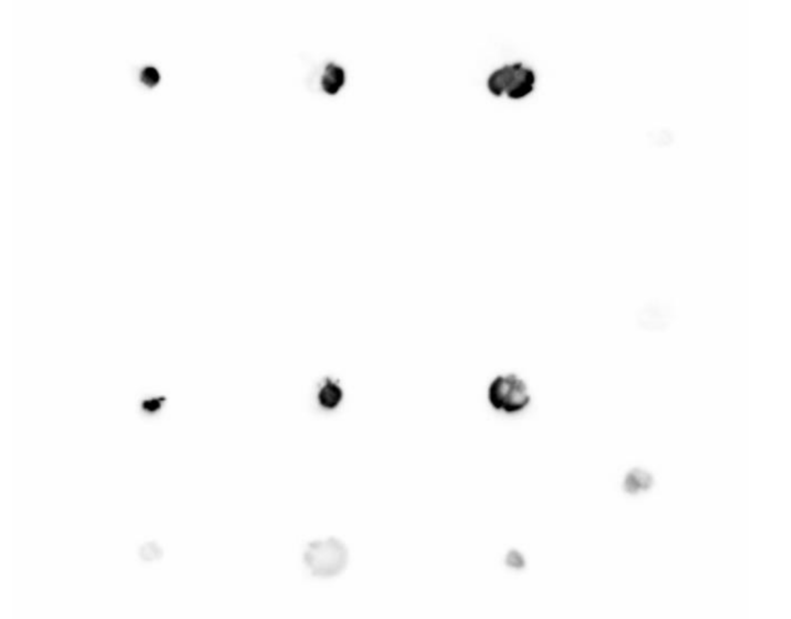

H3K27me2
