# Supplementary material for: DNA methylation and histone post-translational modification stability in post-mortem brain tissue
Source: Clin Epigenetics. 2019 Jan 11;11:5. doi: 10.1186/s13148-018-0596-7 (PMC6330433; doi:10.1186/s13148-018-0596-7)

Additional File 3

Total histone Western blot results. Raw densitometric quantity (“volume”) measurements are shown for all pigs combined (n=7; mean ± 95% confidence intervals). Total histone H3 and H4 levels were stable across all post-mortem delay time points (not significant at $p<0.05$)

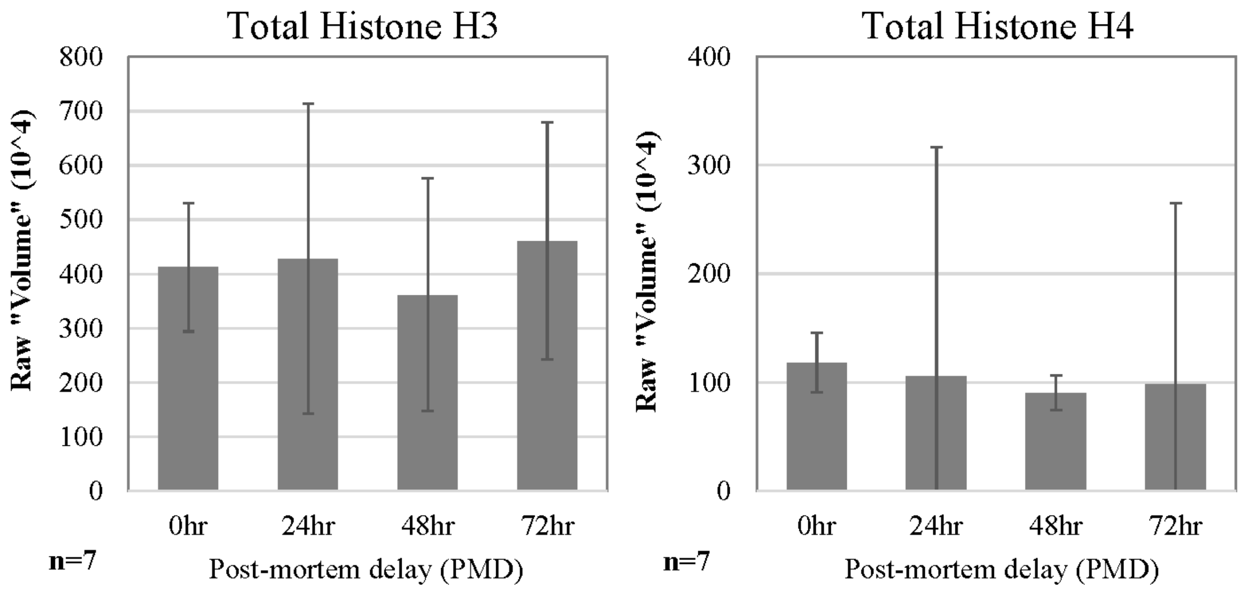

Supplement: Supplementary file 3 — Total histone Western blot results. Raw densitometric quantity (“volume”) measurements are shown for all pigs combined (n=7; mean ± 95% confidence intervals). Total histone H3 and H4 levels were stable across all post-mortem delay time points (not significant at p<0.05). (PDF 33 kb) [file 13148_2018_596_MOESM3_ESM.pdf]
