# Supplementary material for: DNA methylation and histone post-translational modification stability in post-mortem brain tissue
Source: Clin Epigenetics. 2019 Jan 11;11:5. doi: 10.1186/s13148-018-0596-7 (PMC6330433; doi:10.1186/s13148-018-0596-7)

Additional File 4

Figure S1: Western blots for each pig set of normoxic (Pigs 1-4) and hypoxic (Pigs 5-7) pigs for H3K27me3 (left) and corresponding Total Histone H3 (right).

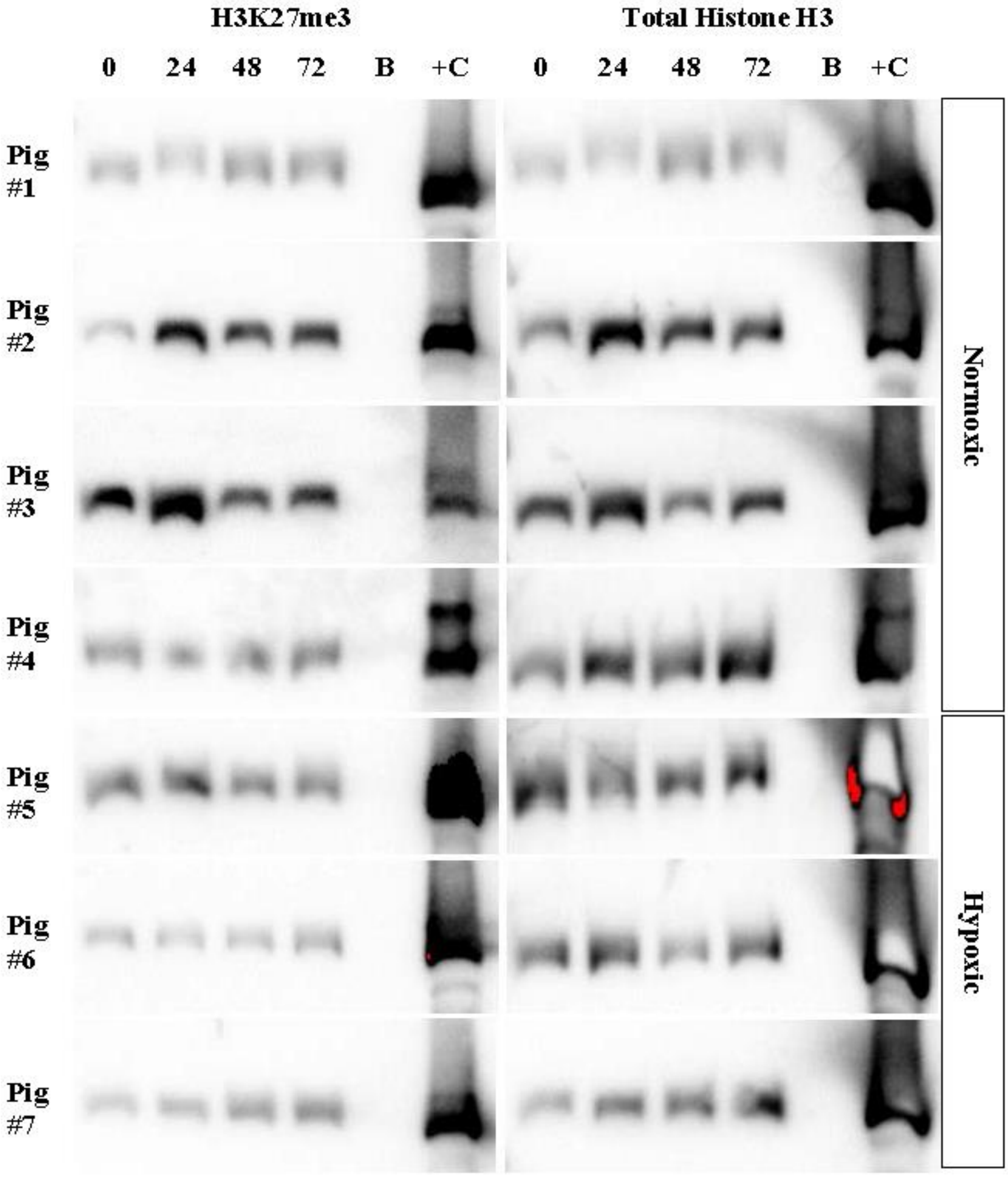

Figure S2: Western blots for each pig set of normoxic (Pigs 1-4) and hypoxic (Pigs 5-7) pigs for H4K12ac (left) and corresponding Total Histone H4 (right).

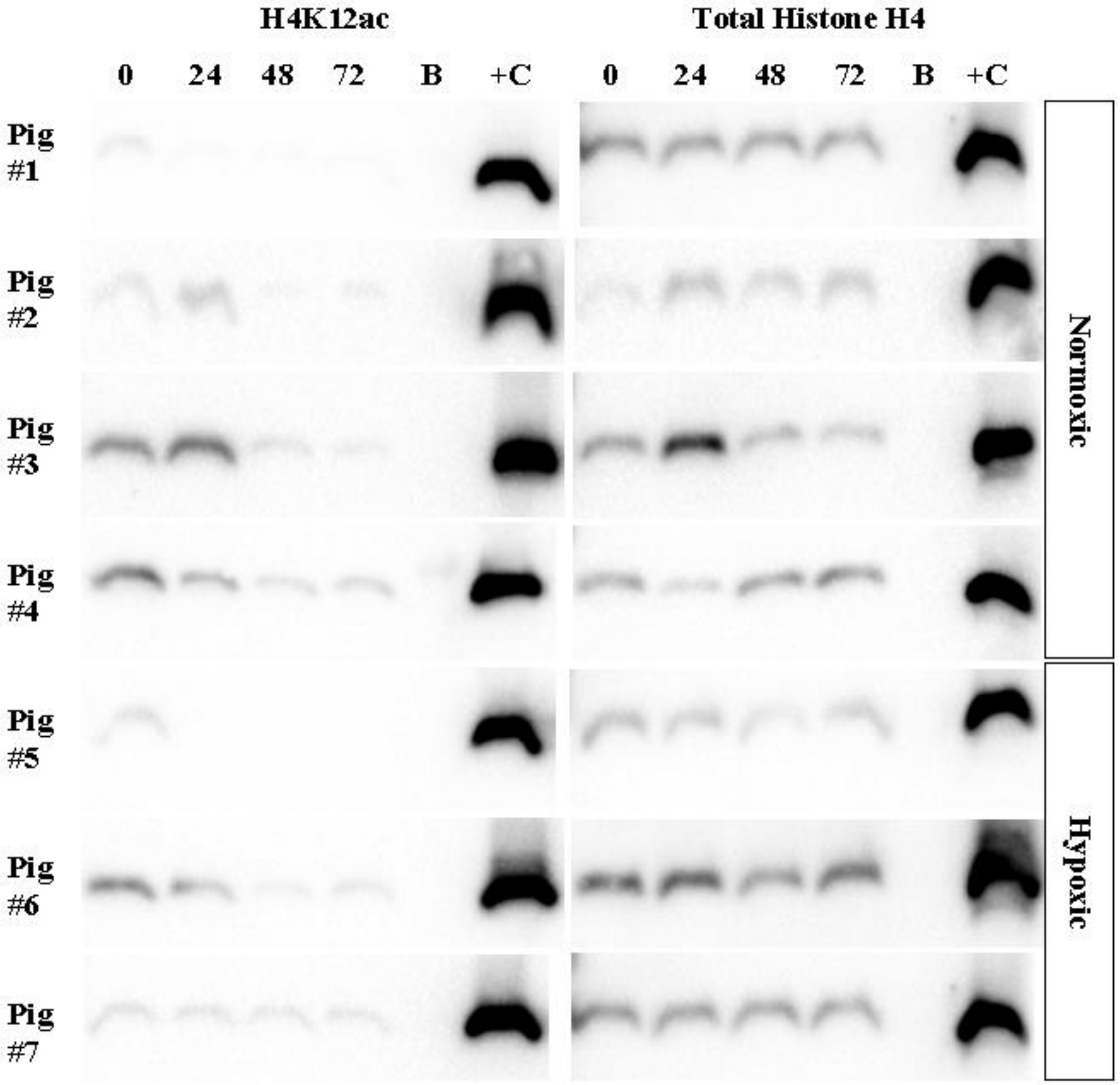

Figure S3: Western blots for Pig #7 (Hypoxic) for all histone PTMs (left) and corresponding Total Histone (right).

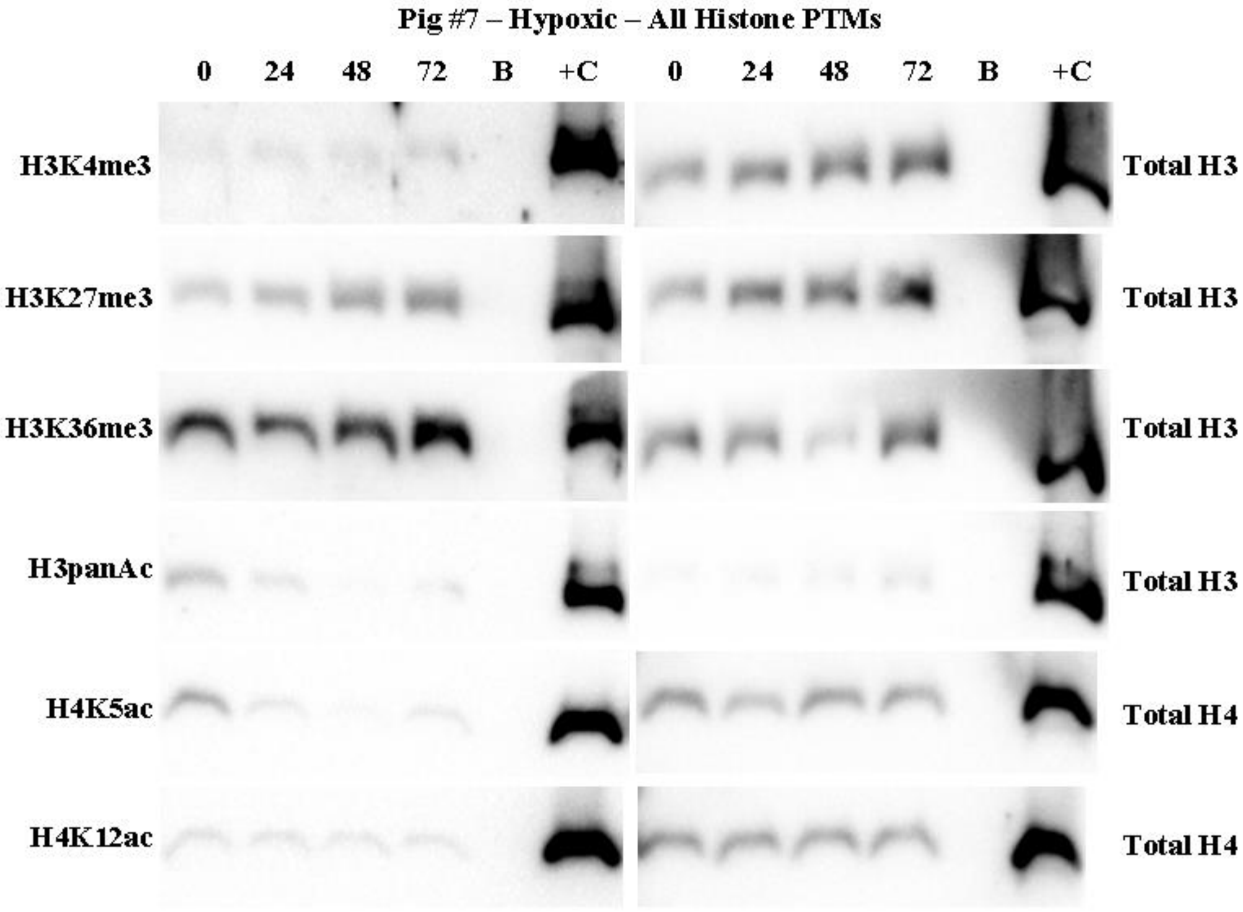

Figure S4: Full western blot images for H3K36me3 and H4K12ac. Histone PTM (left) corresponding Total Histone (right). L represents the protein standard ladder (kd), B represents a "blank" lane, +C represents the positive control (calf thymus histone mix).

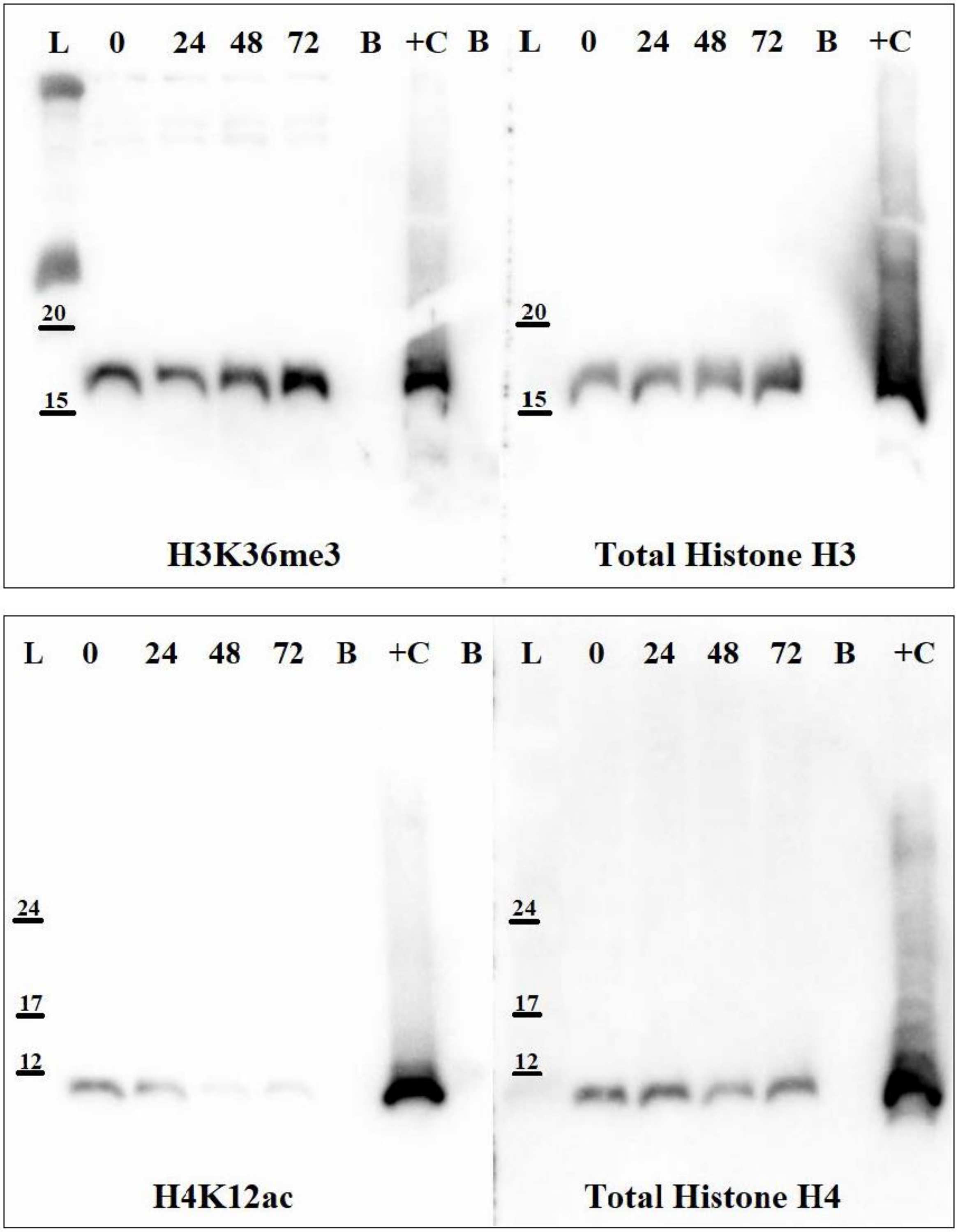

Supplement: Supplementary file 4 — Figure S1. Western blots for each pig set of normoxic (Pigs 1-4) and hypoxic (Pigs 5-7) pigs for H3K27me3 (left) and corresponding Total Histone H3 (right). Figure S2. Western blots for each pig set of normoxic (Pigs 1-4) and hypoxic (Pigs 5-7) pigs for H4K12ac (left) and corresponding Total Histone H4 (right). Figure S3. Western blots for Pig #7 (Hypoxic) for all histone PTMs (left) and corresponding Total Histone (right). Figure S4. Full western blot images for H3K36me3 and H4K12ac. Histone PTM (left) corresponding Total Histone (right). L represents the protein standard ladder (kd), B represents a “blank” lane, +C represents the positive control (calf thymus histone mix). (PDF 4534 kb) [file 13148_2018_596_MOESM4_ESM.pdf]
