# Supplementary material for: DNA methylation and histone post-translational modification stability in post-mortem brain tissue
Source: Clin Epigenetics. 2019 Jan 11;11:5. doi: 10.1186/s13148-018-0596-7 (PMC6330433; doi:10.1186/s13148-018-0596-7)
Supplement: Supplementary file 5 — Figure S1. Immunohistochemical detection of H3K27me2 in 10-day mouse and neonatal pig neocortex. In control brains (0 hour), the nuclei of all cells, except for a subpopulation of endothelial cells, are positive. Photomicrographs show decreased intensity of immunoreactivity in medium-size nuclei of mouse brain at 48 and 72 hours post-mortem, and in large neuronal nuclei of pig brain by 72 hours post-mortem. Images taken at × 400 magnification. DAB detection of antibody (brown) and hematoxylin counterstain (blue). Figure S2. Immunohistochemical detection of “total” histone H3 and H4 in neonatal pig and mouse neocortex. In control (0 hour) pig brain and 10-day and 10-week mouse brains, anti-H3 and anti-H4 label nuclei in all but a subset of endothelial cells. However, in newborn mouse brain, anti-H3 does not label the most immature cells. Photomicrographs show a minor decrease in the intensity of immunostaining of large neurons in pig brain at 72 hours post-mortem. In mouse brain at all ages, loss of H3 and H4 immunoreactivity is more substantial at 72 hours post-mortem. Images taken at × 400 magnification. DAB detection of antibody (brown) and hematoxylin counterstain (blue). (PDF 4534 kb) [file 13148_2018_596_MOESM5_ESM.pdf]

Additional File 5

Figure S1: Immunohistochemical detection of H3K27me2 in 10-day mouse and neonatal pig neocortex. In control brains (0 hour), the nuclei of all cells, except for a subpopulation of endothelial cells, are positive. Photomicrographs show decreased intensity of immunoreactivity in medium-size nuclei of mouse brain at 48 and 72 hours post-mortem, and in large neuronal nuclei of pig brain by 72 hours post-mortem. Images taken at x 400 magnification. DAB detection of antibody (brown) and hematoxylin counterstain (blue).

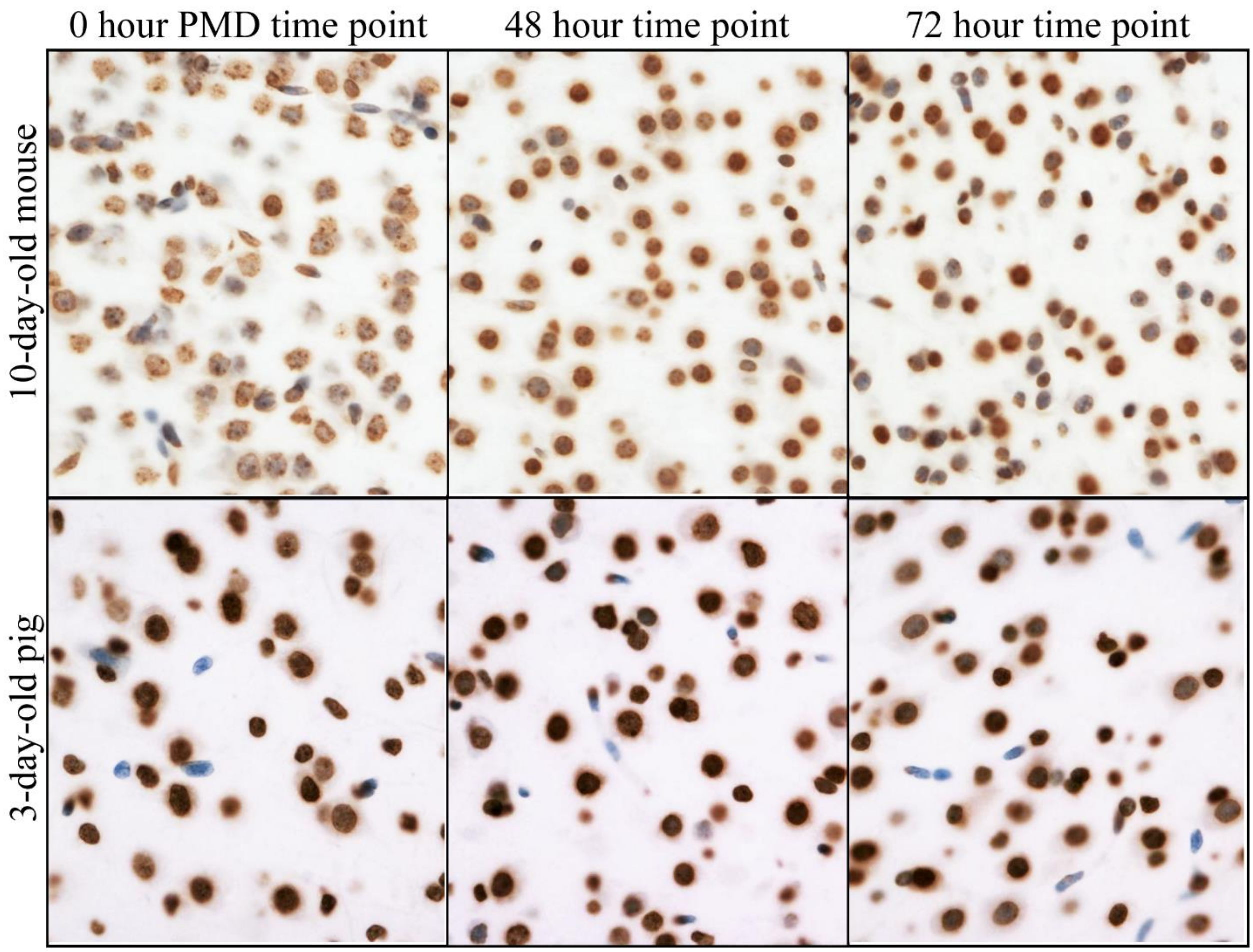

Figure S2: Immunohistochemical detection of “total” histone H3 and H4 in neonatal pig and mouse neocortex. In control (0 hour) pig brain and 10-day and 10-week mouse brains, anti-H3 and anti-H4 label nuclei in all but a subset of endothelial cells. However, in newborn mouse brain, anti-H3 does not label the most immature cells. Photomicrographs show a minor decrease in the intensity of immunostaining of large neurons in pig brain at 72 hours post-mortem. In mouse brain at all ages, loss of H3 and H4 immunoreactivity is more substantial at 72 hours post-mortem. Images taken at x 400 magnification. DAB detection of antibody (brown) and hematoxylin counterstain (blue).

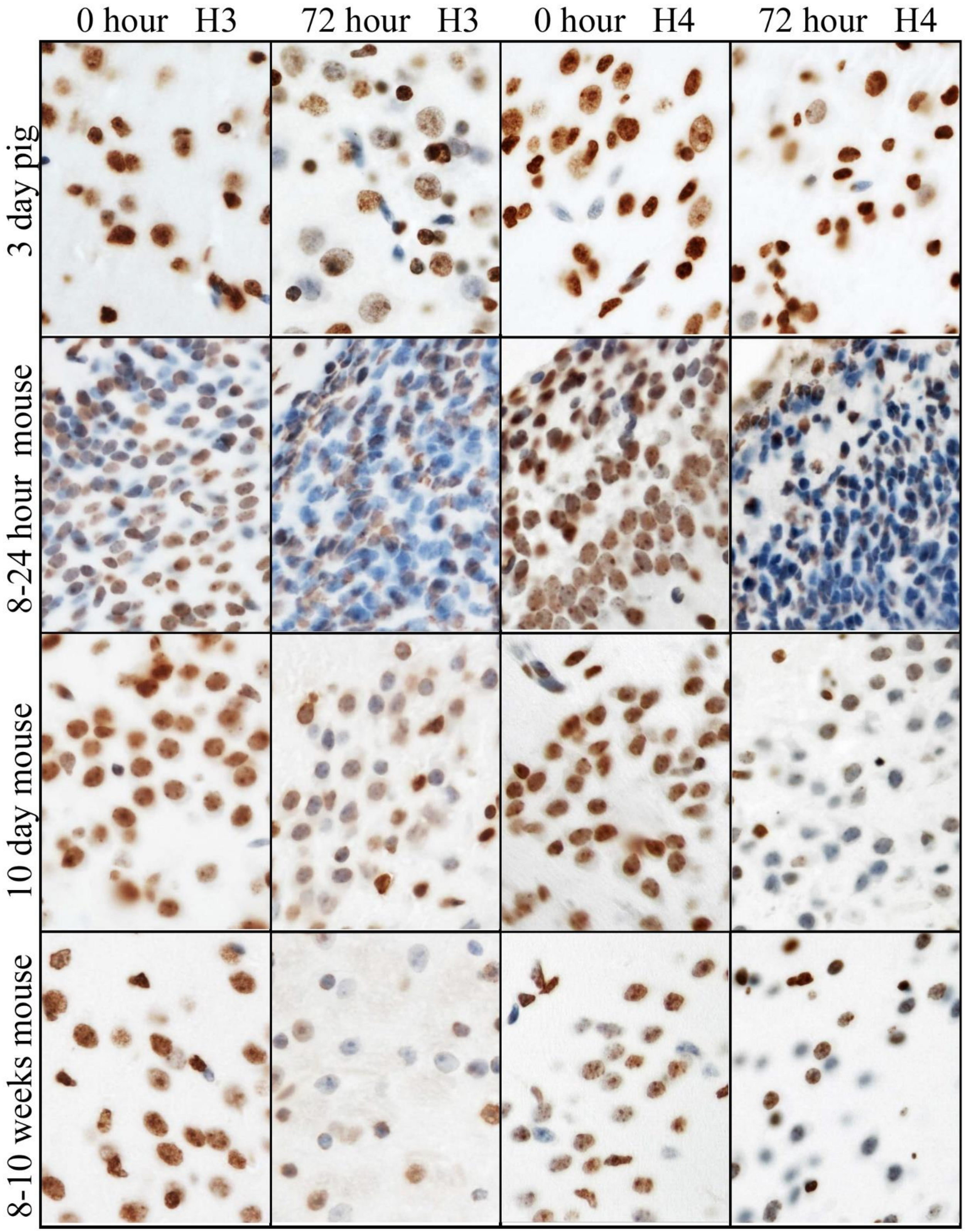
0 hour H3
72 hour H3
0 hour H4
72 hour H4
3 day pig
8-24 hour mouse
10 day mouse
8-10 weeks mouse
