# Supplementary material for: DNA methylation and histone post-translational modification stability in post-mortem brain tissue
Source: Clin Epigenetics. 2019 Jan 11;11:5. doi: 10.1186/s13148-018-0596-7 (PMC6330433; doi:10.1186/s13148-018-0596-7)
Supplement: Supplementary file 6 — Figure S1. Bar graphs showing the semiquantitative intensity scores (mean ± 95% confidence intervals; maximum 3) for all epigenetic modification antibodies used in mouse brain. There values are shown for dentate gyrus, where there were no age-related differences (all ages combined). DNA cytosine modifications, total histone H4, all histone methylation PTMs, and H3K14ac showed stable immunoreactivity up to 72 hours post-mortem. H4K5ac showed a downward trend (not statistically significant) with increasing PMD. Other acetylation marks (H3K9ac, H3K27ac, H4K12ac and H4K16ac) tended to decrease by 24 hours and were significantly decreased by 48 hours post-mortem. p values for all statistical comparisons shown at bottom. A p value <0.05 that is not bolded in red did not pass the Benjamini-Hochberg correction. Figure S2. Bar graphs showing the semiquantitative proportion scores (mean ± 95% confidence intervals; maximum 4) for all epigenetic modifications in mouse brain dentate gyrus (all ages combined). DNA cytosine modifications and histone methylation were stable from 0 to 72 hours post-mortem. Total histone H3 and H4 labeling declined gradually after 48 hours. All of the acetylation modifications showed progressive declines by 24 hours, although not all of the trends were statistically significant. p values for all statistical comparisons shown at bottom. A p value <0.05 that is not bolded in red did not pass the Benjamini-Hochberg correction. (PDF 4534 kb) [file 13148_2018_596_MOESM6_ESM.pdf]

Additional File 6

Figure S1: Bar graphs showing the semiquantitative intensity scores (mean ± 95% confidence intervals; maximum 3) for all epigenetic modification antibodies used in mouse brain. There values are shown for dentate gyrus, where there were no age-related differences (all ages combined). DNA cytosine modifications, total histone H4, all histone methylation PTMs, and H3K14ac showed stable immunoreactivity up to 72 hours post-mortem. H4K5ac showed a downward trend (not statistically significant) with increasing PMD. Other acetylation marks (H3K9ac, H3K27ac, H4K12ac and H4K16ac) tended to decrease by 24 hours and were significantly decreased by 48 hours post-mortem. P values for all statistical comparisons shown at bottom. A p value <0.05 that is not bolded in red did not pass the Benjamini-Hochberg correction.

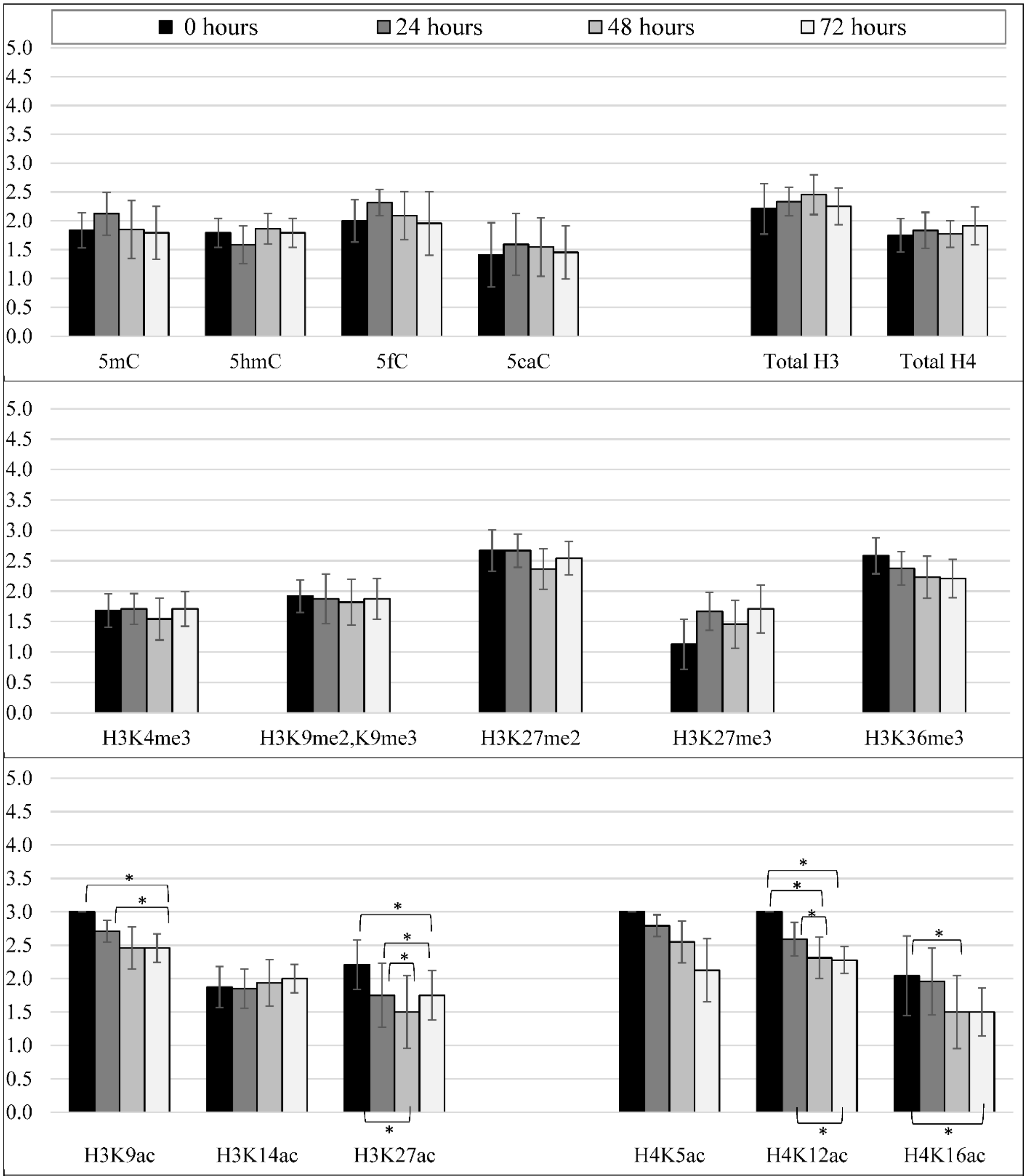

P Value	0 vs. 24	0 vs. 48	0 vs. 72	24 vs. 48	24 vs. 72	48 vs. 72
**H3K9ac**	0.25	0.0625	**0.0156**	0.125	**0.0313**	0.125
**H3K14ac**	0.5	0.5	0.5	0.5	0.5	0.375
**H3K27ac**	0.0625	**0.002**	**0.002**	**0.0156**	**0.0078**	0.1094
**H4K5ac**	0.25	0.25	0.0625	0.5	0.25	0.25
**H4K12ac**	0.5	**0.0156**	**0.0313**	**0.0156**	**0.0313**	0.5
**H4K16ac**	0.125	**0.0078**	**0.0156**	0.0625	0.0938	0.5

Figure S2: Bar graphs showing the semiquantitative proportion scores (mean ± 95% confidence intervals; maximum 4) for all epigenetic modifications in mouse brain dentate gyrus (all ages combined). DNA cytosine modifications and histone methylation were stable from 0 to 72 hours post-mortem. Total histone H3 and H4 labeling declined gradually after 48 hours. All of the acetylation modifications showed progressive declines by 24 hours, although not all of the trends were statistically significant. P values for all statistical comparisons shown at bottom. A p value <0.05 that is not bolded in red did not pass the Benjamini-Hochberg correction.

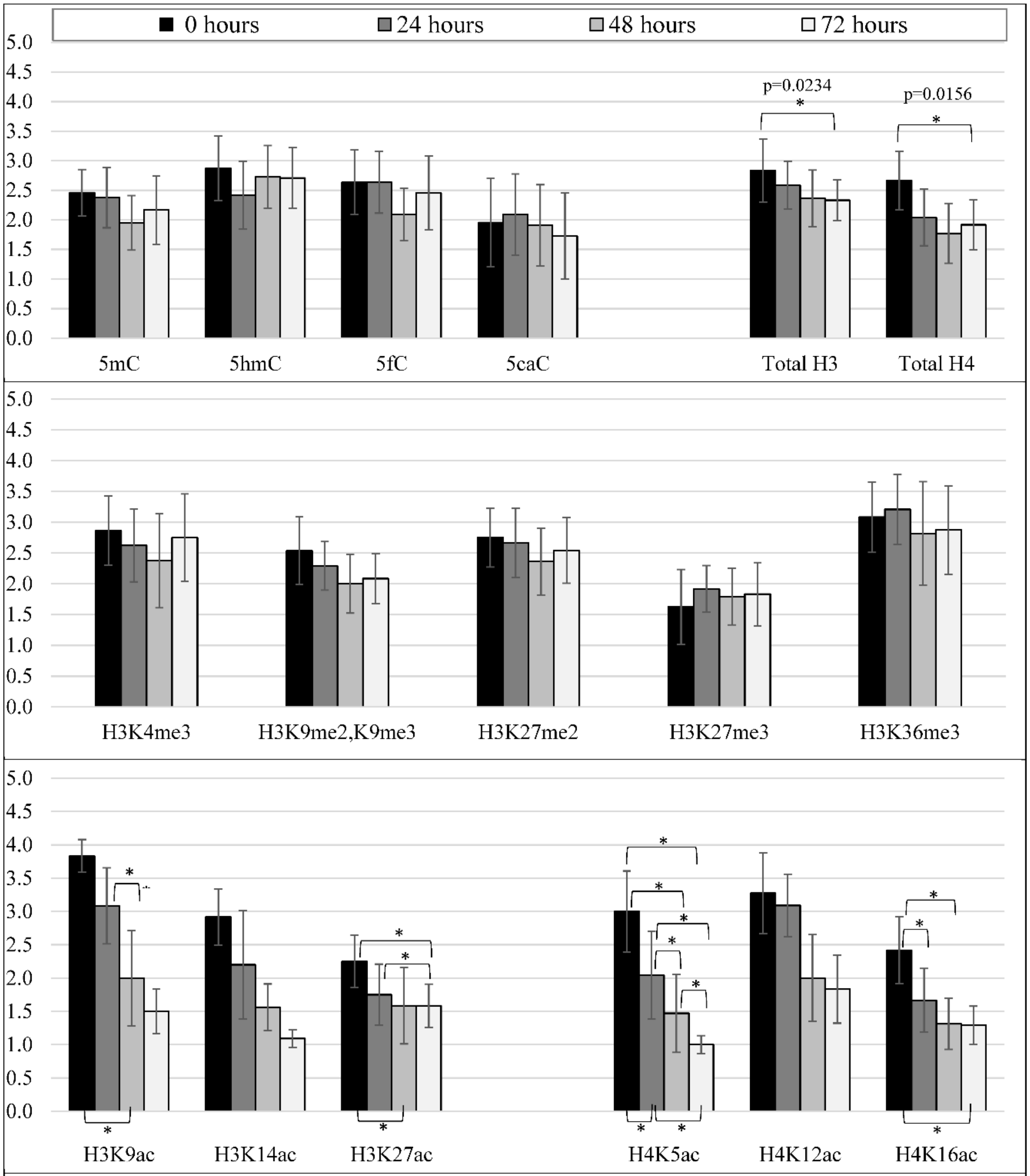

**P values**	**0 vs. 24**	**0 vs. 48**	**0 vs. 72**	**24 vs. 48**	**24 vs 72**	**48 vs 72**
**H3K9ac**	0.5	**0.0313**	0.0625	**0.0313**	0.0625	0.5
**H3K14ac**	0.5	0.5	0.3125	0.5	0.25	0.375
**H3K27ac**	0.125	**0.0313**	**0.0313**	0.25	**0.0313**	0.0625
**H4K5ac**	**0.0313**	**0.001**	**0.0005**	**0.0078**	**0.0039**	**0.0078**
**H4K12ac**	0.5	0.5	0.5	0.5	0.5	0.5
**H4K16ac**	**0.0156**	**0.0039**	**0.0078**	0.125	0.1875	0.5
