# Supplementary material for: DNA methylation and histone post-translational modification stability in post-mortem brain tissue
Source: Clin Epigenetics. 2019 Jan 11;11:5. doi: 10.1186/s13148-018-0596-7 (PMC6330433; doi:10.1186/s13148-018-0596-7)
Supplement: Supplementary file 7 — Post-mortem stability of brain antigens commonly evaluated in neuropathology. Method - Paraffin blocks of neonatal pig (one each raised in normoxic and mild hypoxic environment) cerebrum samples that were fixed with formalin after storage at 4˚C for 0, 24, 48, or 72 hours and then paraffin embedded were sectioned at 5μm thickness. They were subjected to immunostaining using and automated immunostaining system (Dako Envision) as done routinely for human autopsy specimens under conditions that had been optimized for use with human material. All slides were examined by a neuropathologist with extensive experience evaluating human and other mammalian brains. Results - There are no differences comparing normoxic to hypoxic newborn pig brains. Several antigens had no change in detection intensity, but the pattern was smudged or globular (+/- increased background) after a 72 hour delay to fixation. This suggests degradation of the cell and / or antigen leaking from cells. (PDF 4534 kb) [file 13148_2018_596_MOESM7_ESM.pdf]

Additional File 7

Post-mortem stability of brain antigens commonly evaluated in neuropathology.

Method - Paraffin blocks of neonatal pig (one each raised in normoxic and mild hypoxic environment) cerebrum samples that were fixed with formalin after storage at 4˚C for 0, 24, 48, or 72 hours and then paraffin embedded were sectioned at 5µm thickness. They were subjected to immunostaining using an automated immunostaining system (Dako Envision) as done routinely for human autopsy specimens under conditions that had been optimized for use with human material.

All slides were examined by a neuropathologist with extensive experience evaluating human and other mammalian brains.

Results - There are no differences comparing normoxic to hypoxic newborn pig brains. Several antigens had no change in detection intensity, but the pattern was smudged or globular (+/- increased background) after a 72 hour delay to fixation. This suggests degradation of the cell and / or antigen leaking from cells.

Antigen	Antibody; source	Labeling pattern in control brain tissue	Post-mortem change
Amyloid beta (A4) precursor protein (APP)	mouse monoclonal clone 22C11; Millipore MAB348	Cytoplasm of large neurons	No difference at 72 hours
Calbindin 2 (calretinin)	mouse monoclonal clone DAK-Calret 1; Agilent / Dako M7245	Cytoplasm of inhibitory interneurons	No difference at 72 hours
Glial fibrillary acidic protein (GFAP)	rabbit polyclonal; Agilent / Dako Z03344	Intermediate filaments in astrocyte cell processes	Intensity maintained at 72 hours; cell processes irregular with increased background stain at 72 hours (suggests cell degradation or antigen leak)
Neurofilament (NF)(phosphorylated light (68-70kD) and heavy chains);	mouse monoclonal clone 2F11; Agilent / Dako IR607	Axons in gray and white matter	Intensity maintained at 72 hours; axons progressively more globular beginning at 48 hours (suggests axon degradation)
Neurofilament (npNF) (nonphosphorylated heavy and medium chains)	mouse monoclonal IgG1 and IgM cocktail; Biolegend / Sternberger SMI-311	Scattered large neurons + faint in axons	No definite difference at 72 hours

Antigen	Antibody; source	Labeling pattern in control brain tissue	Post-mortem change
NeuN (RNA binding protein fox-1 homolog, RBFOX3)	mouse monoclonal clone A60; Millipore MAB377	Neurons (many types; nucleus +/- cytoplasm)	Slight decrease in intensity of normoxic but not hypoxic at 72 hours; note that this antigen seems to be sensitive to overfixation in human tissue
Neuron specific enolase (NSE; enolase 2)	mouse monoclonal clone BBS/NC/VI-H14; Agilent / Dako M0873	Some neuron cell bodies + neuropil	Slight fading of cytoplasm and increased background at 72 hours
Synaptophysin	mouse monoclonal clone 27G12; Novocastra / Leica ORG- 8848	Presynaptic protein (neuropil pattern)	No loss of intensity at 72 hours, but pattern looks smudged (suggests antigen leak)
S100 (calcium binding protein beta)	rabbit polyclonal; Agilent / Dako Z0311	Glial nuclei, cell bodies (mainly astrocytes), and processes near cell bodies	No loss of intensity at 72 hours, but pattern looks smudged in glial processes at 72 hours (suggests antigen leak)
TDP-43 (TARDBP)	rabbit polyclonal; Proteintech 10782-2-AP	Neuron (and some glial) nuclei	Faded slightly at 48 hours and more at 72 hours (but labeling still obvious)
Nkx2-1 (TTF1)	mouse monoclonal clone SPT24; Novocastra / Leica NCL-L-TTF-1	Nuclei of rare precursor cells	Slightly decreased intensity in some cells at 72 hours
Vimentin	mouse monoclonal clone V9; Agilent / Dako M0725	Intermediate filaments in endothelia and some astrocytes	No loss of intensity at 72 hours
