# Supplementary material for: DNA methylation and histone post-translational modification stability in post-mortem brain tissue
Source: Clin Epigenetics. 2019 Jan 11;11:5. doi: 10.1186/s13148-018-0596-7 (PMC6330433; doi:10.1186/s13148-018-0596-7)
Supplement: Supplementary file 8 — Figure S1. Pig brain dissection. Lines on the photograph show the dissection planes used to divide the pig brain into samples used for formalin fixation and subsequent immunohistochemical staining (white) or freezing at -70˚C and subsequent biochemical analyses (yellow). Figure S2. Mouse brain dissection. The left brain hemisphere was used for formalin fixation (FFPE) and subsequent immunohistochemical staining and the right hemisphere for freezing at -70˚C. (PDF 4534 kb) [file 13148_2018_596_MOESM8_ESM.pdf]

Additional File 8

Figure S1: Pig brain dissection. Lines on the photograph show the dissection planes used to divide the pig brain into samples used for formalin fixation and subsequent immunohistochemical staining (white) or freezing at -70˚C and subsequent biochemical analyses (yellow).

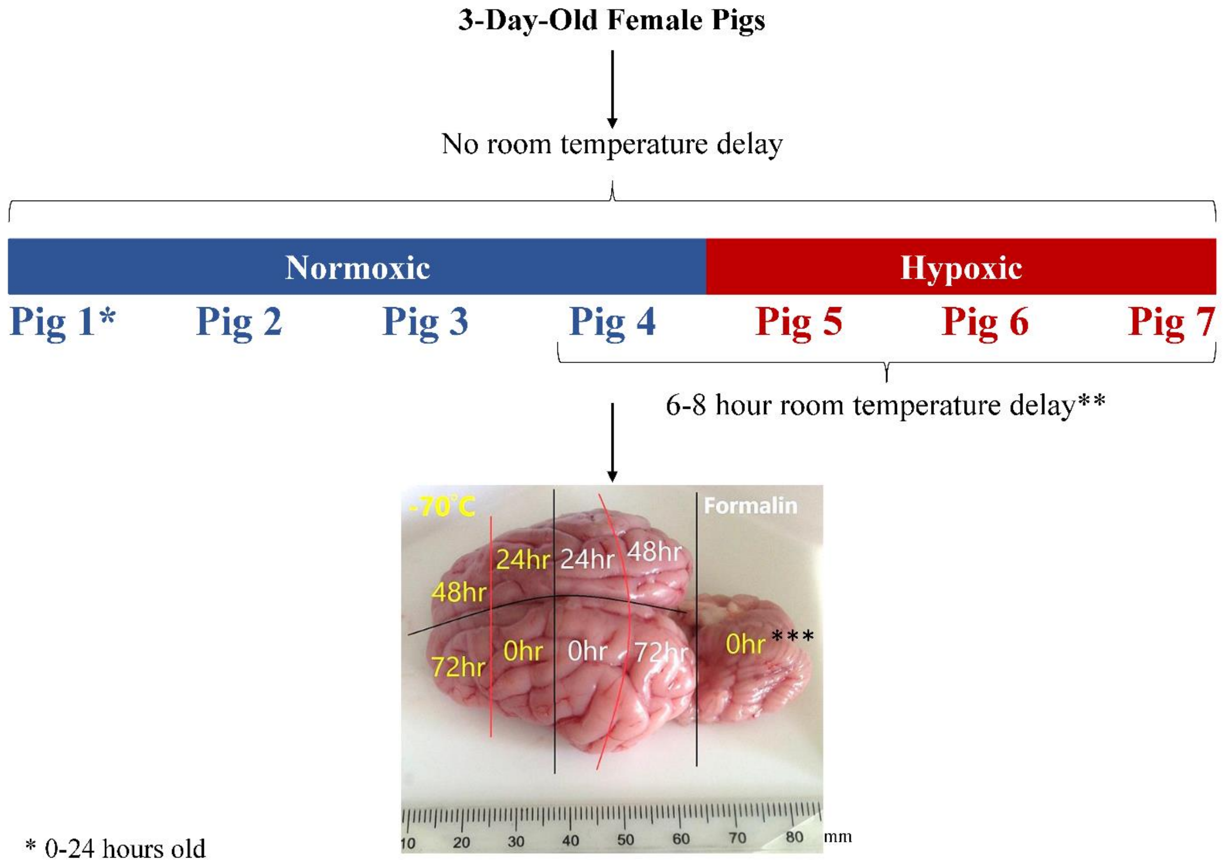

* 0-24 hours old

** each post-mortem delay brain section was further cut into 2; one section had no room temperature delay and the other had a room temperature delay

*** the cerebellum and brainstem were removed and frozen as practice tissue from assay optimization

Figure S2: Mouse brain dissection. The left brain hemisphere was used for formalin fixation (FFPE) and subsequent immunohistochemical staining and the right hemisphere for freezing at -70˚C.

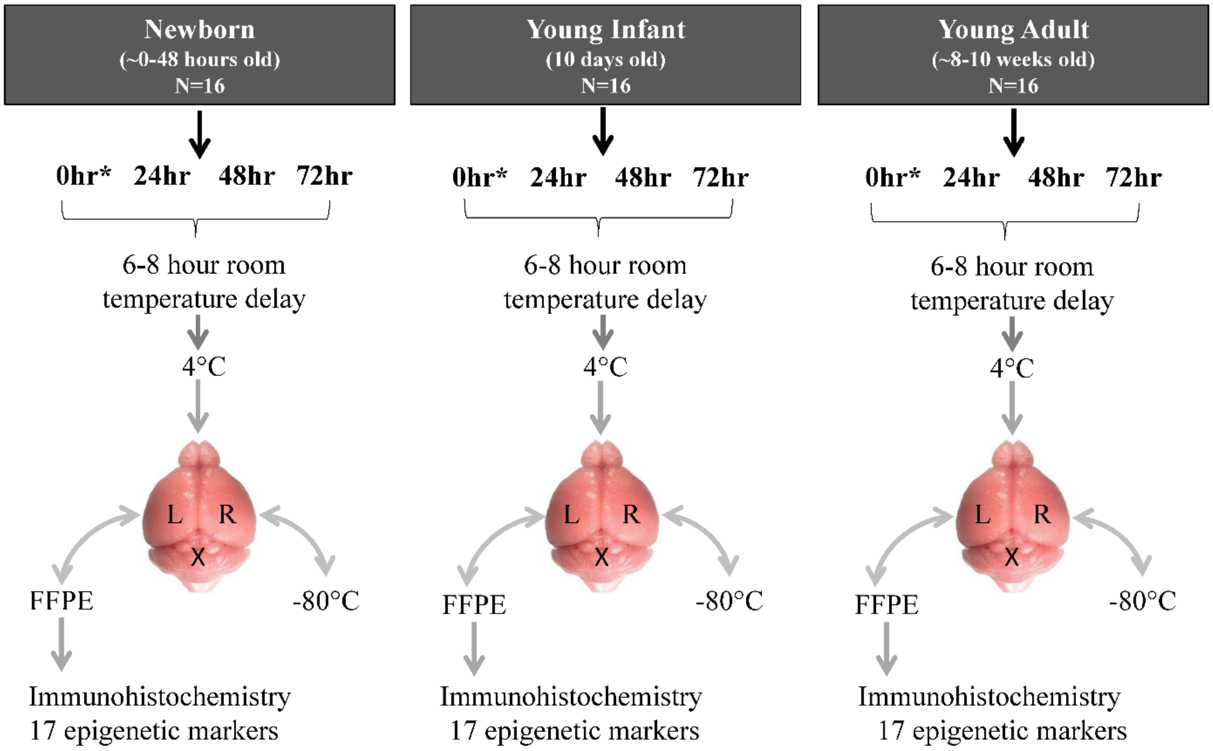

*0 hour time point was processed immediately
The cerebellum was removed and discarded which is represented by an "X"
FFPE = Formalin-fixed paraffin-embedded
