# Supplementary material for: DNA methylation and histone post-translational modification stability in post-mortem brain tissue
Source: Clin Epigenetics. 2019 Jan 11;11:5. doi: 10.1186/s13148-018-0596-7 (PMC6330433; doi:10.1186/s13148-018-0596-7)
Supplement: Supplementary file 9 — Human brain tissue microarray - case details and construction. (PDF 97 kb) [file 13148_2018_596_MOESM9_ESM.pdf]

Additional File 9 - Human brain tissue microarray - case details and construction

Slide Position	Brain region	Age / sex	Description (a)	Time delay between tissue death & start of fixation	Fixation duration
1	Cerebellum	17y, male	Autopsy - normal	24 hours	4 days
2	Temporal cortex	72y, male	Normal tissue adjacent to tumor	<1 hour	1 days
3	Temporal cortex	12y, male	Normal tissue adjacent to hippocampus removed for epilepsy procedure	<1 hour	1 days
4	Frontal cortex	66y, female	Autopsy - normal	6 hours	14 days
5	Frontal Cortex	17y, male	Autopsy - normal	24 hours	4 days
6	Frontal Cortex	8m, male	Autopsy - normal (sudden infant death)	26 hours	8 days
7	Frontal Cortex	42y, male	Autopsy - severe hypoxia (cardiac arrest) + 7 day survival before death	31 hours	19 days
8	Frontal Cortex	53y, male	Autopsy - severe hypoxia + 2 day survival before death	43 hours	19 days
9	Frontal Cortex	10m, male	Autopsy - normal (sudden infant death)	52 hours	2 days
10	Frontal Cortex	15y, female	Autopsy - normal	53 hours	1 days
11	Frontal Cortex	21y, male	Autopsy - normal	3 days	17 days
12	Frontal Cortex	61y, male	Autopsy - no antemortem pathology; body partially decomposed with deep brain putrefaction	3 days +	18 days
13	Frontal Cortex	17y, male	Autopsy - acute brain trauma with immediate death - uninvolved brain	4 days	2 days
14	Frontal Cortex	76y, female	Autopsy - Alzheimer disease	5 days	55 days

Slide layout

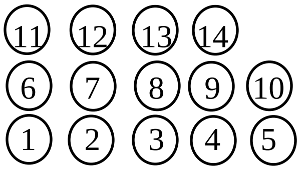

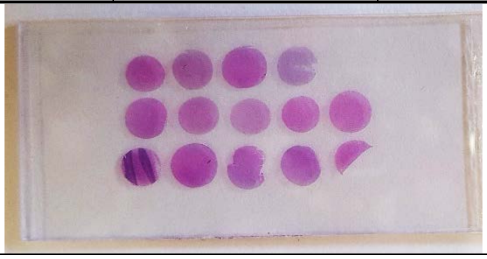

a) Sample 1 (cerebellum) is for orientation only. Samples 2 and 3 (temporal cortex) were obtained from routine neurosurgical specimens. All autopsy cases were handled in the routine manner with storage of the body at 4˚C beginning ~4-8 hours after death; case 12 is the exception with the body remaining at room temperature 1-2 days prior to cooling. During autopsy, brains were removed and placed in formalin whole within 30 minutes.
