# Supplementary material for: DNA methylation and histone post-translational modification stability in post-mortem brain tissue
Source: Clin Epigenetics. 2019 Jan 11;11:5. doi: 10.1186/s13148-018-0596-7 (PMC6330433; doi:10.1186/s13148-018-0596-7)
Supplement: Supplementary file 10 — Epigenetic mark antibody details and sources. (PDF 4535 kb) [file 13148_2018_596_MOESM10_ESM.pdf]

Additional File 10: Epigenetic mark antibody details and sources.

Antibody	Details	Isotype	Species	Type	Company Details	Lot #
5mC	Clone 33D3 recognizes the modified base 5-methylcytidine found in plant and vertebrate DNA.	IgG	Mouse	Monoclonal	Active Motif, #39649	8614012<br>24516019
5hmC	Raised against 5-hydroxymethylcytidine conjugated to KLH and recognizes 5-hydroxymethylcytosine.	Serum	Rabbit	Polyclonal	Active Motif, #39769	10210001<br>06116002
5fC	Raised against 5-formylcytidine conjugated to KLH and recognizes 5-formylcytosine.	Serum	Rabbit	Polyclonal	Active Motif, #61223	34711001
5caC	Raised against 5-carboxylcytidine conjugated to KLH and recognizes 5-carboxylcytosine.	Serum	Rabbit	Polyclonal	Active Motif, #61225	32115002
H3K4me3	Raised against a peptide including trimethyl-lysine 4 of histone H3.	Serum	Rabbit	Polyclonal	Active Motif, #39159	12613005
H3K4me3	Synthetic peptide within Human Histone H3 aa 1-100 (tri methyl K4) conjugated to Keyhole Limpet Haemocyanin (KLH). The exact sequence is proprietary.	IgG	Rabbit	Polyclonal	Abcam, #ab8580	GR144288-1<br>GR224369-1<br>GR240214-4
H3K9ac	Synthetic peptide corresponding to Human Histone H3 aa 1-100 (N terminal) (acetyl K9) conjugated to Keyhole Limpet Haemocyanin (KLH).	IgG	Rabbit	Polyclonal	Abcam, #ab10812	GR287797-1
H3K9me2, K9me3	Synthetic peptide within Human Histone H3 aa 1-100 (tri methyl K9). The exact sequence is proprietary.	IgG1	Mouse	Monoclonal	Abcam, #ab71604	GR117765-1
H3K14ac	Synthetic peptide within Human Histone H3 (acetyl K14). The exact sequence is proprietary.	IgG	Rabbit	Monoclonal	Abcam #ab52946	GR149741-17<br>GR149741-20<br>GR302893-8
H3K27ac	Synthetic peptide corresponding to Human Histone H3 aa 1-100 (acetyl K27) conjugated to Keyhole Limpet Haemocyanin (KLH).	IgG	Rabbit	Polyclonal	Abcam, #ab4729	GR81163-1<br>GR288020-1
H3K27me2	Synthetic peptide corresponding to Human Histone H3 aa 1-100 (N Terminal) (di methyl K27) conjugated to Keyhole Limpet Haemocyanin (KLH).	IgG	Rabbit	Polyclonal	Abcam, #ab24684	GR31501-6<br>GR31501-1

Antibody	Details	Isotype	Species	Type	Company Details	Lot #
H3K27me3	Synthetic peptide within Human Histone H3 aa 1-100 (tri methyl K27) conjugated to keyhole limpet haemocyanin (Sulfosuccinimidyl 4-N-maleimidomethyl-cyclohexane-1-carboxylate (Sulfo-SMCC)). The exact sequence is proprietary.	IgG fraction	Mouse	Monoclonal	Abcam, #ab6002	GR218433-5<br>GR275911-1<br>GR275911-2<br>GR275911-7
H3K36me3	Synthetic peptide within Human Histone H3 aa 1-100 (tri methyl K36) conjugated to Keyhole Limpet Haemocyanin (KLH). The exact sequence is proprietary.	IgG	Rabbit	Polyclonal	Abcam, #ab9050	GR249065-1<br>GR3177961-1
H4K5ac	Synthetic peptide within Human Histone H4 aa 1-100 (N terminal) (acetyl K5). The exact sequence is proprietary.	IgG	Rabbit	Monoclonal	Abcam, #ab51997	GR295999-2<br>GR295999-7
H4K12ac	Synthetic peptide corresponding to Human Histone H4 aa 10-15 (acetyl K12). Synthetic acetylated peptide derived from human Histone H4 around the acetylation site of lysine 12 LGK(Ac)GG	IgG	Rabbit	Polyclonal	Abcam, #ab61238	GR325039-1<br>GR325039-3
H4K16ac	Synthetic peptide (the amino acid sequence is considered to be commercially sensitive) corresponding to Human Histone H4 (acetyl K16).	IgG	Rabbit	Monoclonal	Abcam, #ab109463	GR53193-15<br>GR187780-8
H3panAc	This antibody was raised against a peptide including acetyl-lysines contained in the N-terminal tail of human Histone H3.	IgG	Rabbit	Polyclonal	Active Motif, #61637	28915002
Total H3	Raised against a peptide containing the N-terminus of histone H3.	IgG2b	Mouse	Monoclonal	Active Motif, #39763	07716018<br>34614001<br>5217020
Total H4	Raised against a synthetic peptide containing human Histone H4.	IgG2b	Mouse	Monoclonal	Active Motif, #61521	24615004<br>7715006
