# Supplementary material for: DNA methylation and histone post-translational modification stability in post-mortem brain tissue
Source: Clin Epigenetics. 2019 Jan 11;11:5. doi: 10.1186/s13148-018-0596-7 (PMC6330433; doi:10.1186/s13148-018-0596-7)
Supplement: Supplementary file 11 — Table S1. Antibody dilutions and respective Western Blotting conditions. Table S2. Antibody dilutions and respective Immunohistochemistry conditions. (PDF 4535 kb) [file 13148_2018_596_MOESM11_ESM.pdf]

Additional File 11

Table S1: Antibody dilutions and respective Western Blotting conditions

Antibody	Manufactured Concentration	Dilution	Blocking Solution
H3K4me3	n/a	1:600	BSA
H3K27me3	1μg/μl	1:1000	BSA
H3K36me3	0.2μg/μl	1:200	BSA
H4K5ac	0.523μg/μl	1:1000	Skim Milk
H4K12ac	1μg/μl	1:1000	Skim Milk
H3panAc	1μg/μl	1:1000	Skim Milk
Total H3	0.68μg/μl	1:5000	Skim Milk or BSA
Total H4	0.52μg/μl	1:2000	Skim Milk

BSA - Bovine Serum Albumin

Table S2: Antibody dilutions and respective Immunohistochemistry conditions

Antibody	Dilution	DAB Time	Antigen Retrieval	Ab Diluent	Washing Buffer #1	Washing Buffer #2
5mC	1:500	4min	Sodium Citrate pH 6.0	1% BSA in PBS	1X PBS + 0.2% Triton-X-100	1X PBS
5hmC	1:3000	5min	Tris-EDTA pH 9.0	1% BSA in TBS	1X TBS + 0.1% Tween 20	1X TBS
5fC	1:400	5min	Tris-EDTA pH 9.0	1% BSA in TBS	1X TBS + 0.1% Tween 20	1X TBS
5caC	1:250	5min	Tris-EDTA pH 9.0	1% BSA in TBS	1X TBS + 0.1% Tween 20	1X TBS
H3K4me3	1:500	5min	Sodium Citrate pH 6.0	1% BSA in PBS	1X PBS + 0.2% Triton-X-100	1X PBS
H3K9ac	1:500	5min	Tris-EDTA pH 9.0	1% BSA in TBS	1X TBS + 0.1% Tween 20	1X TBS
H3K9me2, K9me3	1:700	4min	Sodium Citrate pH 6.0	1% BSA in PBS	1X PBS + 0.2% Triton-X-100	1X PBS
H3K14ac	1:150	5min	Sodium Citrate pH 6.0	1% BSA in PBS	1X PBS + 0.2% Triton-X-100	1X PBS
H3K27ac	1:500	5min	Sodium Citrate pH 6.0	1% BSA in PBS	1X PBS + 0.2% Triton-X-100	1X PBS
H3K27me2	1:700	4min	Sodium Citrate pH 6.0	1% BSA in PBS	1X PBS + 0.2% Triton-X-100	1X PBS

**Antibody**	**Dilution**	**DAB Time**	**Antigen Retrieval**	**Ab Diluent**	**Washing Buffer #1**	**Washing Buffer #2**
H3K27me3	1:150	5min	Sodium Citrate pH 6.0	1% BSA in PBS	1X PBS + 0.2% Triton-X-100	1X PBS
H3K36me3	1:100	6mins	Tris-EDTA pH 9.0	1% BSA in TBS	1X TBS + 0.1% Tween 20	1X TBS
H4K5ac	1:350	5min	Tris-EDTA pH 9.0	1% BSA in TBS	1X TBS + 0.1% Tween 20	1X TBS
H4K12ac	1:300	5min	Tris-EDTA pH 9.0	1% BSA in TBS	1X TBS + 0.1% Tween 20	1X TBS
H4K16ac	1:100	6min	Sodium Citrate pH 6.0	1% BSA in PBS	1X PBS + 0.2% Triton-X-100	1X PBS
H3panAc	1.200	5min	Sodium Citrate pH 6.0	1% BSA in PBS	1X PBS + 0.2% Triton-X-100	1X PBS
Total H3	1:300	6min	Sodium Citrate pH 6.0	1% BSA in PBS	1X PBS + 0.2% Triton-X-100	1X PBS
Total H4	1:1000	5min	Tris-EDTA pH 9.0	1% BSA in TBS	1X TBS + 0.1% Tween 20	1X TBS

PBS – phosphate buffered saline

TBS – tris-buffered saline
