# Supplementary material for: DNA methylation and histone post-translational modification stability in post-mortem brain tissue
Source: Clin Epigenetics. 2019 Jan 11;11:5. doi: 10.1186/s13148-018-0596-7 (PMC6330433; doi:10.1186/s13148-018-0596-7)
Supplement: Supplementary file 12 — Peptides used for antibody specificity experiments. (PDF 39 kb) [file 13148_2018_596_MOESM12_ESM.pdf]

Additional File 12

Peptides used for antibody specificity experiments

Peptide Name		Company	Catalogue #	Manufactured Concentration
H3K4me	Human Histone H3 (mono methyl K4) synthetic peptide	Abcam	ab1340	1mg/ml
H3K4me2	Human Histone H3 (di methyl K4) synthetic peptide	Abcam	ab7768	1mg/ml
H3K4me3	Human Histone H3 (tri methyl K4) synthetic peptide	Abcam	ab1342	1mg/ml
H3K9me	Human Histone H3 (mono methyl K9) synthetic peptide	Abcam	ab1771	1mg/ml
H3K9me2	Human Histone H3 (di methyl K9) synthetic peptide	Abcam	ab1772	1mg/ml
H3K9me3	Human Histone H3 (tri methyl K9) synthetic peptide	Abcam	ab1773	1mg/ml
H3K27me	Human Histone H3 (mono methyl K27) synthetic peptide	Abcam	ab1780	1mg/ml
H3K27me2	Human Histone H3 (di methyl K27) synthetic peptide	Abcam	ab1781	1mg/ml
H3K27me3	Human Histone H3 (tri methyl K27) synthetic peptide	Abcam	ab1782	1mg/ml
H3K36me	Human Histone H3 (mono methyl K36) synthetic peptide	Abcam	ab1783	1mg/ml
H3K36me2	Human Histone H3 (di methyl K36) synthetic peptide	Abcam	ab1784	1mg/ml
H3K36me3	Human Histone H3 (tri methyl K36) synthetic peptide	Abcam	ab1785	1mg/ml
H3K27ac	Human Histone H3 (acetyl K27) synthetic peptide	Abcam	ab24404	1mg/ml
H4K5ac	Human Histone H4 (acetyl K5) synthetic peptide	Abcam	ab154430	1mg/ml
H4K12ac	Human Histone H4 (acetyl K12) synthetic peptide	Abcam	ab154463	0.5mg/ml
Unmodified H3	Human Histone H3 (unmodified) synthetic peptide	Abcam	ab7228	1mg/ml
Unmodified H4	Human Histone H4 synthetic peptide (unmodified)	Abcam	ab14963	1mg/ml
