# Supplementary material for: DNA methylation and histone post-translational modification stability in post-mortem brain tissue
Source: Clin Epigenetics. 2019 Jan 11;11:5. doi: 10.1186/s13148-018-0596-7 (PMC6330433; doi:10.1186/s13148-018-0596-7)

Additional File 13:

Standard scale images used for grading the proportion of immunoreactive nuclei. Any degree of brown was considered positive. Photomicrographs show: 4 - ~100% positive; 3 - ~75% positive; 2 - ~50% positive; 1 ~25% positive; 0 - ~0% positive. Images taken at x 400 magnification. DAB detection of antibody (brown) and hematoxylin counterstain (blue).

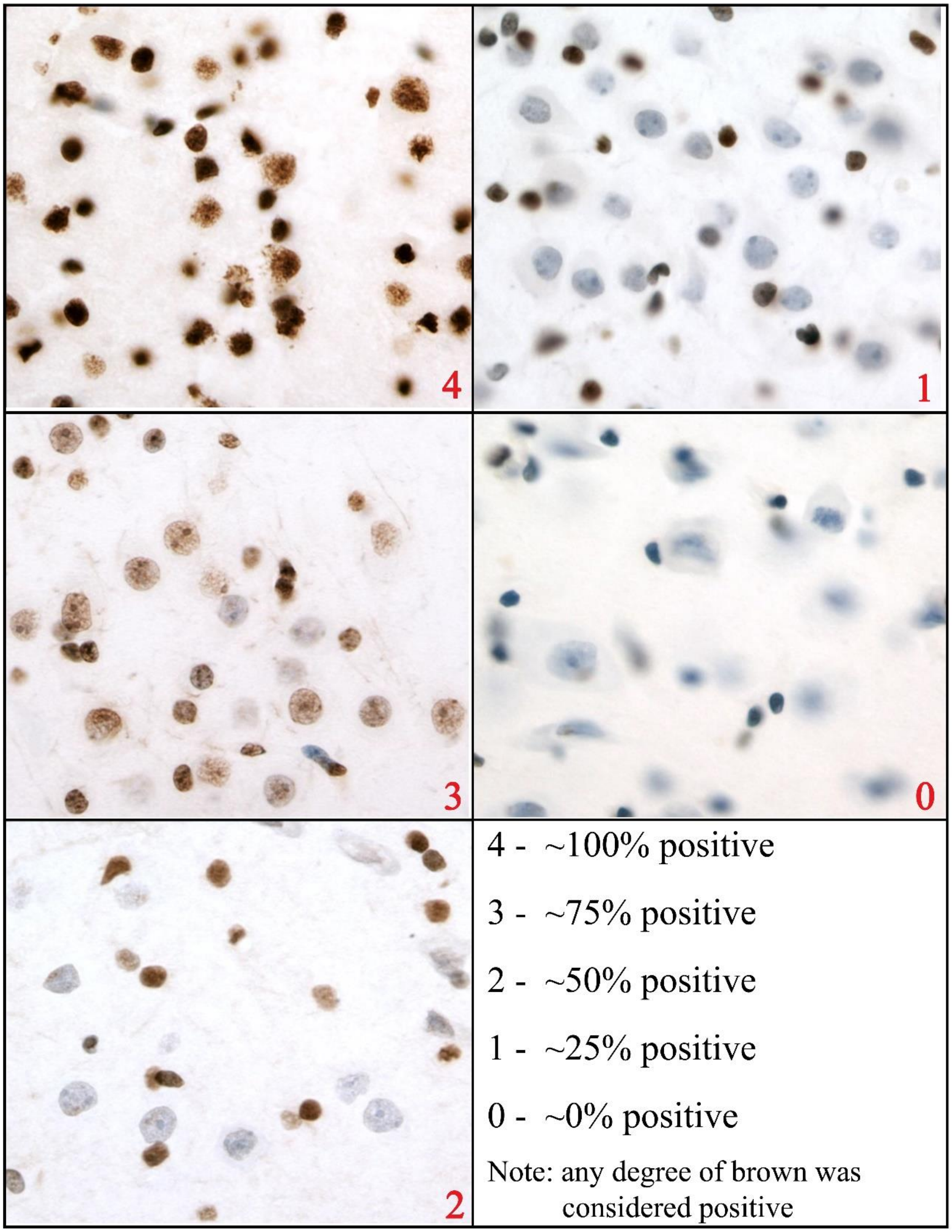

Figure S2: Standard scale images used for grading the intensity of immunoreactive nuclei. Strong labeling (3) was almost black with no nuclear details visible (arrows). Medium labeling (2) is distinctly brown but with chromatin stippling or nucleolus showing (arrows). Faint labeling (1) has brown coloration (arrows) that only barely obscures the blue staining in negative (0) cells (arrowheads). For grading, the majority level was the score assigned. Images taken at x 400 magnification. DAB detection of antibody (brown) and hematoxylin counterstain (blue).

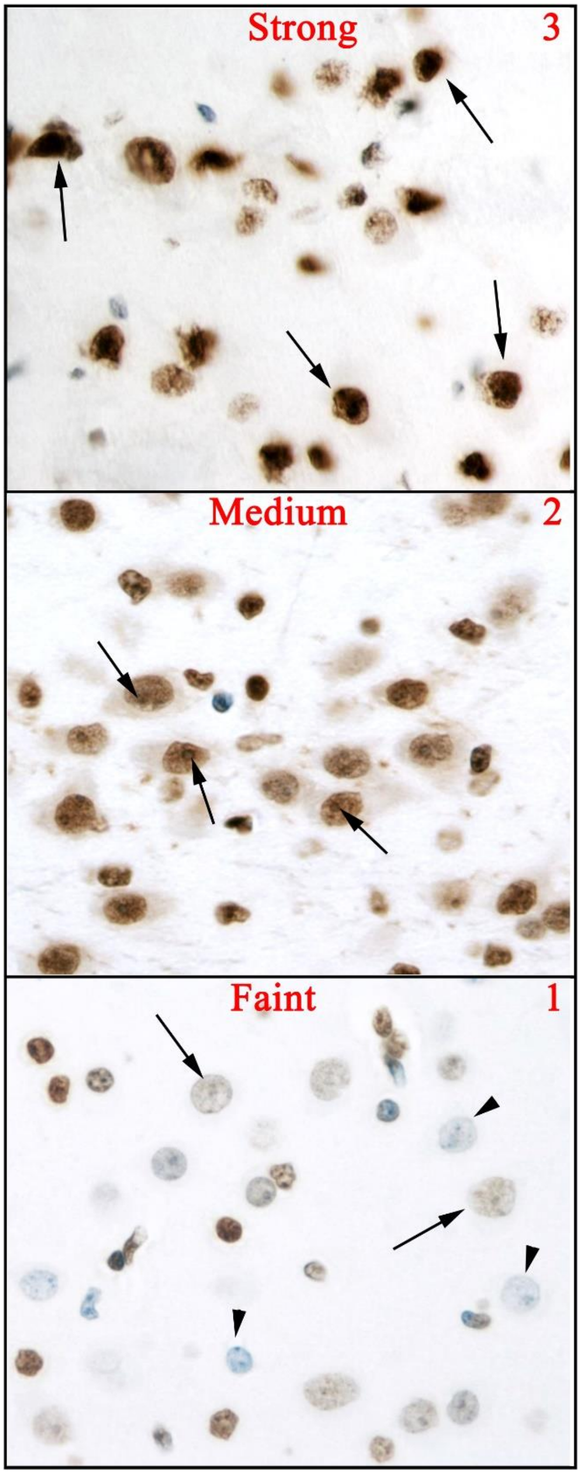

Figure S3: Cell type (probable) based on nuclear morphology

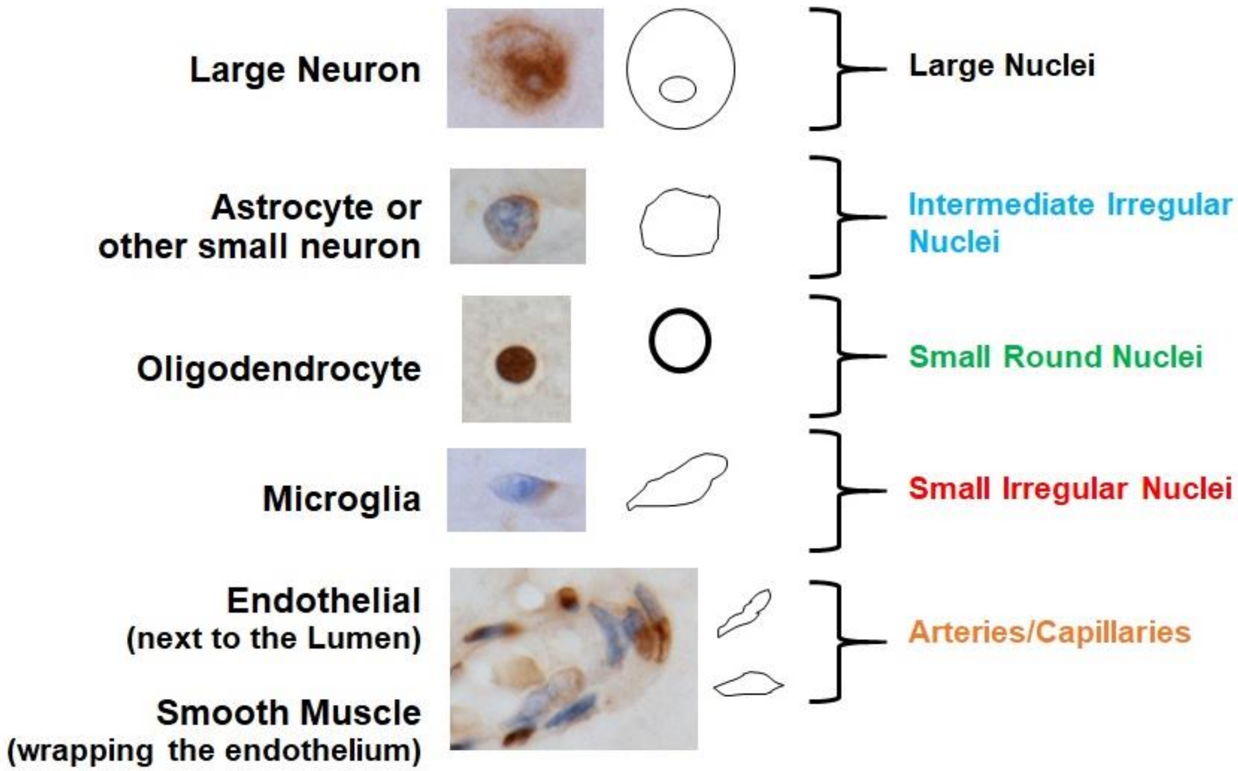

Supplement: Supplementary file 13 — Figure S1. Standard scale images used for grading the proportion of immunoreactive nuclei. Any degree of brown was considered positive. Photomicrographs show: 4 - ~100% positive; 3 - ~75% positive; 2 - ~50% positive; 1 <25% positive; 0 - ~0% positive. Images taken at × 400 magnification. DAB detection of antibody (brown) and hematoxylin counterstain (blue). Figure S2. Standard scale images used for grading the intensity of immunoreactive nuclei. Strong labeling (3) was almost black with no nuclear details visible (arrows). Medium labeling (2) is distinctly brown but with chromatin stippling or nucleolus showing (arrows). Faint labeling (1) has brown coloration (arrows) that only barely obscures the blue staining in negative (0) cells (arrowheads). For grading, the majority level was the score assigned. Images taken at × 400 magnification. DAB detection of antibody (brown) and hematoxylin counterstain (blue). Figure S3. Cell type (probable) based on nuclear morphology. (PDF 4533 kb) [file 13148_2018_596_MOESM13_ESM.pdf]
